# Supplementary figures and images for: BAG6 inhibits influenza A virus replication by inducing viral polymerase subunit PB2 degradation and perturbing RdRp complex assembly
Source: PLoS Pathog. 2024 Mar 18;20(3):e1012110. doi: 10.1371/journal.ppat.1012110 (PMC10977894; doi:10.1371/journal.ppat.1012110)

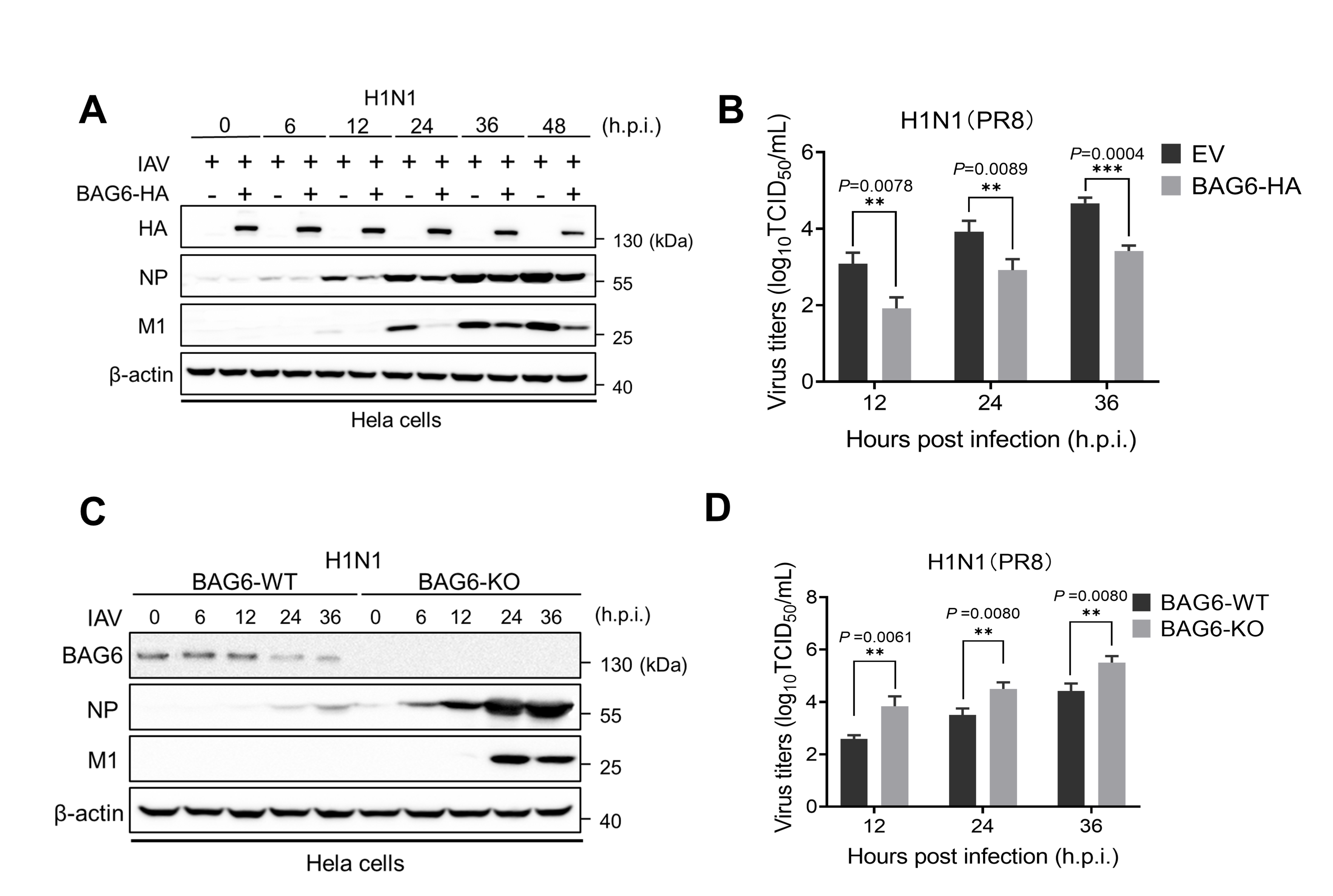

Supplement: S1 Fig — (A and B) HeLa cells were transfected with BAG6-HA or Empty vector (EV) plasmids. At 24 h post-transfection, the cells were infected with IAV H1N1 (MOI = 1.0). The viral NP expression was measured by western blotting at different time points postinfection, as indicated (A). Viral titers in the supernatants were determined by TCID50 assay at 12, 24 or 36 h post-infection (B). (C and D) BAG6-KO and BAG6-WT HeLa cells were infected with IAV H1N1 (MOI = 1.0), and the NP and M1 expression in the cell lysates (C) and viral titers in the supernatants (D) were determined by western blotting and TCID50 assay, respectively, at the indicated time points. Data presented as means ± SD and are representative of three independent experiments. *p < 0.05, **p < 0.01, ***p < 0.001, Unpaired Student’s t test. (TIF) [file ppat.1012110.s001.tif]

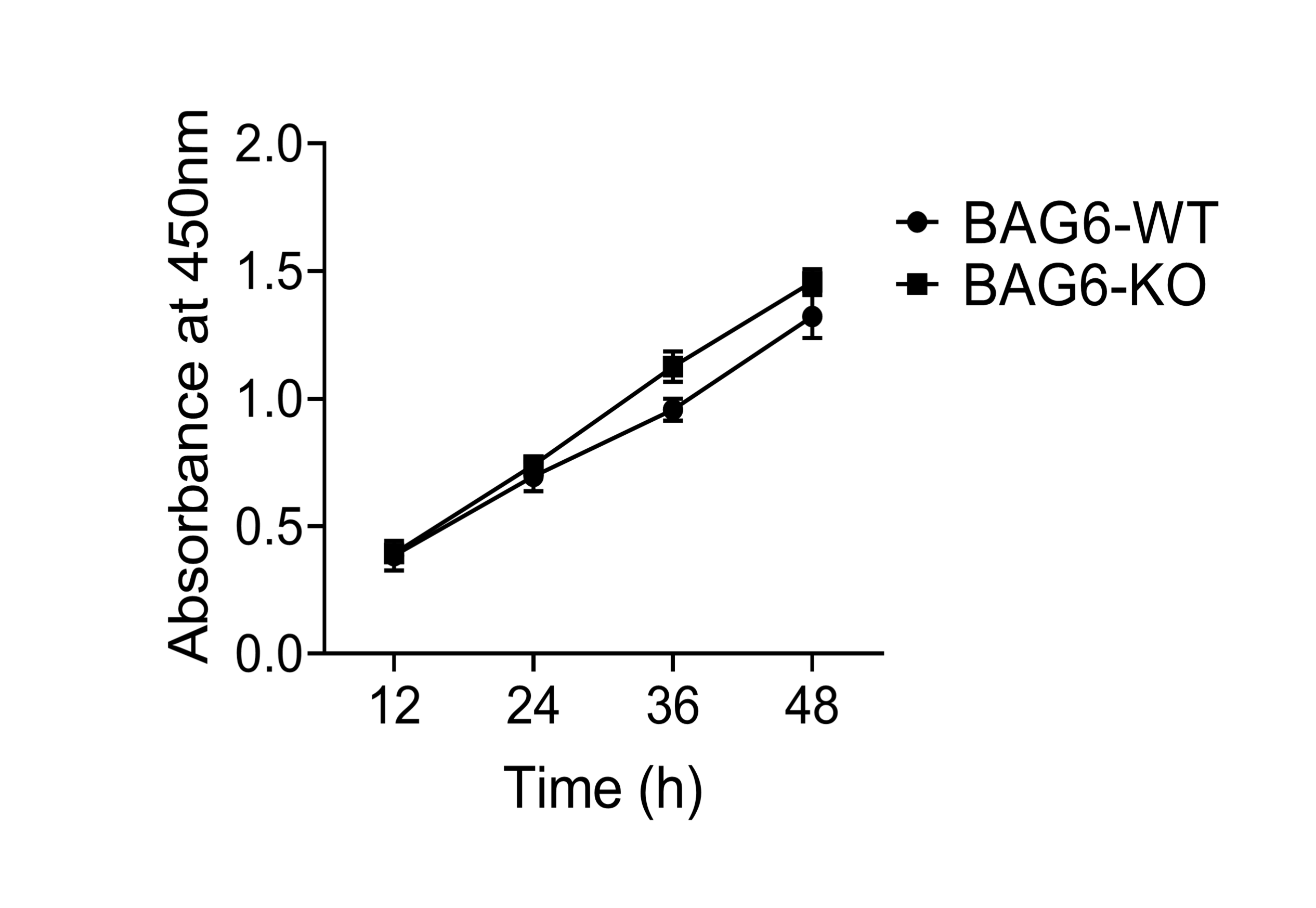

Supplement: S2 Fig — Cell viability of BAG6-KO A549 and BAG6-WT A549 cells was measured using the CCK8 kit. (TIF) [file ppat.1012110.s002.tif]

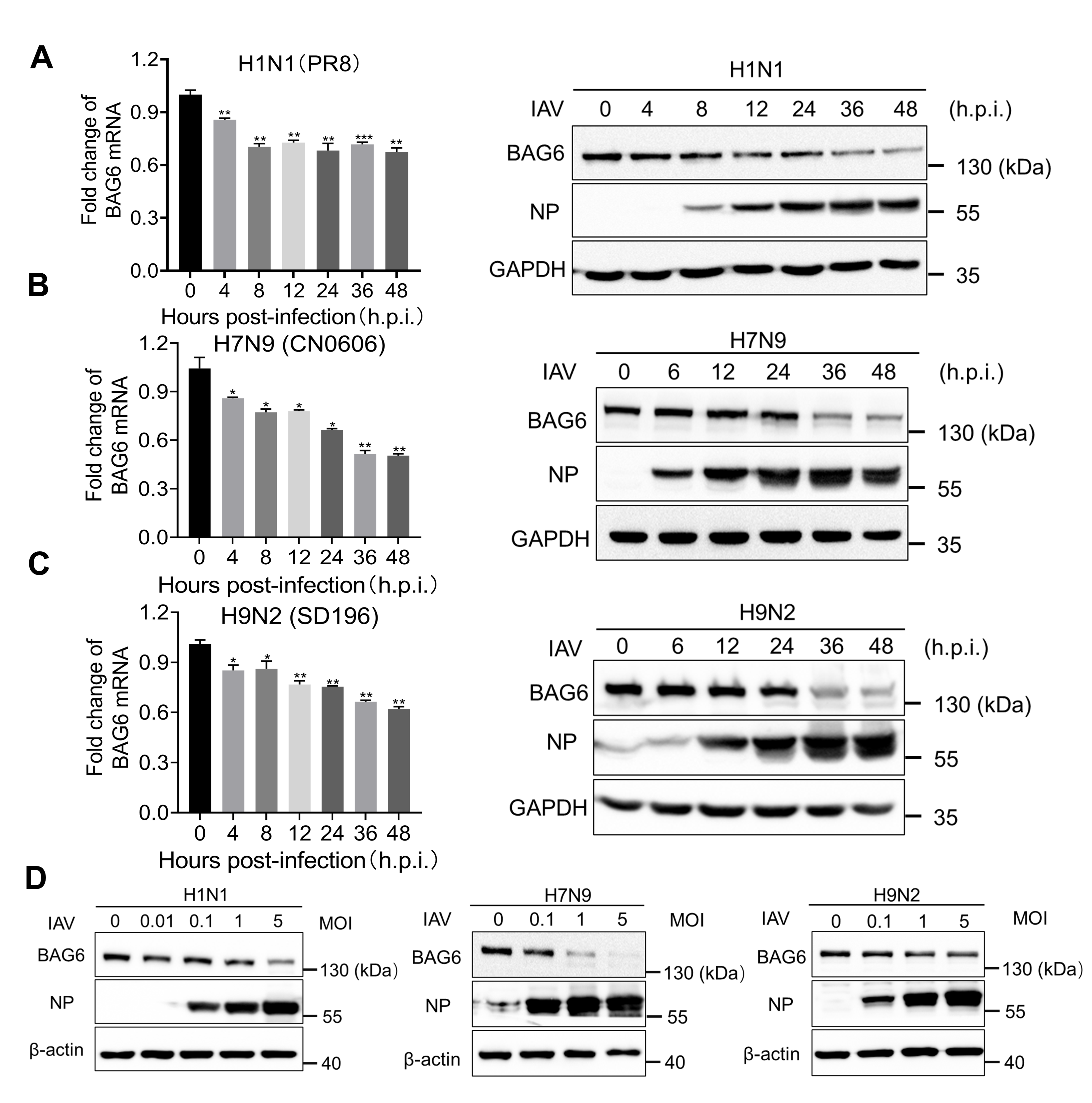

Supplement: S3 Fig — (A-C) A549 cells were infected with IAV H1N1 (A) or H7N9 (B) or H9N2 (C) (MOI = 1.0), and the mRNA and protein expression of BAG6 were determined by quantitative real-time PCR (left panels) and western blotting (right panels), respectively, at the indicated time points postinfection. (D) A549 cells were infected with IAV H1N1 or H7N9 or H9N2 with different MOI as indicated. The expression of BAG6 protein was determined by western blotting. Data presented as means ± SD and are representative of three independent experiments. *p < 0.05, **p < 0.01, ***p < 0.001, Unpaired Student’s t test. (TIF) [file ppat.1012110.s003.tif]

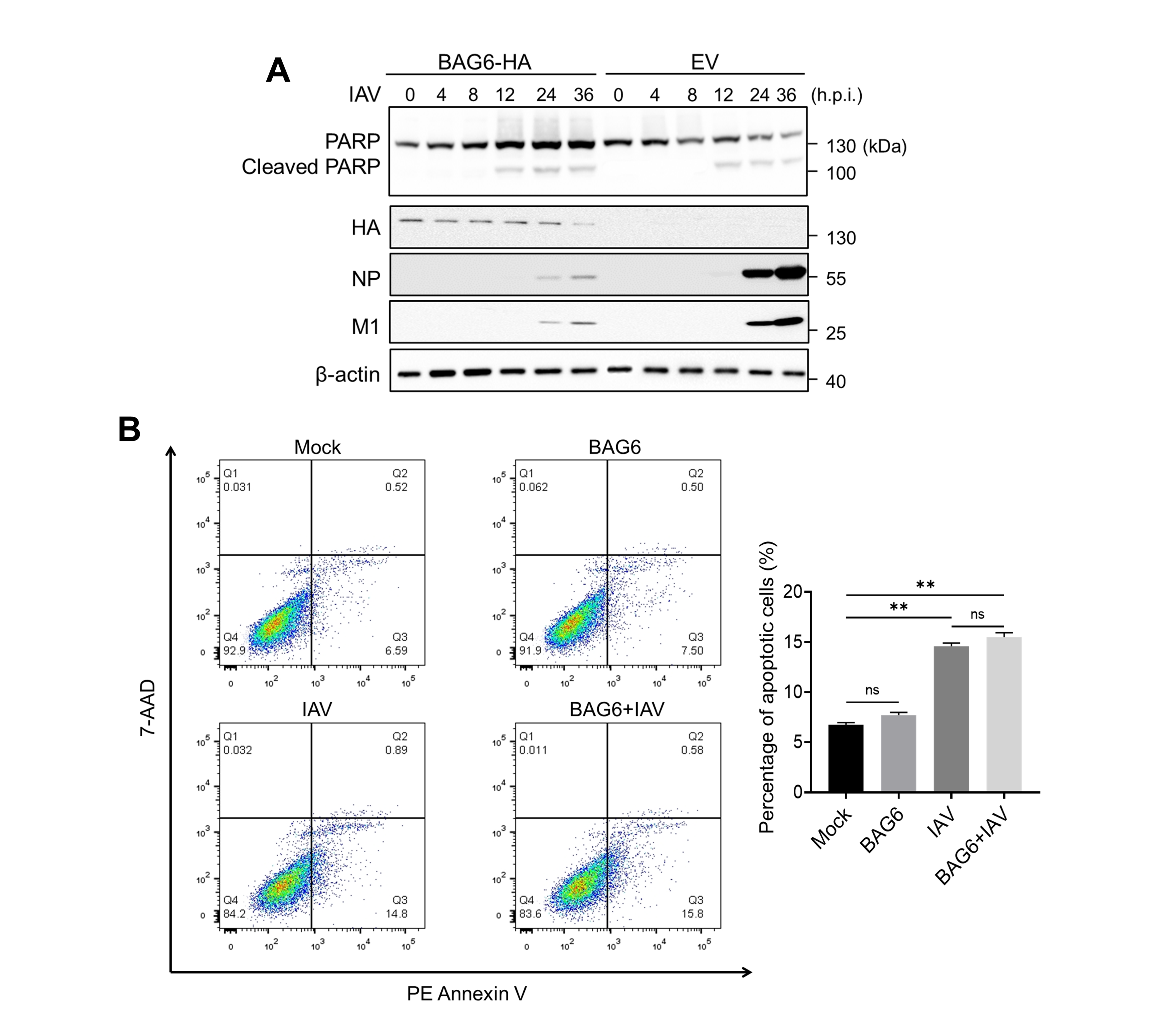

Supplement: S4 Fig — (A) BAG6-HA expression plasmid or empty vector was transfected into A549 cells for 24 h and then infected with PR8 virus at 1.0 MOI. The expression of PARP, cleaved PARP, and viral NP and M1 proteins at 0, 4, 8, 12, 24 and 36 h post-infection was detected using western blotting. β-actin detection was used as loading control. (B) A549 cells were untreated, transfected with BAG6 only, infected with IAV only, or both transfected with BAG6 and infected with IAV, and the cells were collected by centrifugation and were resuspended in 100 μL of 1x binding buffer supplemented with 5 μL of PE annexin V and 5 μL 7-AAD. Fluorescence of the stained cells was then analyzed using flow cytometry. Data presented as means ± SD and are representative of three independent experiments. *p < 0.05, **p < 0.01, ***p < 0.001, Unpaired Student’s t test. (TIF) [file ppat.1012110.s004.tif]
