# Supplementary material for: BAG6 inhibits influenza A virus replication by inducing viral polymerase subunit PB2 degradation and perturbing RdRp complex assembly
Source: PLoS Pathog. 2024 Mar 18;20(3):e1012110. doi: 10.1371/journal.ppat.1012110 (PMC10977894; doi:10.1371/journal.ppat.1012110)
Supplement: S1 Table — (DOCX) [file ppat.1012110.s005.docx]

**S1 Table. Amino acid residues at site 189 of the PB2 protein of different subtypes of influenza virus.**

| **Subtype** | **Host** | **Number of strains** | **Percentage** |
| --- | --- | --- | --- |
| H1 | Human | 10945^*^ | K (100%) |
|  | Avian | 1082^#^ | K (99.91%)  Q (0.09%) |
| H3 | Human | 7787^*^ | K (100%) |
|  | Avian | 2697^#^ | K (100%) |
| H5 | Human | 410^#^ | K (99.76%)  R (0.24%) |
|  | Avian | 16196^#^ | K (99.98%)  R (0.02%) |
| H7 | Human | 1262^#^ | K (100%) |
|  | Avian | 2941^#^ | K (99.97%)  R (0.03%) |
| H9 | Human | 63^#^ | K (100%) |
|  | Avian | 4789^#^ | K (99.96%)  R (0.04%) |

^*^only including the strains since Jan 2023 from Global Initiative on Sharing Avian Influenza Data (GISAID, [www.epicov.org)](http://www.epicov.org)) due to the too many sequences of human origin for subtypes H1 and H3.

^#^ including all the strains from GISAID.
