# Supplementary material for: BAG6 inhibits influenza A virus replication by inducing viral polymerase subunit PB2 degradation and perturbing RdRp complex assembly
Source: PLoS Pathog. 2024 Mar 18;20(3):e1012110. doi: 10.1371/journal.ppat.1012110 (PMC10977894; doi:10.1371/journal.ppat.1012110)
Supplement: S2 Table — (DOCX) [file ppat.1012110.s006.docx]

**S2 Table. List of primer pairs used in this study.**

| **Gene** | **Forward primer region (5’-3’)** | **Reverse primer region (5’-3’)** |
| --- | --- | --- |
| **reverse transcription PCR** | | |
| BAG6-HA | GTTCCTGACTATGCGGGCGGATCCATGGAGCCCAATGATA | AGTTGGGCCATGGCGGCCAAGCTTCTAGGGATCTTCAGCA |
| BAG6 1:92-HA | GTTCCTGACTATGCGGGCGGATCCATGGAGCCTAATGATAGTACCAGTACC | AGTTGGGCCATGGCGGCCAAGCTTCTAGTCTGAGGAGGAGCCCGTTCCA |
| BAG6 92:255-HA | GTTCCTGACTATGCGGGCGGATCCTCACCTCCCTTCTGGGGCATC | AGTTGGGCCATGGCGGCCAAGCTTCTAAGGTGTTGGGCCCGCTG |
| BAG6 1:255-HA | GTTCCTGACTATGCGGGCGGATCCATGGAGCCTAATGATAGTACCAGTACC | AGTTGGGCCATGGCGGCCAAGCTTCTAAGGTGTTGGGCCCGCTG |
| BAG6 1:753-HA | GTTCCTGACTATGCGGGCGGATCCATGGAGCCTAATGATAGTACCAGTACC | AGTTGGGCCATGGCGGCCAAGCTTCTAGCACAGAAGAGAAAGCAAGGCCC |
| BAG6 482:753-HA | GTTCCTGACTATGCGGGCGGATCCATGCTGGGACAGCAGGTGCCAG | AGTTGGGCCATGGCGGCCAAGCTTCTAGCACAGAAGAGAAAGCAAGGCCC |
| BAG6 482:1132-HA | GTTCCTGACTATGCGGGCGGATCCATGCTGGGACAGCAGGTGCCAG | AGTTGGGCCATGGCGGCCAAGCTTCTAAGGATCATCAGCAAAGGCCCG |
| BAG6 641:1132-HA | GTTCCTGACTATGCGGGCGGATCCATGACTGACTTCTTGCAGGCAACAC | AGTTGGGCCATGGCGGCCAAGCTTCTAAGGATCATCAGCAAAGGCCCG |
| BAG6 del124:186-HA | GGGGGCCTGGGGCCTCTGGAGGGCCCCAACCGCAGCACAGTC | TGCGGTTGGGGCCCTCCAGAGGCCCCAGGCCCCCGAGTACCA |
| PB2-Flag | GACGACGATGACAAGGGATCCATGGAAAGAATAAAAGAACTACGAA | TGGGCCATGGCGGCCAAGCTTCTAATTGATGGCCATCCGAATTCTTTTG |
| PB1-Flag | GACGACGATGACAAGGGATCCATGGATGTCAATCCGACCT | TGGGCCATGGCGGCCAAGCTTCTATTTTTGCCGTCTGAGC |
| PA-Flag | GACGACGATGACAAGGGATCCATGGAAGATTTTGTGCGAC | TGGGCCATGGCGGCCAAGCTTCTAACTCAATGCATGTGTAA |
| NP-Flag | GACGACGATGACAAGGGATCCATGGCGTCCCAAGGCACCA | TGGGCCATGGCGGCCAAGCTTTTAATTGTCGTACTCCTCT |
| M1-Flag | GACGACGATGACAAGGGATCCATGAGTCTTCTAACCGAGG | TGGGCCATGGCGGCCAAGCTTTCACTTGAACCGTTGCAT |
| N-terminal PB2-Flag | GACGACGATGACAAGGGATCCATGGAAAGAATAAAAGAACTACGAA | TGGGCCATGGCGGCCAAGCTTCTATCCTGGAGTATACATCTGTTCCCA |
| PB2-K5R-Flag | ATGGAAAGAATAAGAGAACTAAGAAATCTAATGTCGCAG | CTAATTGATGGCCATCCGAATTCTTTTGGTC |
| PB2-K33R-Flag | CAAGAGGTACACATCAGGAAGACAGGAGAAGA | CTGATGTGTACCTCTTGATTATGGCCATATGGTCCA |
| PB2-K41R-Flag | AAGACAGGAGAGGAACCCAGCACTTAGGATGAAATG | GGTTCCTCTCCTGTCTTCCTGATGTGTACTTCT |
| PB2-K48R-Flag | TTAGGATGAGATGGATGATGGCAATGAAATATCCA | CATCATCCATCTCATCCTAAGTGCTGGGTTCTTCTC |
| PB2-K61R-Flag | AGCAGACAGGAGGATAACGGAAATGATTCCTGAG | TTATCCTCCTGTCTGCTGTAATTGGATATTTCATTGC |
| PB2-K187R-Flag | CGATAACCAGAGAGAAGAAAGAAGAACTCCAGGATTG | CTTCTCTCTGGTTATCGTTAGTTGCGATTCCG |
| PB2-K189R-Flag | CAAAGAGAGGAAAGAAGAACTCCAGGATTGCAAAAT | GGAGTTCTTCTTTCCTCTCTTTGGTTATCGTTAGTTGCGA |
| PB2-K331R-Flag | CACATTTAGGAGAACAAGCGGATCATCAGTCA | CCGCTTGTTCTCCTAAATGTGAATCCACCAAAACTGAA |
| PB2-K339R-Flag | ATCAGTCAGGAGAGAGGAAGAGGTGCTTACGGG | CCTCTCTCCTGACTGATGATCCGCTTGTTCTCT |
| PB2-K353R-Flag | ACATTGAGGATAAGAGTGCATGAGGGATATGA | TGCACTCTTATCCTCAATGTTTGAAGATTTCCCGTA |
| PB2-K482R-Flag | AATCAGCAGAATGGGTGTAGATGAGTACTCCAGCA | CACCCATTCTGCTGATTCTCACTCCTCTCATTGA |
| PB2-K561R-Flag | ACTGTTAGAATTCAGTGGTCCCAGAACCCTA | ACCACTGAATTCTAACAGTTTCCCAGTTTCTGATGATC |
| PB2-K617R-Flag | GATAATAAGACTTCTTCCCTTCGCAGCCGCT | AAGGGAAGAAGTCTTATTATCTGTGCGGTATCAAATGTCC |
| PB2-K669R-Flag | TCTGGGCAGAGAAGACAAGAGATATGGGCCAG | TGTCTTCTCTGCCCAGAATGAGGAATCCCCTC |
| PB2-K721R-Flag | AGGAGAGAGGGCTAATGTGCTAATTGGGCAAGG | CATTAGCCCTCTCTCCTTTCGCAAGGTTGCTC |
| PB2-K736R-Flag | GGTAATGAGACGGAAACGGGACTCTAGCATAC | GTTTCCGTCTCATTACCAACACCACGTCTCCTT |
| PB2-K738R-Flag | ATGAAACGGAGACGGGACTCTAGCATACTTACTGACA | TCCCGTCTCCGTTTCATTACCAACACCACGTC |
| PB2-K752R-Flag | ATGGAAAGAATAAAAGAACTAAGAAATCTAATGTCGCAG | CTAATTGATGGCCATCCGAATTCTTCTGGTCGCTGTCTGG |
| **quantitative real-time PCR** | | |
| BAG6 | TGCTGCCTTCATACAACGCCTC | GTCCACCATAGAGAAGTTCTGGC |
| β-actin | CACCATTGGCAATGAGCGGTTC | AGGTCTTTGCGGATGTCCACG |
| M1 | CCATCAGGCCCCCTCAAAGCCGAGA | ACGGTGAGCGTGAACACGAACCCTA |
| NP | AGAGACGGAAAATGGGTGAGAGAGC | GGATCCATTCCAGTACGCACGAGAG |
