# Supplementary material for: BAG6 inhibits influenza A virus replication by inducing viral polymerase subunit PB2 degradation and perturbing RdRp complex assembly
Source: PLoS Pathog. 2024 Mar 18;20(3):e1012110. doi: 10.1371/journal.ppat.1012110 (PMC10977894; doi:10.1371/journal.ppat.1012110)
Supplement: S1 Data — (PDF) [file ppat.1012110.s007.pdf]

Original Images for Blots and IF

BAG6 inhibits influenza A virus replication by inducing viral polymerase subunit PB2 degradation and perturbing RdRp complex assembly

Yong Zhou, Tian Li, Yunfan Zhang, Nianzhi Zhang, Yuxin Guo, Xiaoyi Gao, Wenjing Peng, Sicheng Shu, Chuankuo Zhao, Di Cui, Honglei Sun, Yipeng Sun, Jinhua Liu, Jun Tang, Rui Zhang, and Juan Pu

Figure 1A

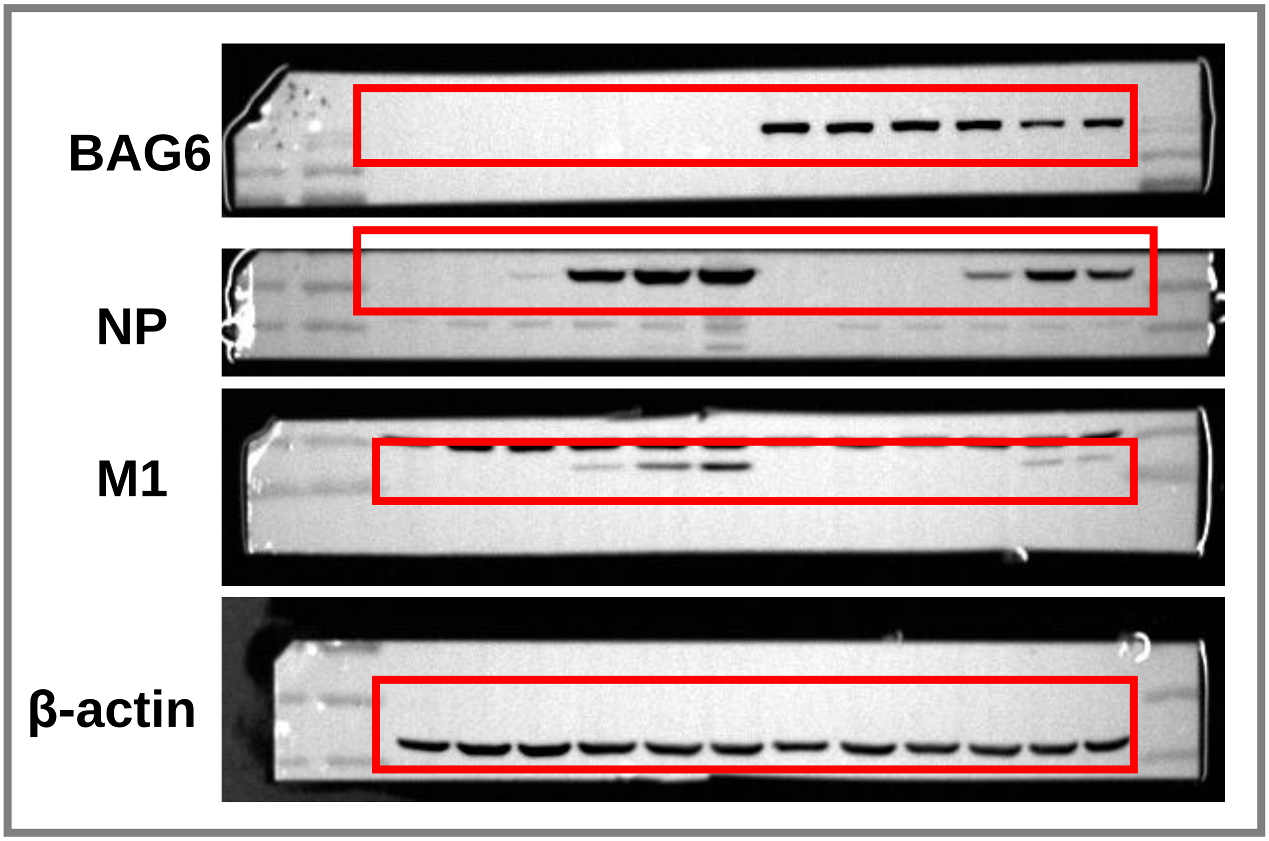

Figure 1C and 1E

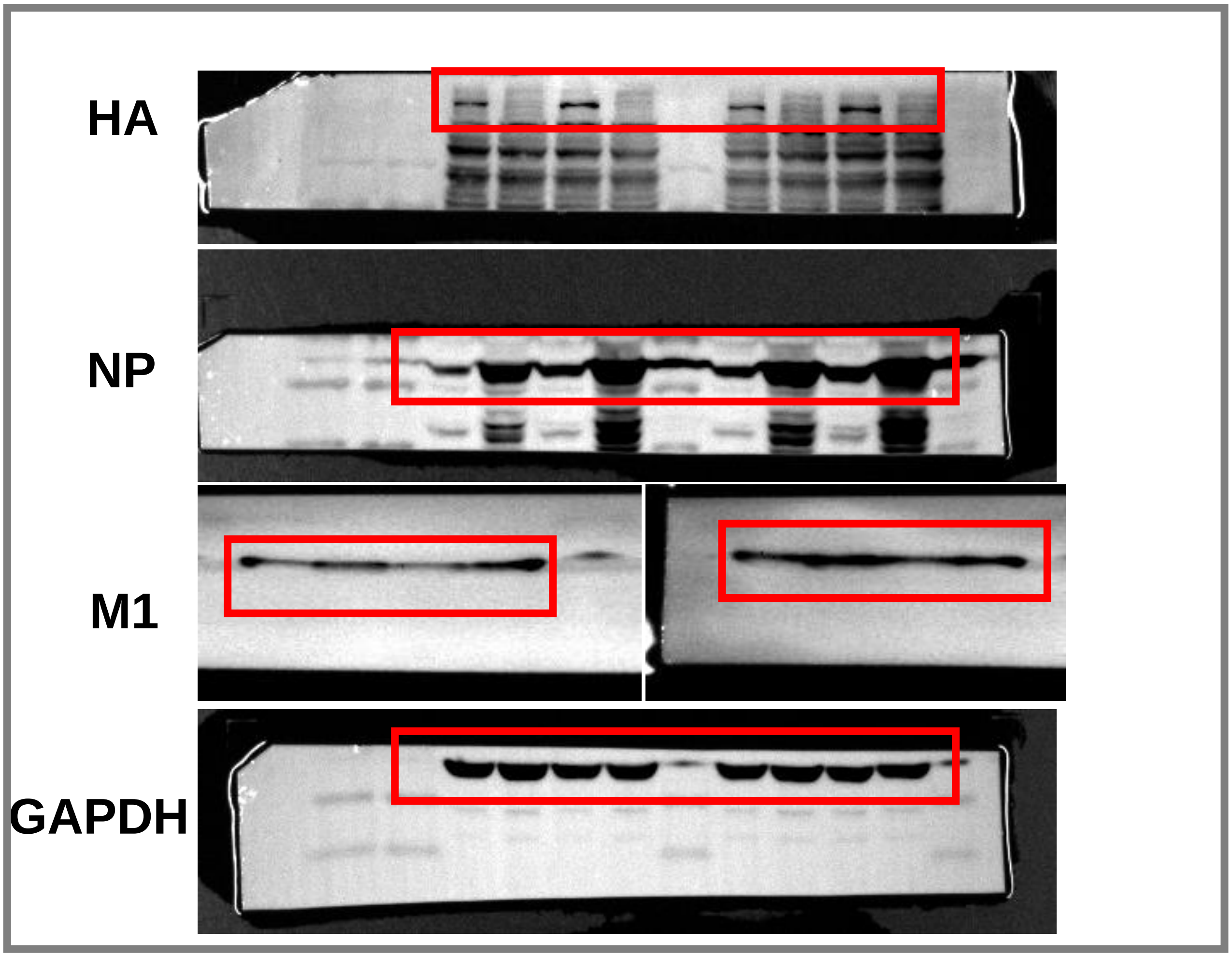

Figure 1G

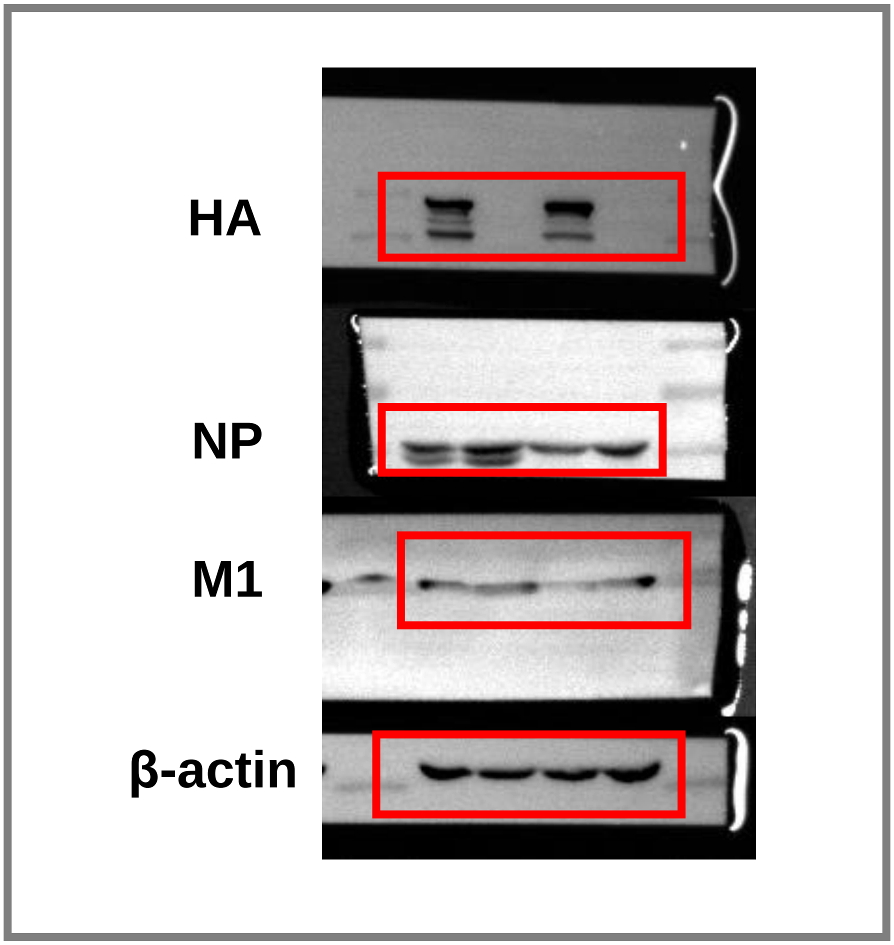

Figure 2A

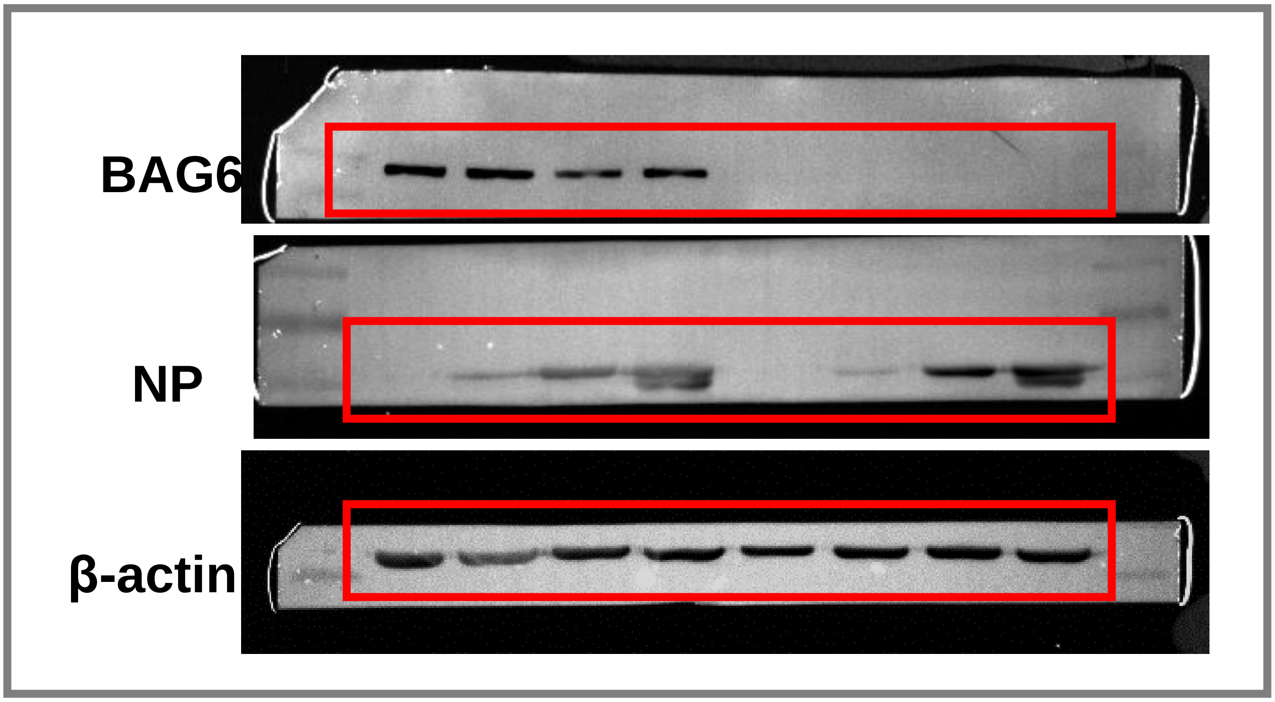

Figure 2C

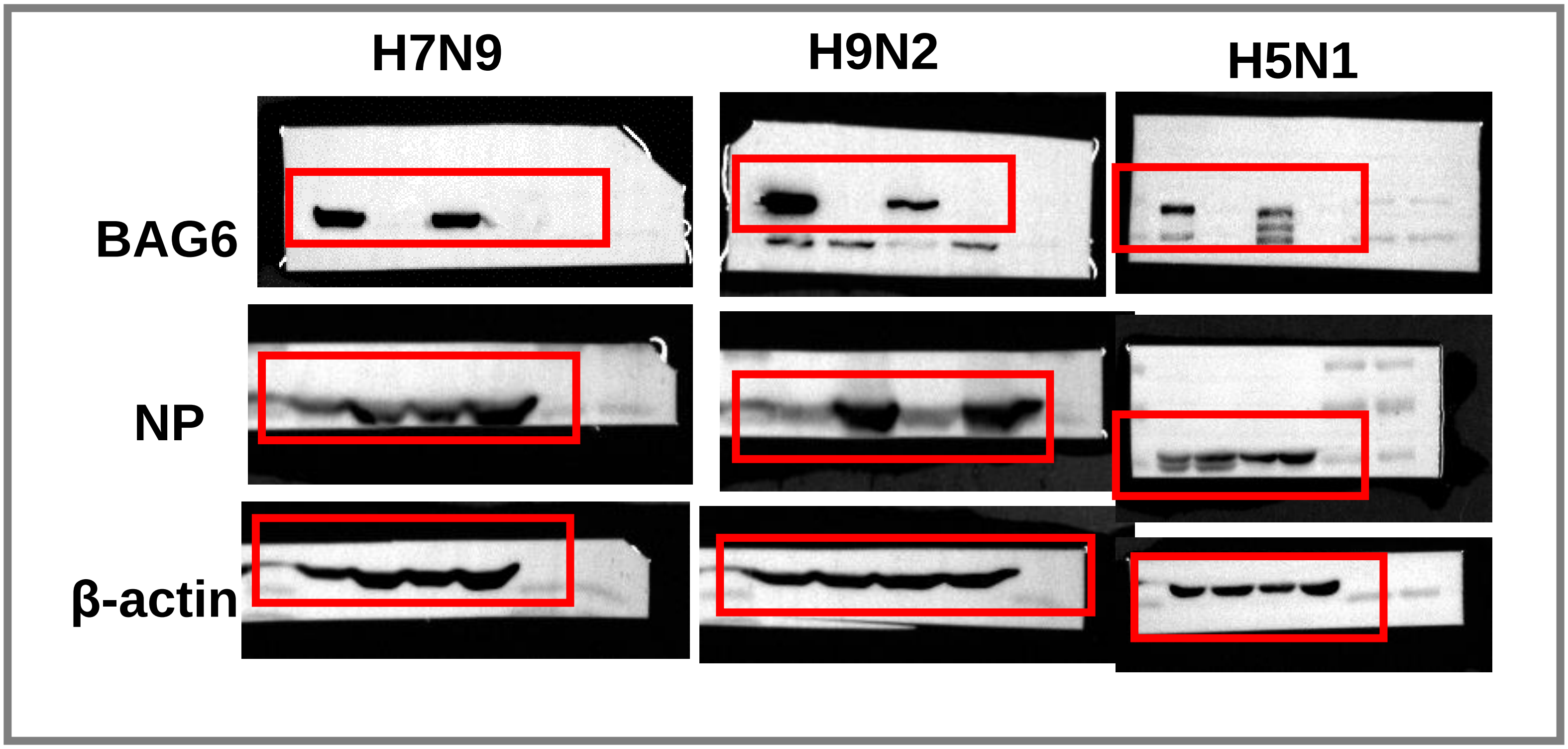

Figure 2E

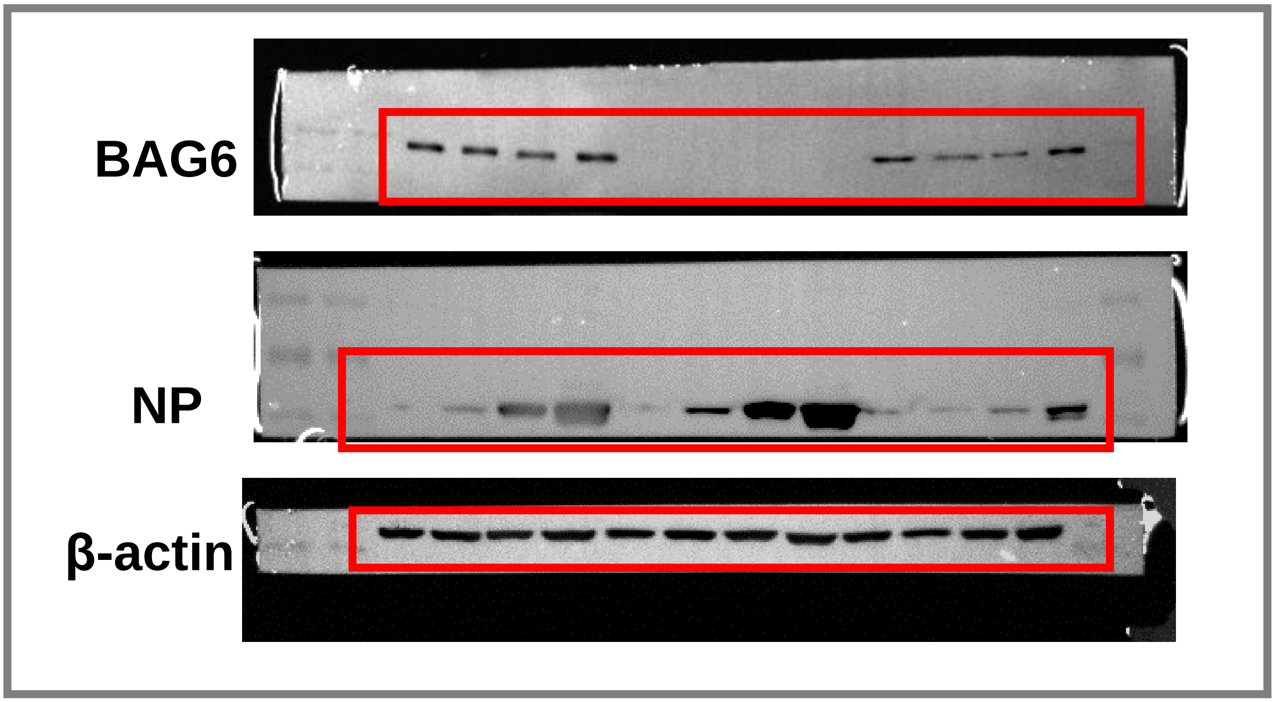

Figure 3B

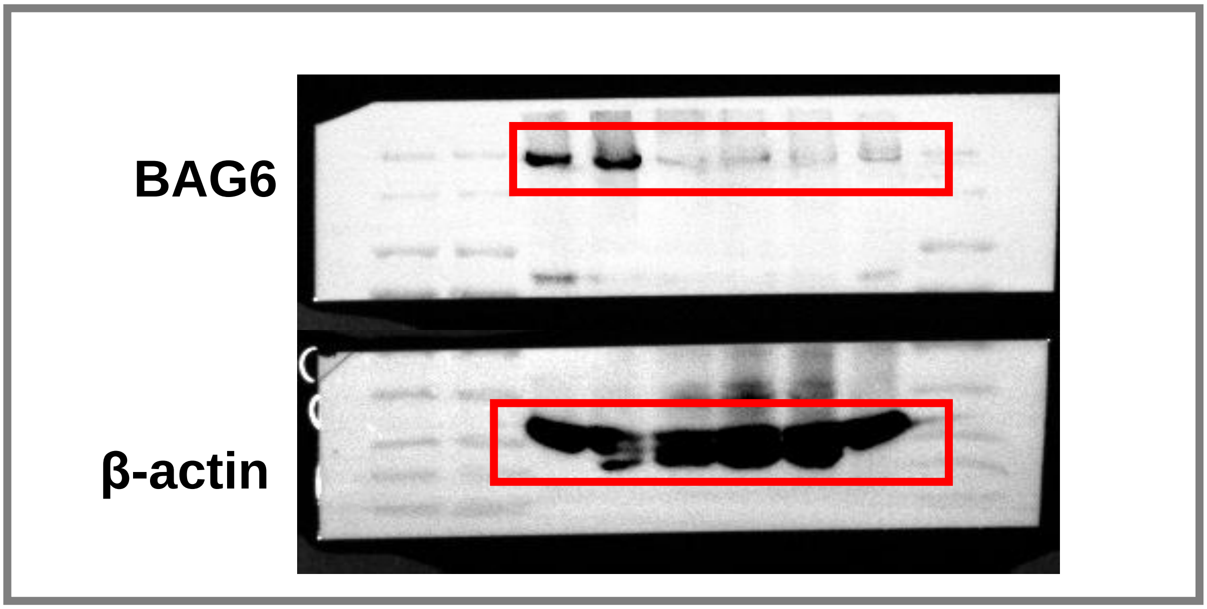

Figure 4A

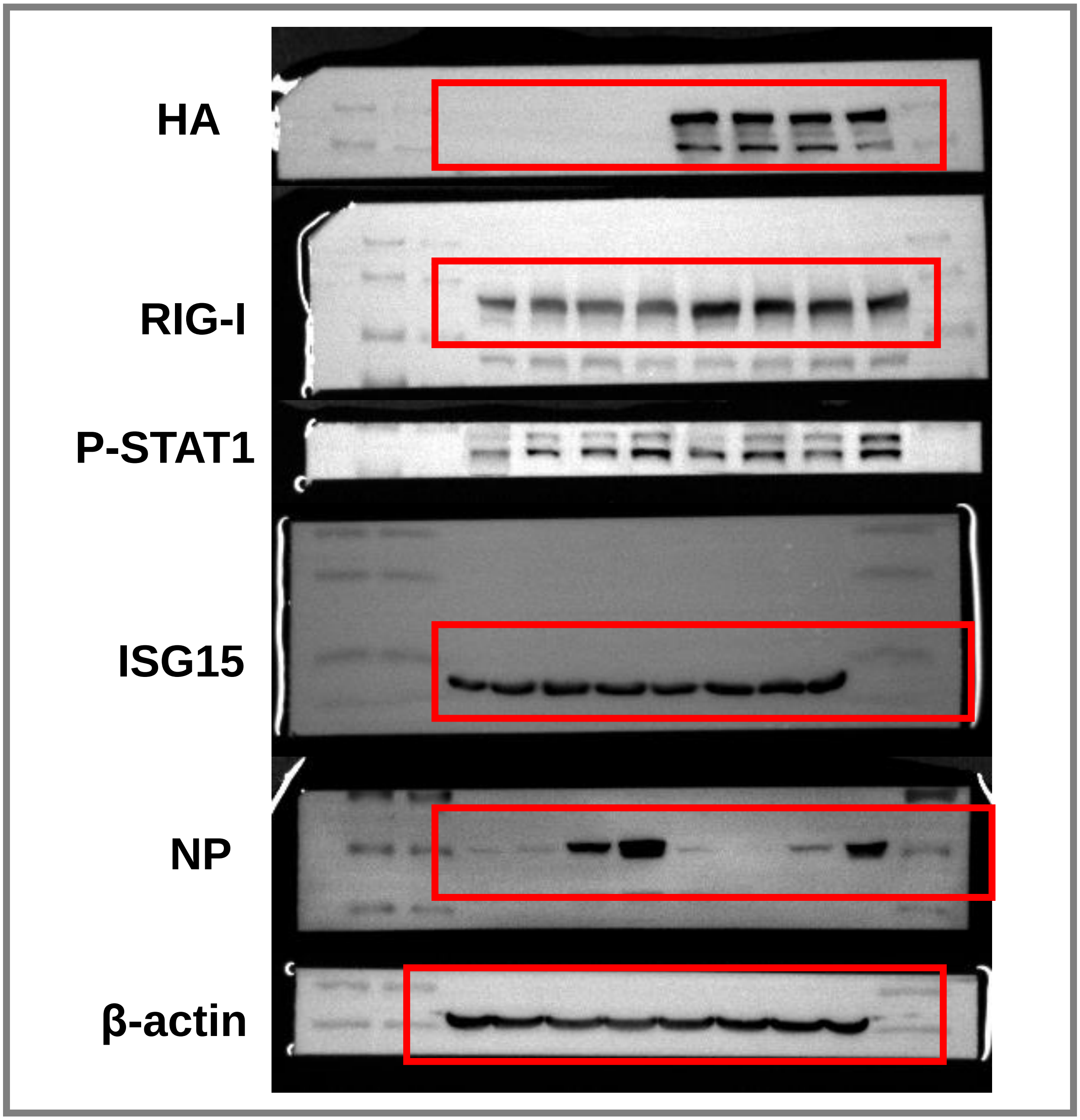

Figure 4B

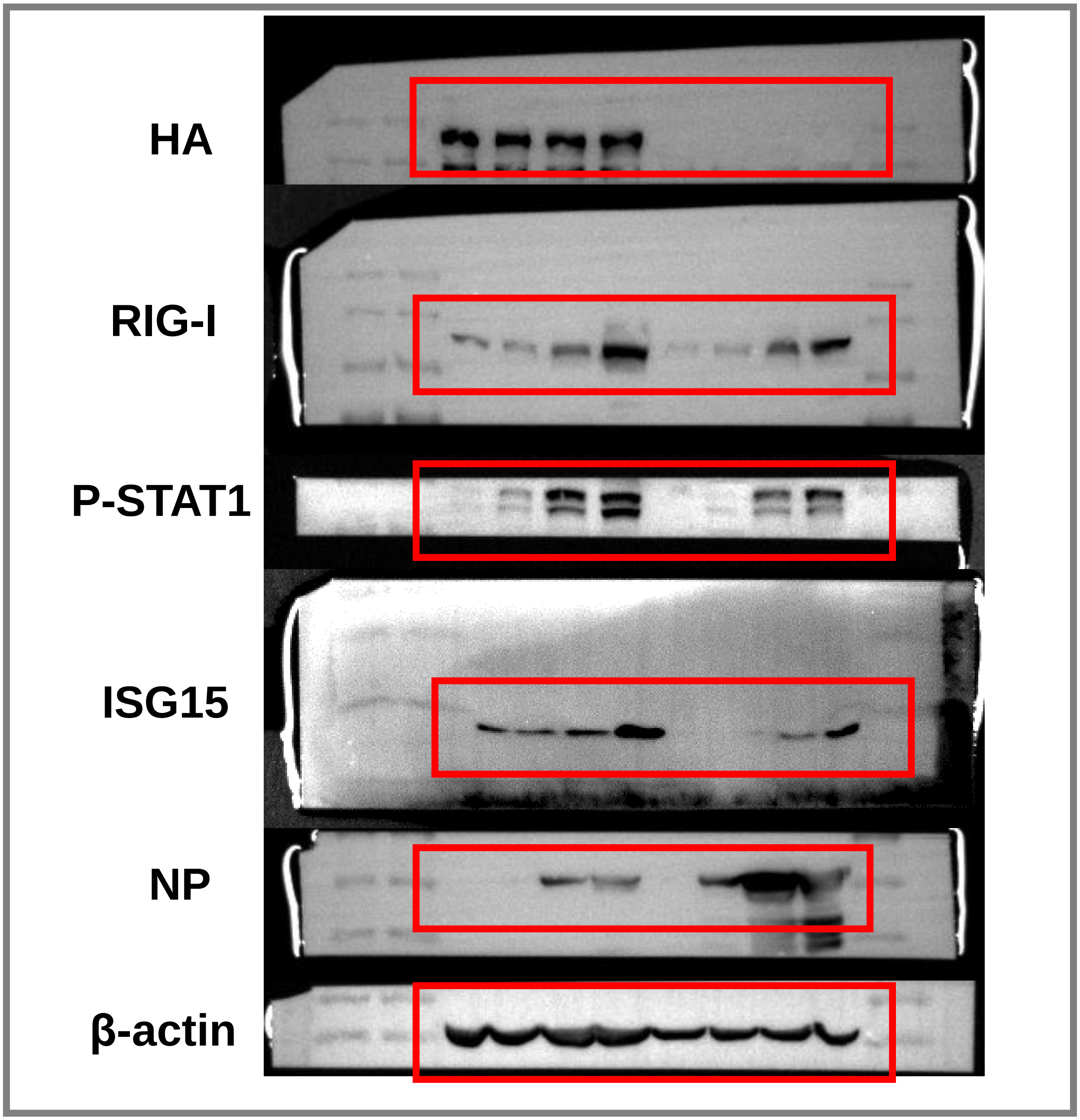

Figure 4C

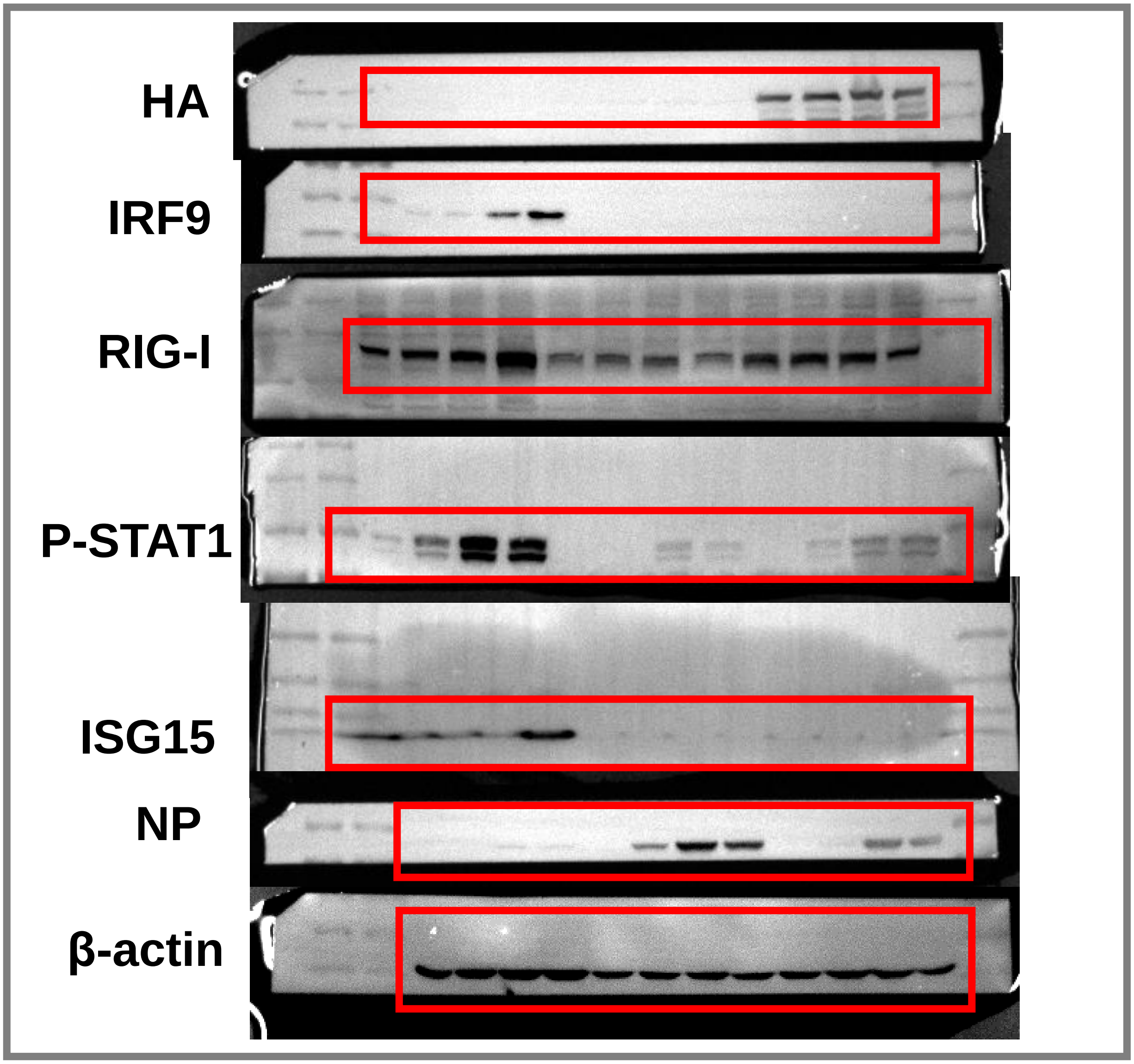

Figure 4E

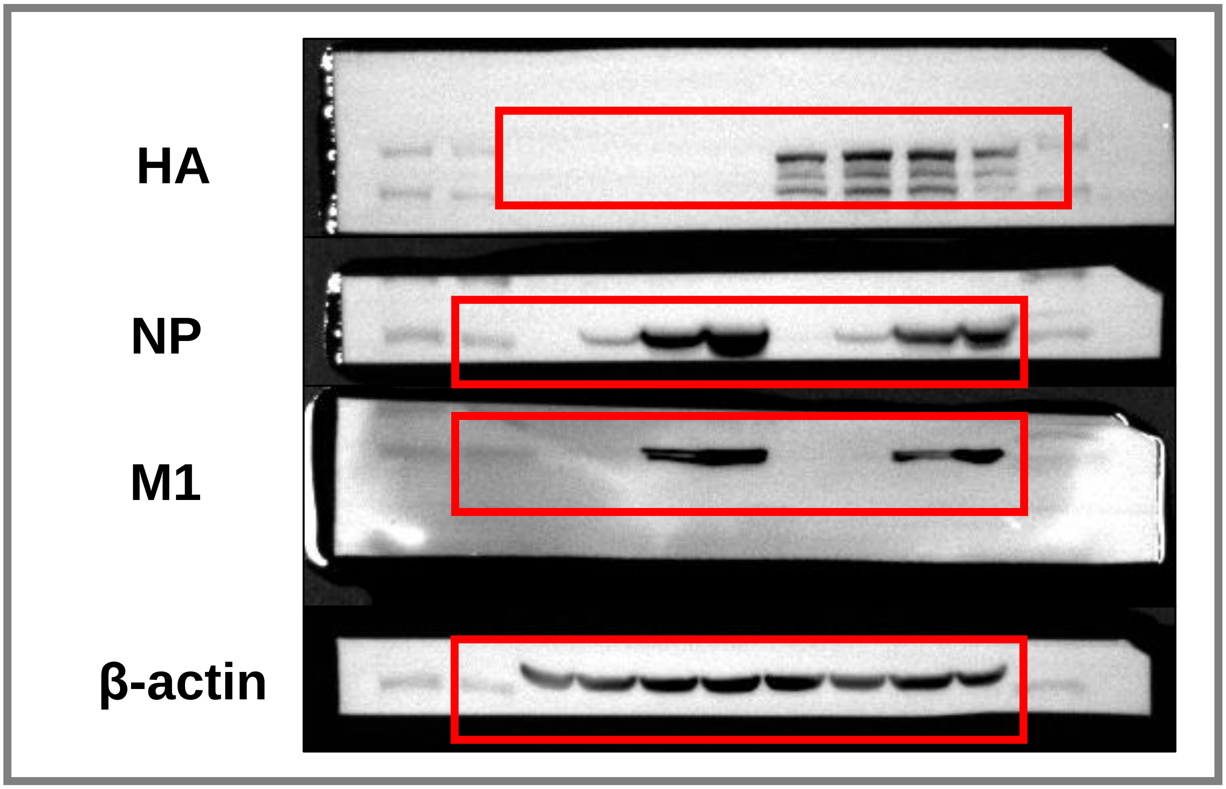

Figure 5A

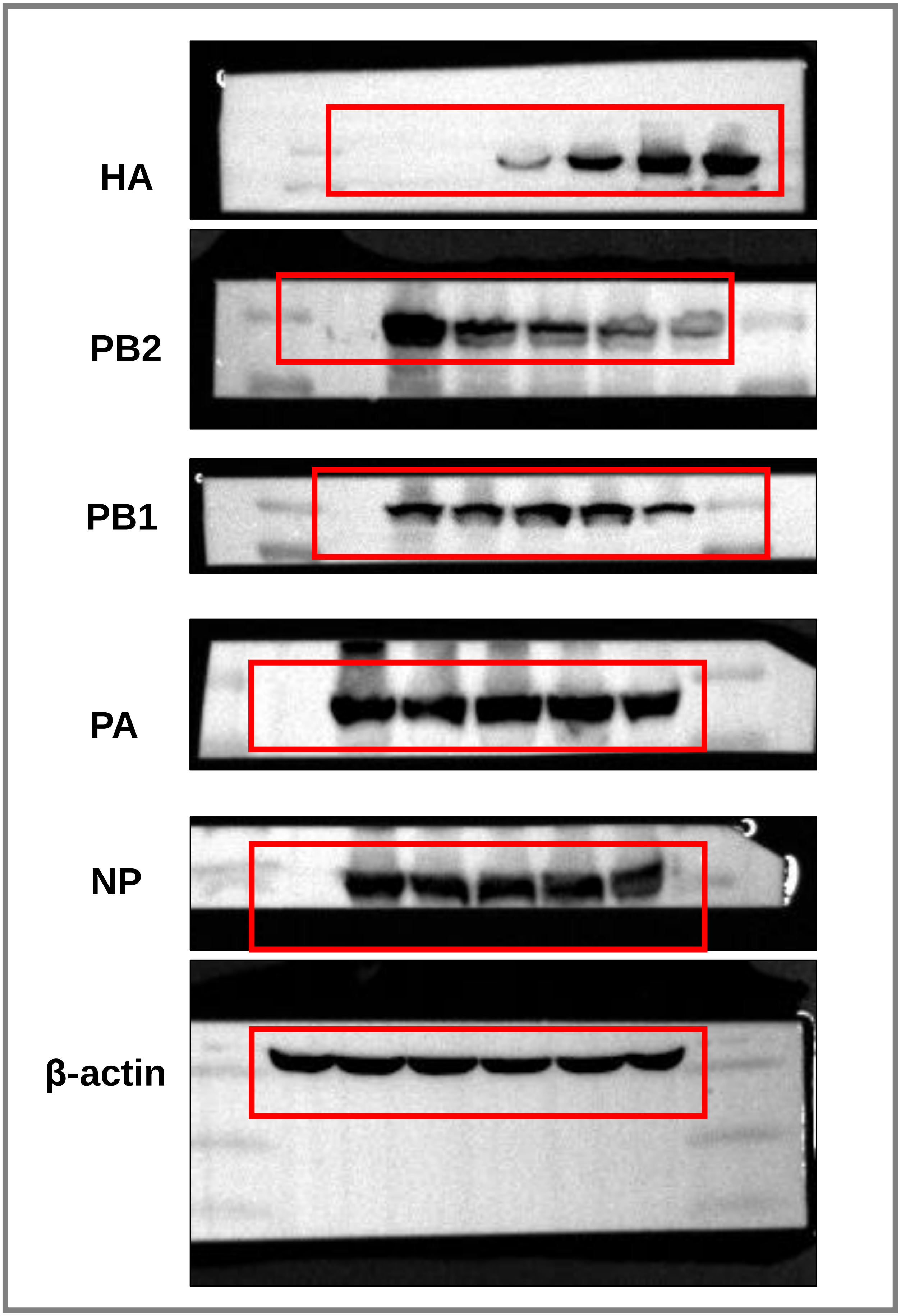

Figure 5A

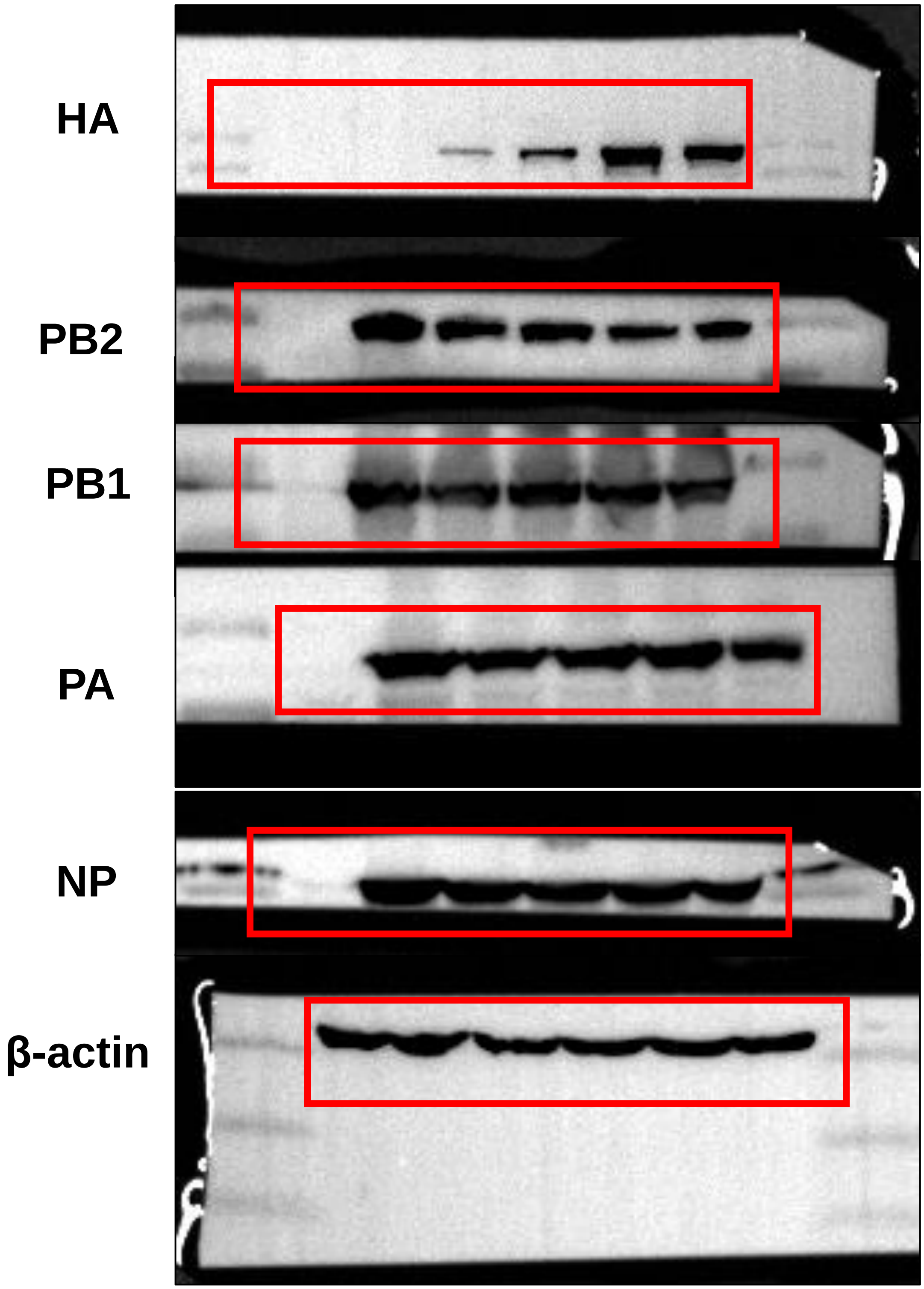

Figure 5A

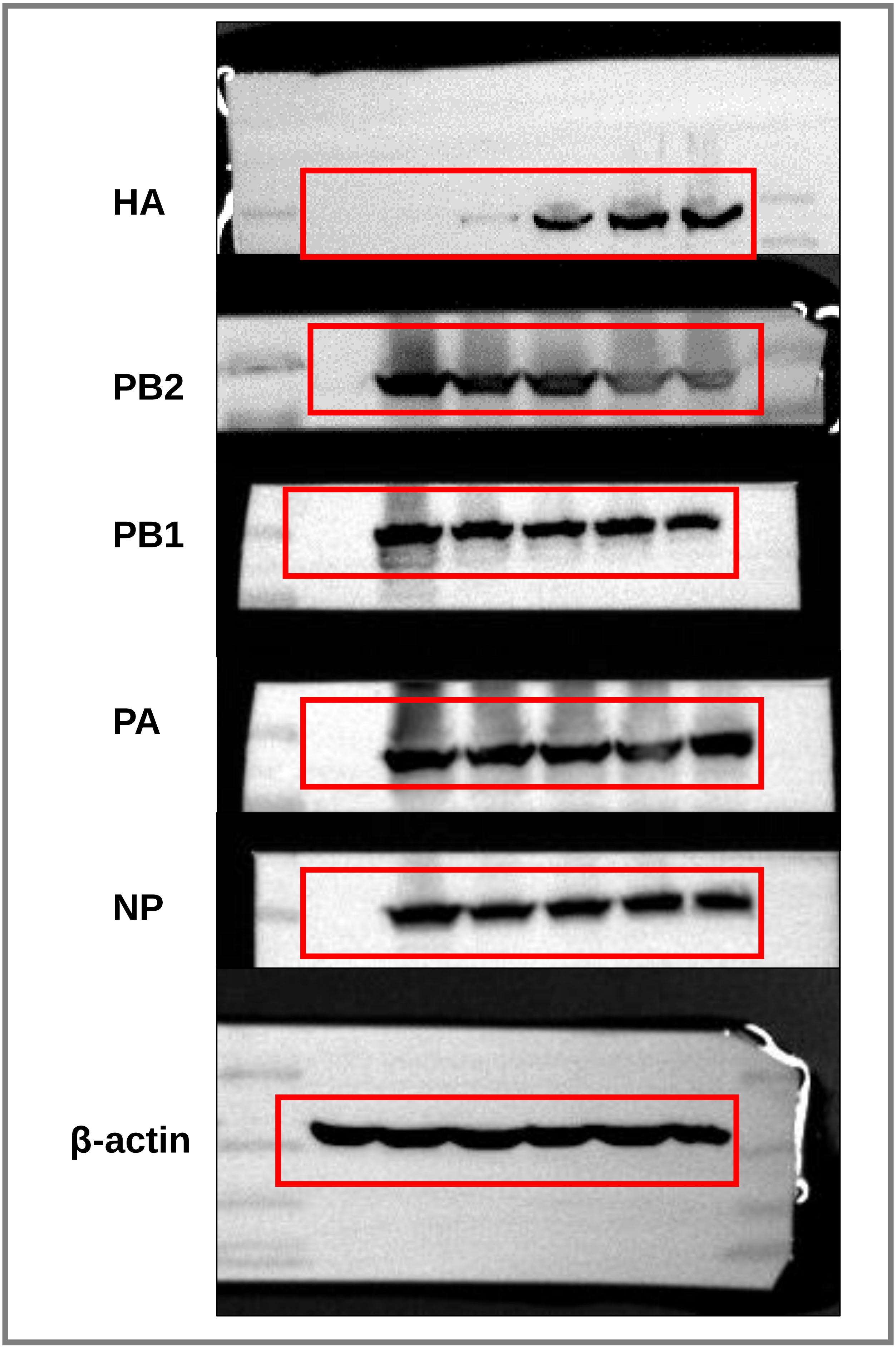

Figure 5B

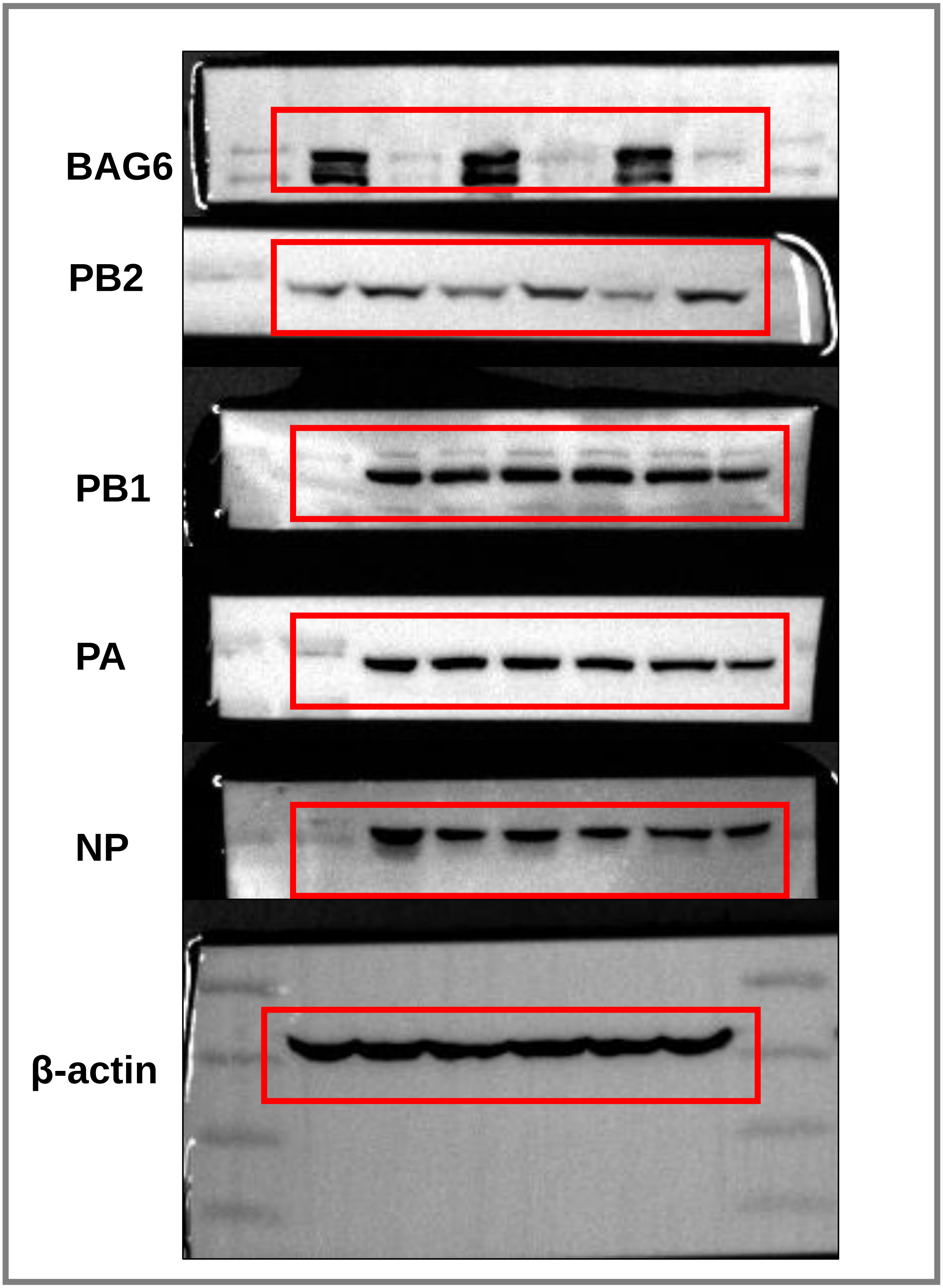

Figure 5C-5F

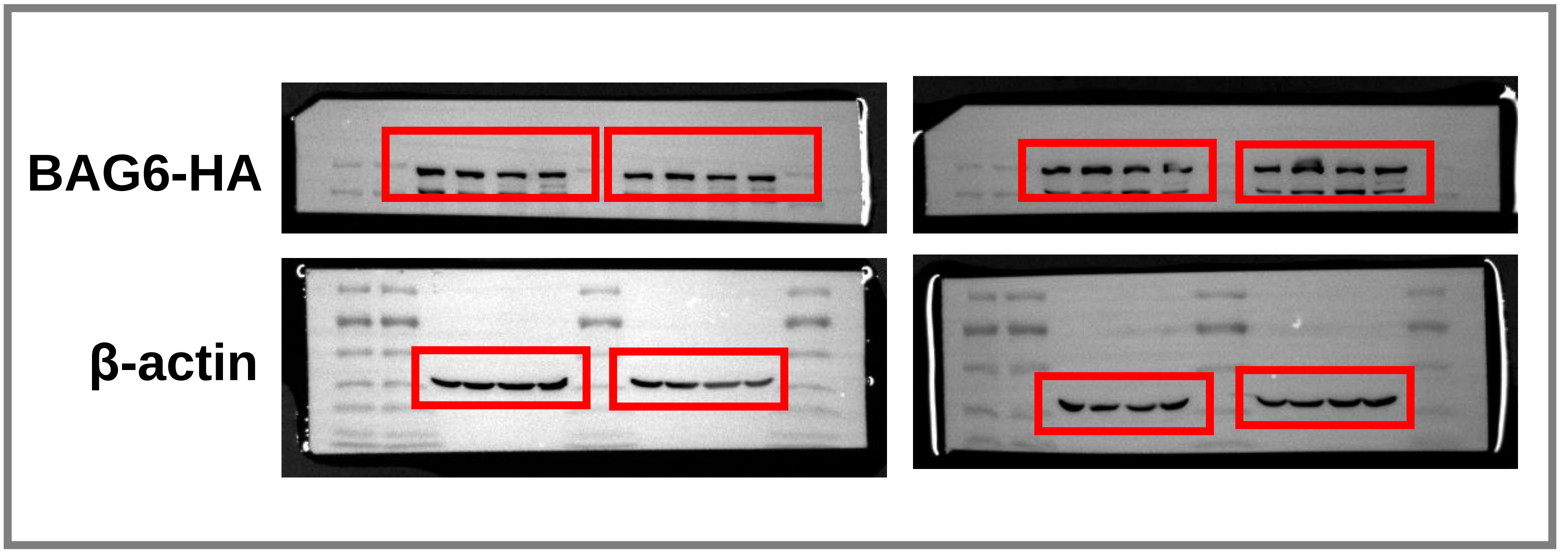

Figure 6A

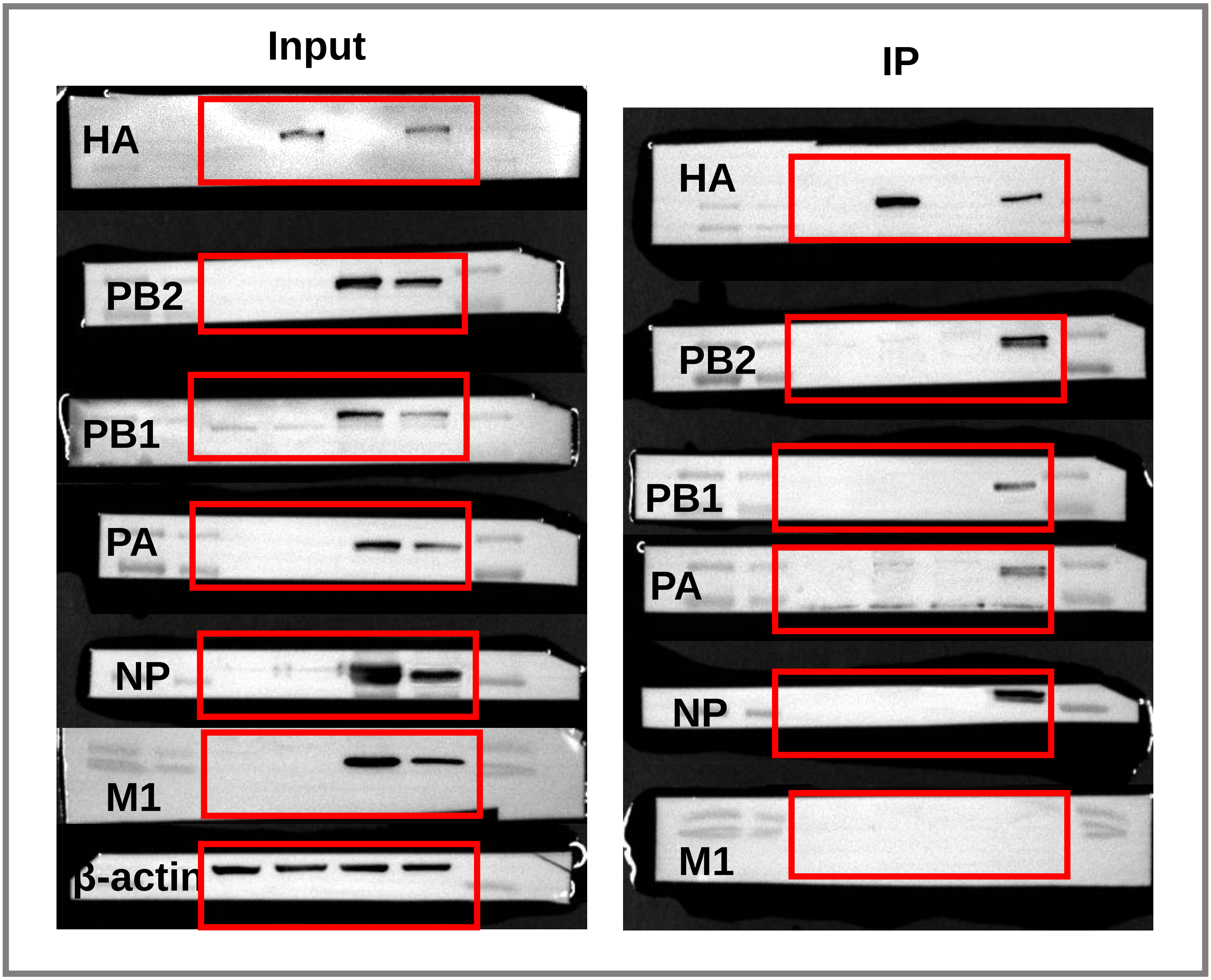

Figure 6B

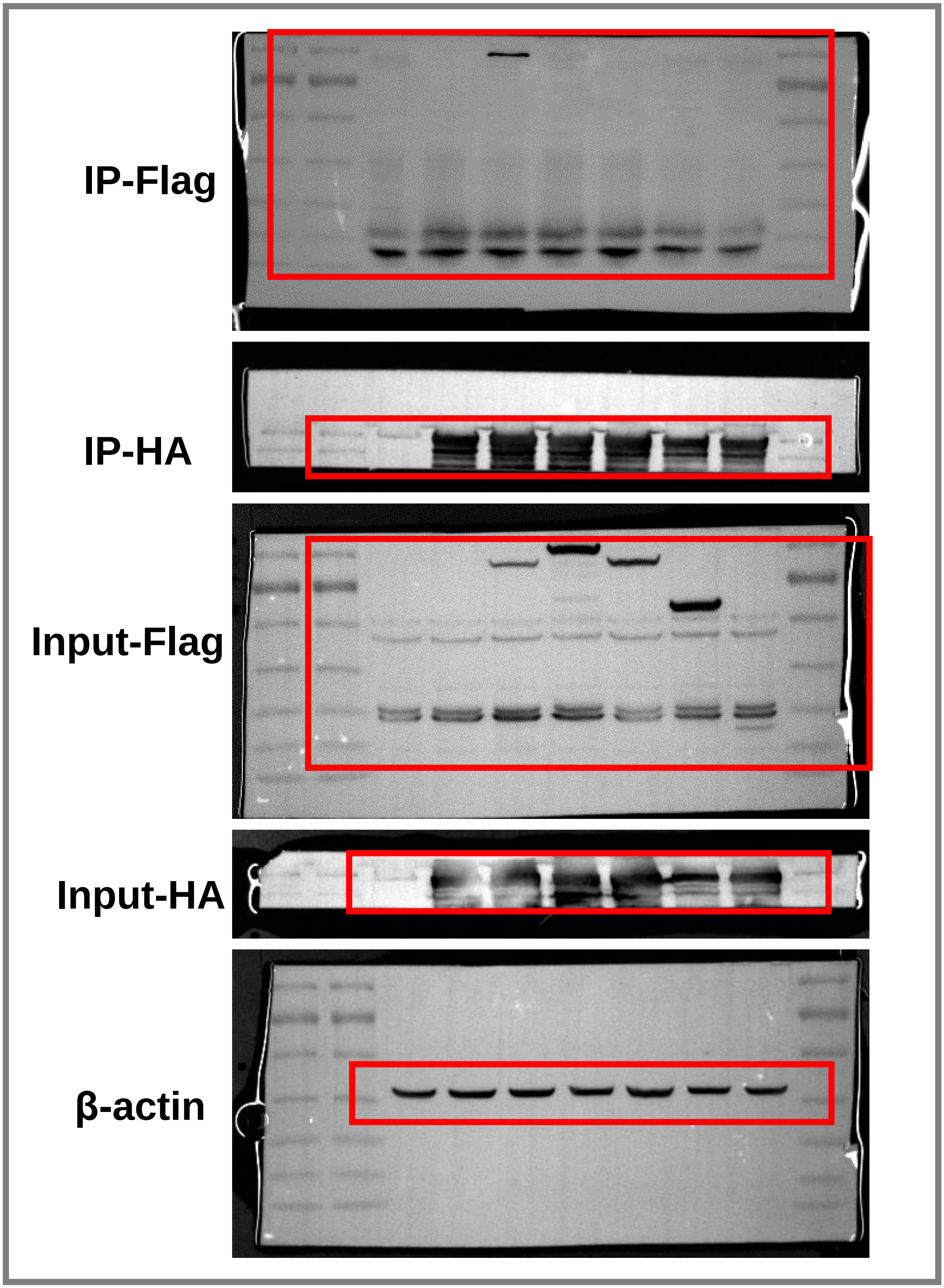

Figure 6C

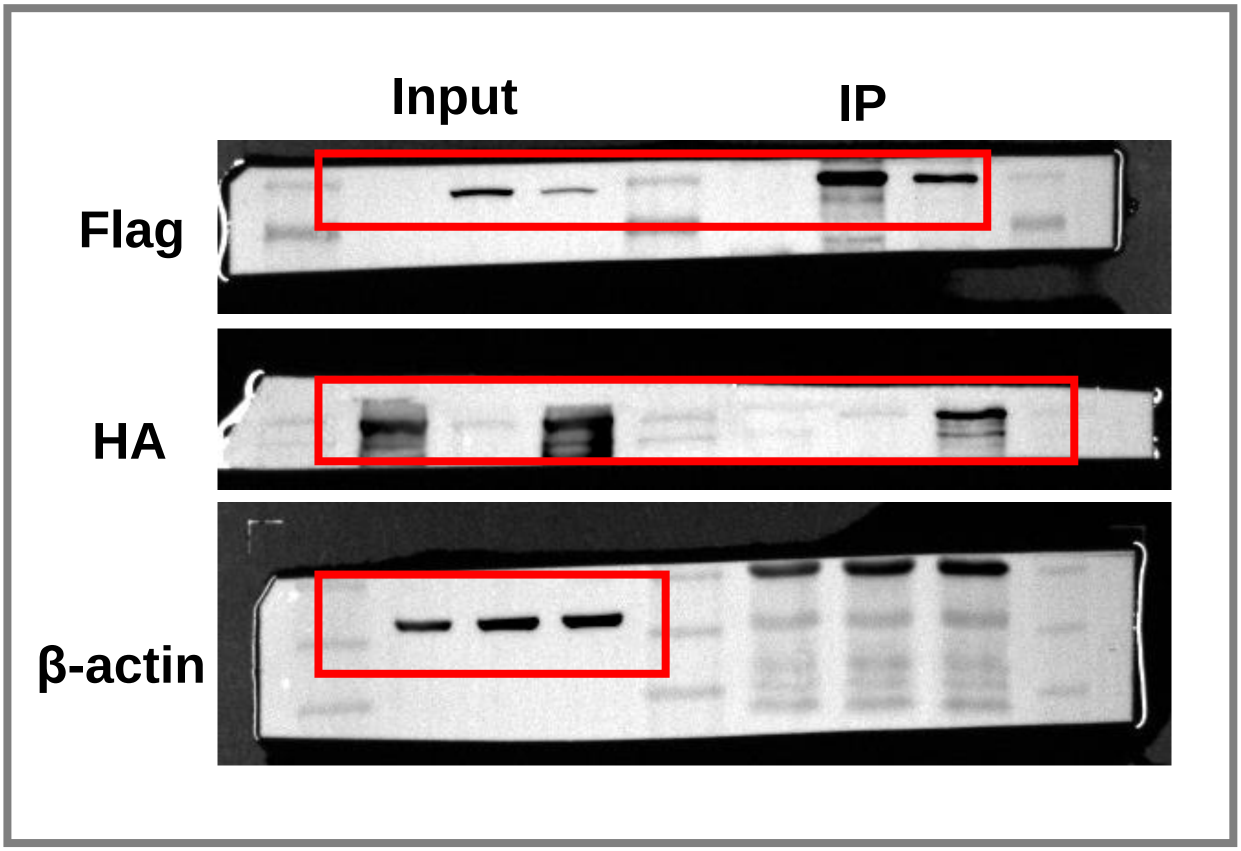

Figure 6D

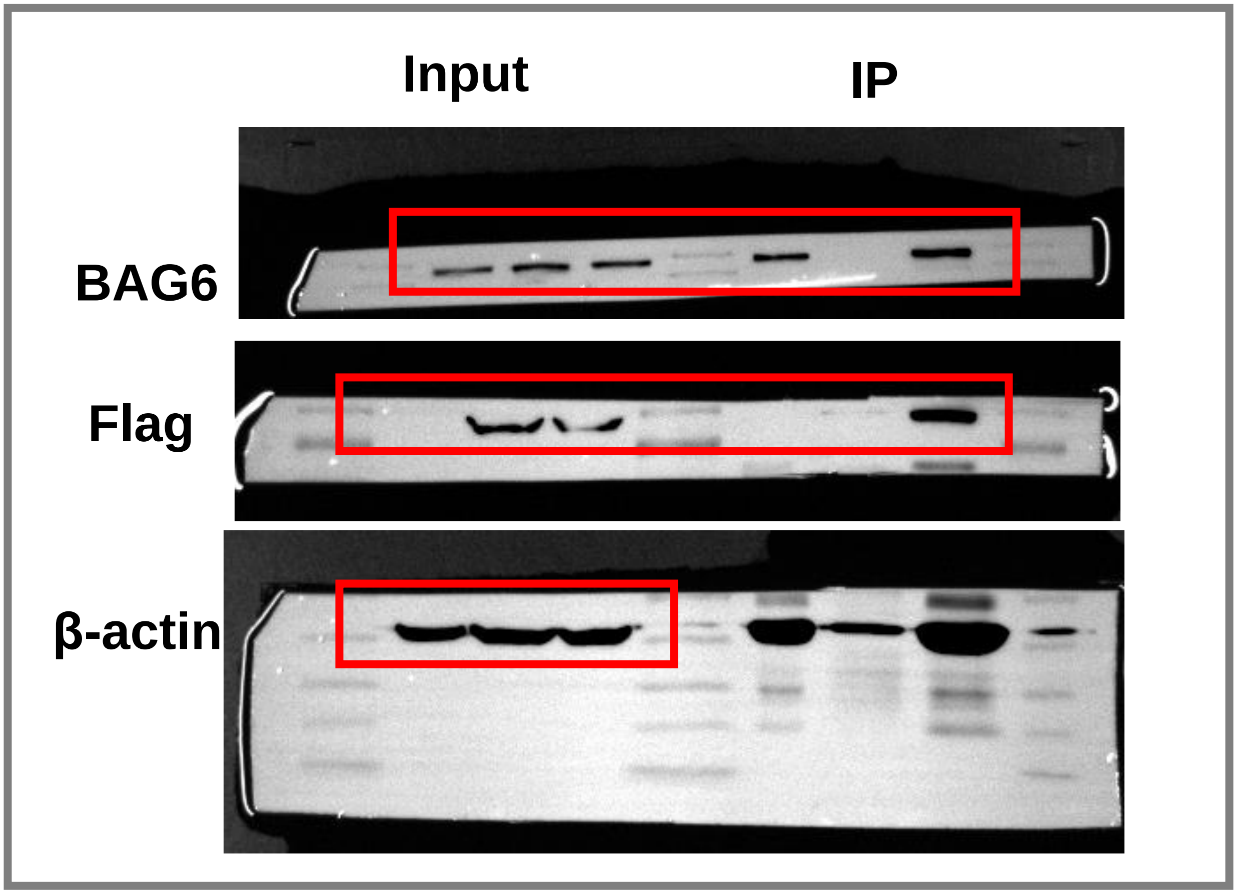

Figure 6F

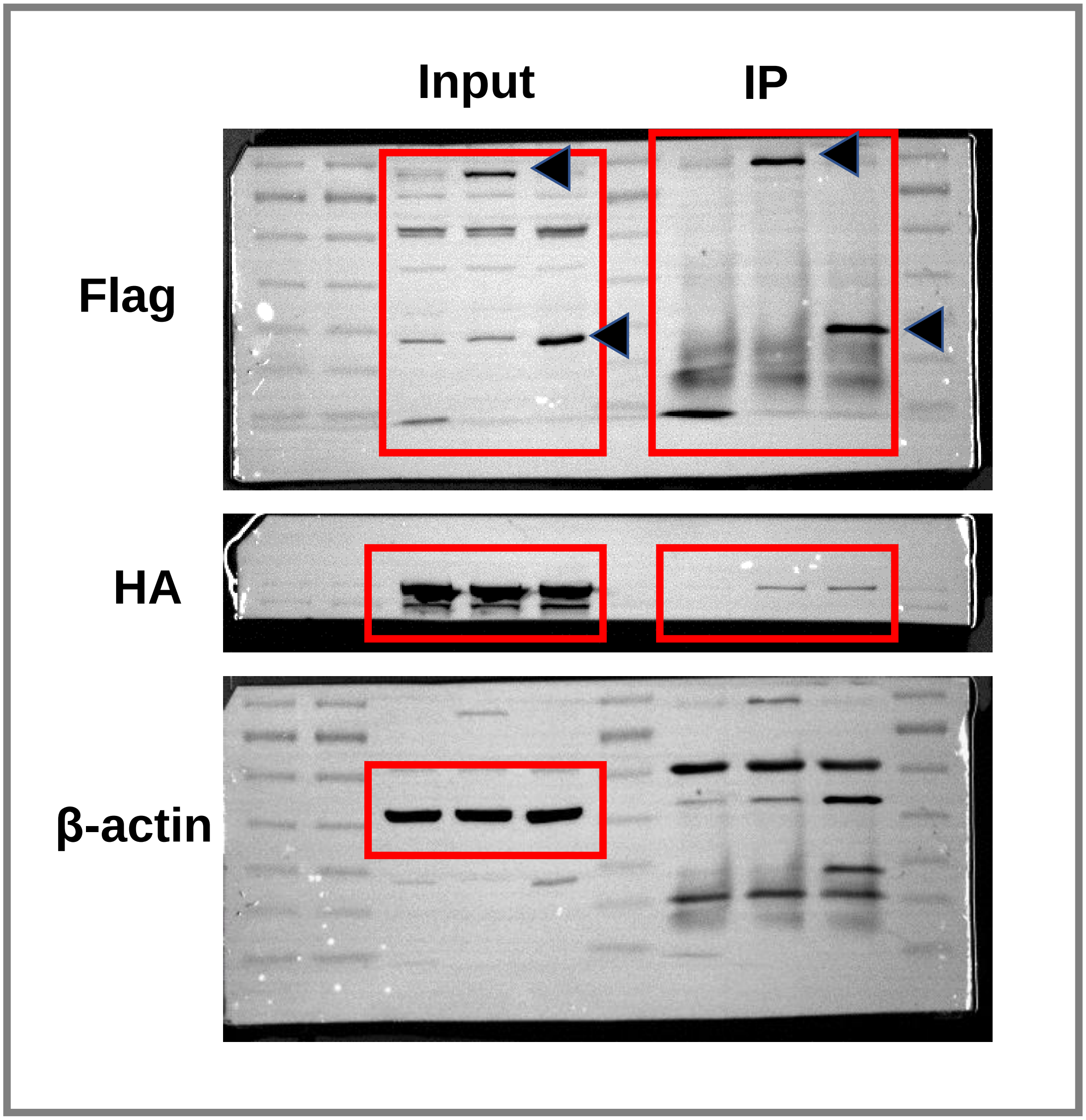

Figure 6G

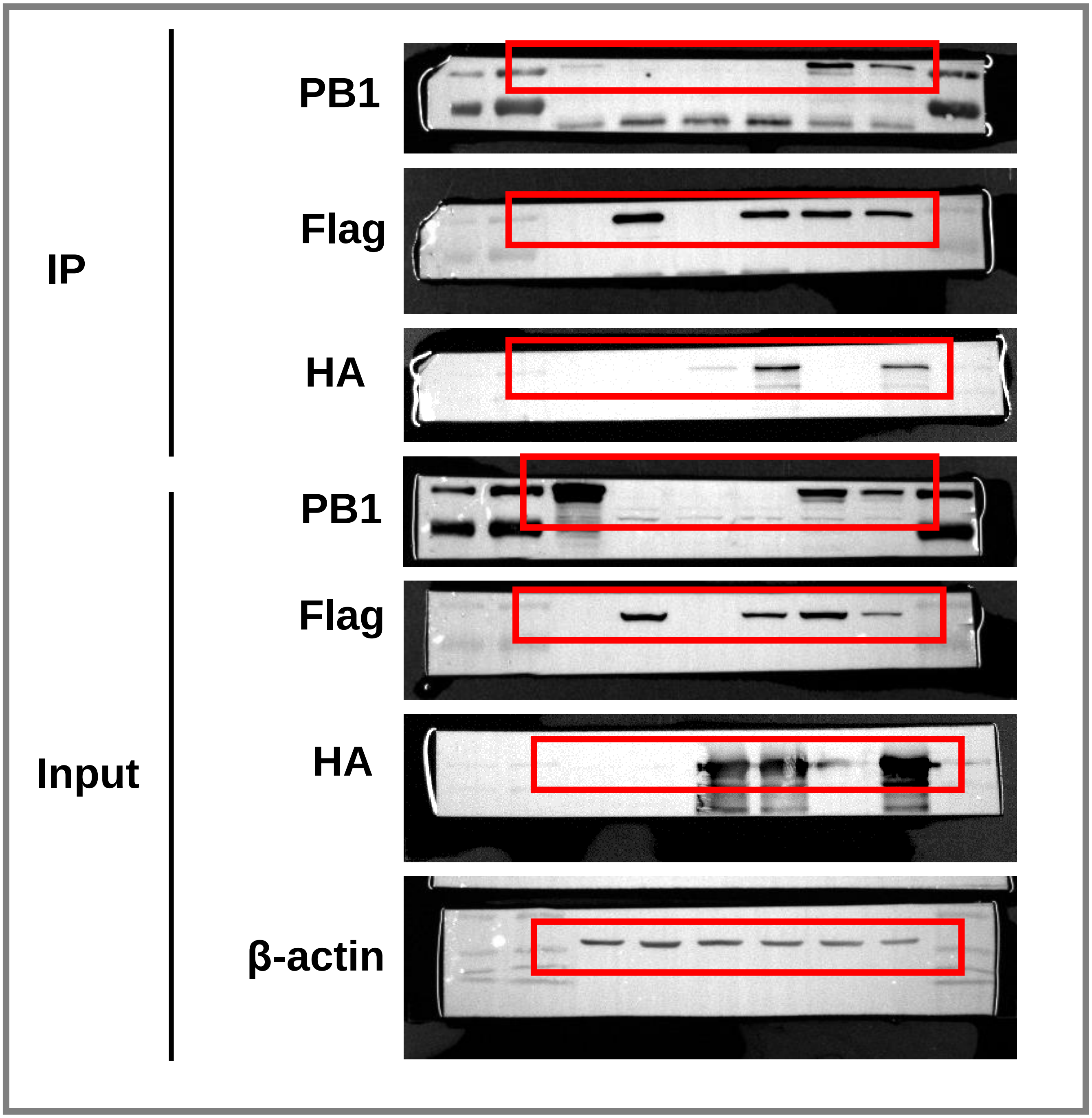

Figure 6H

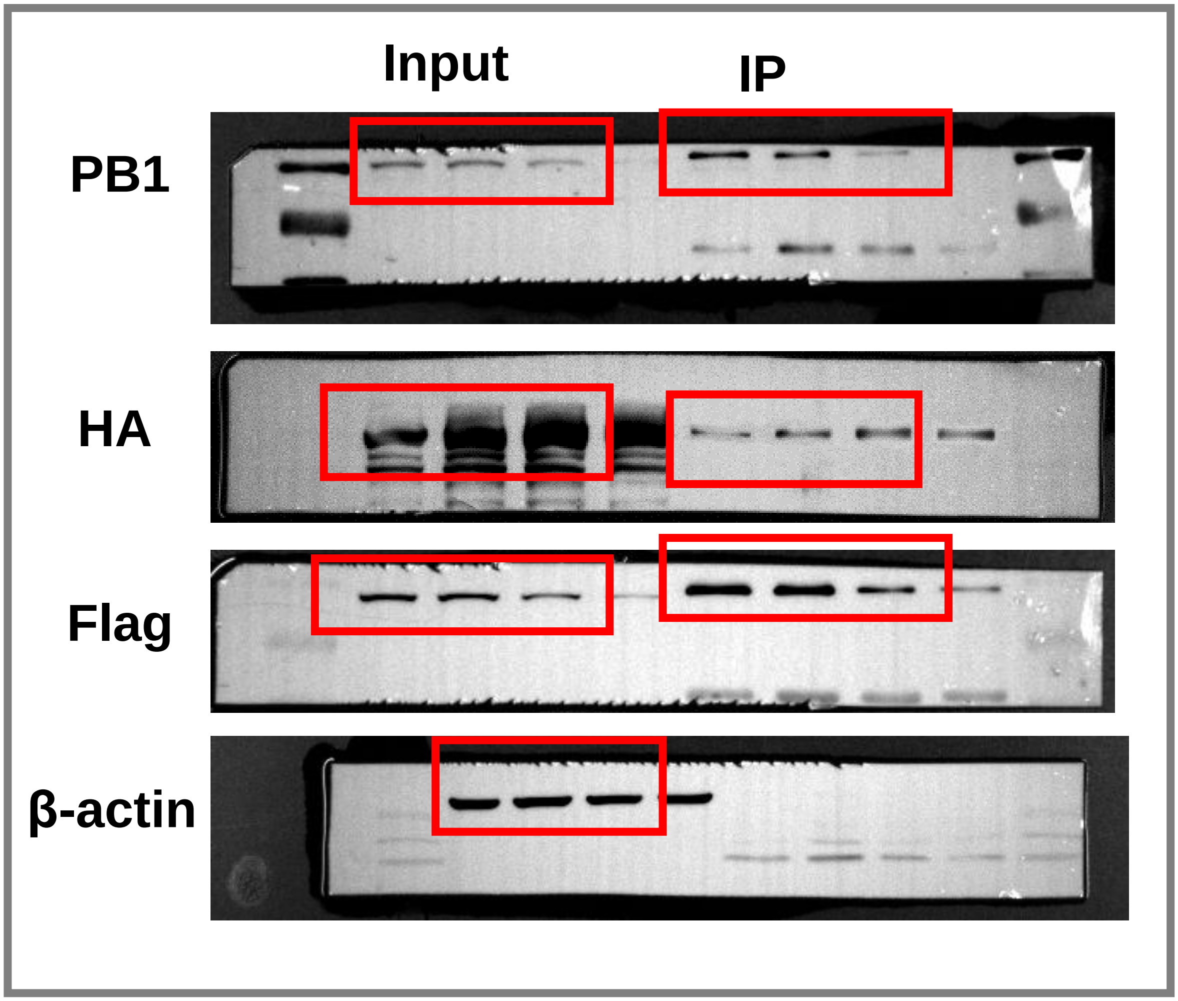

Figure 7A

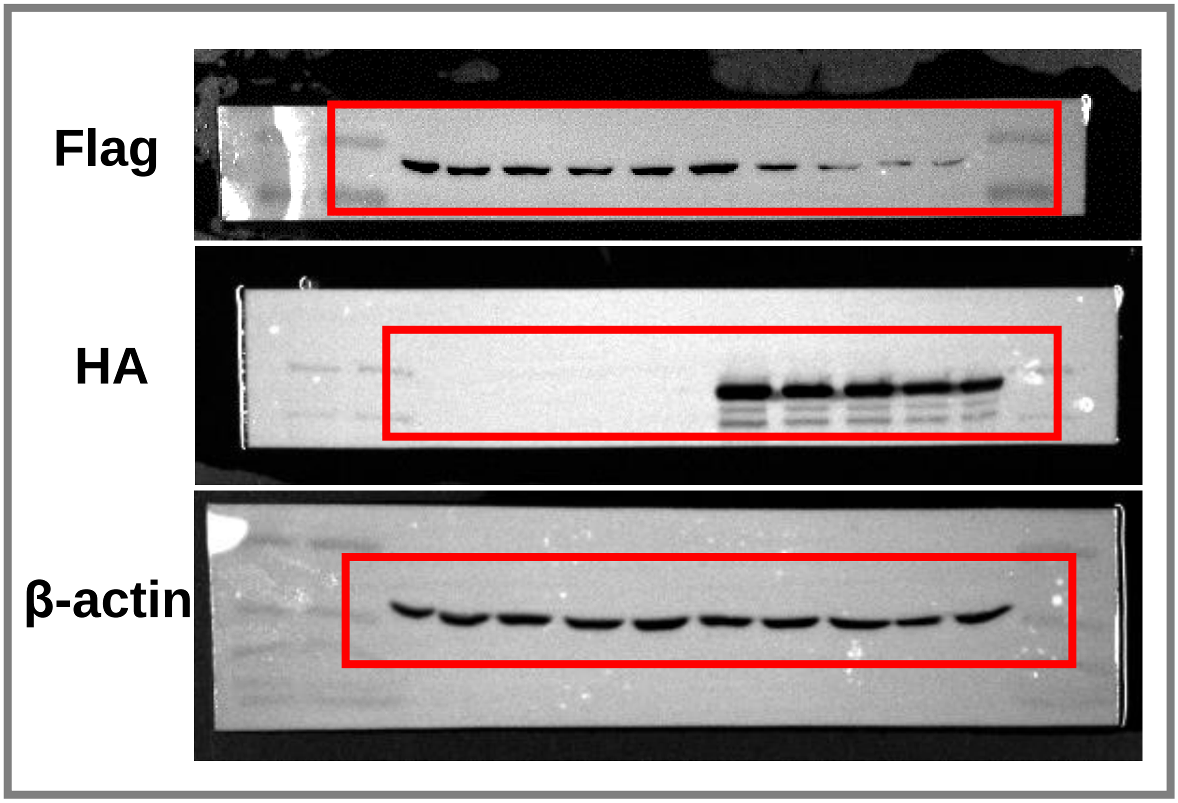

Figure 7B

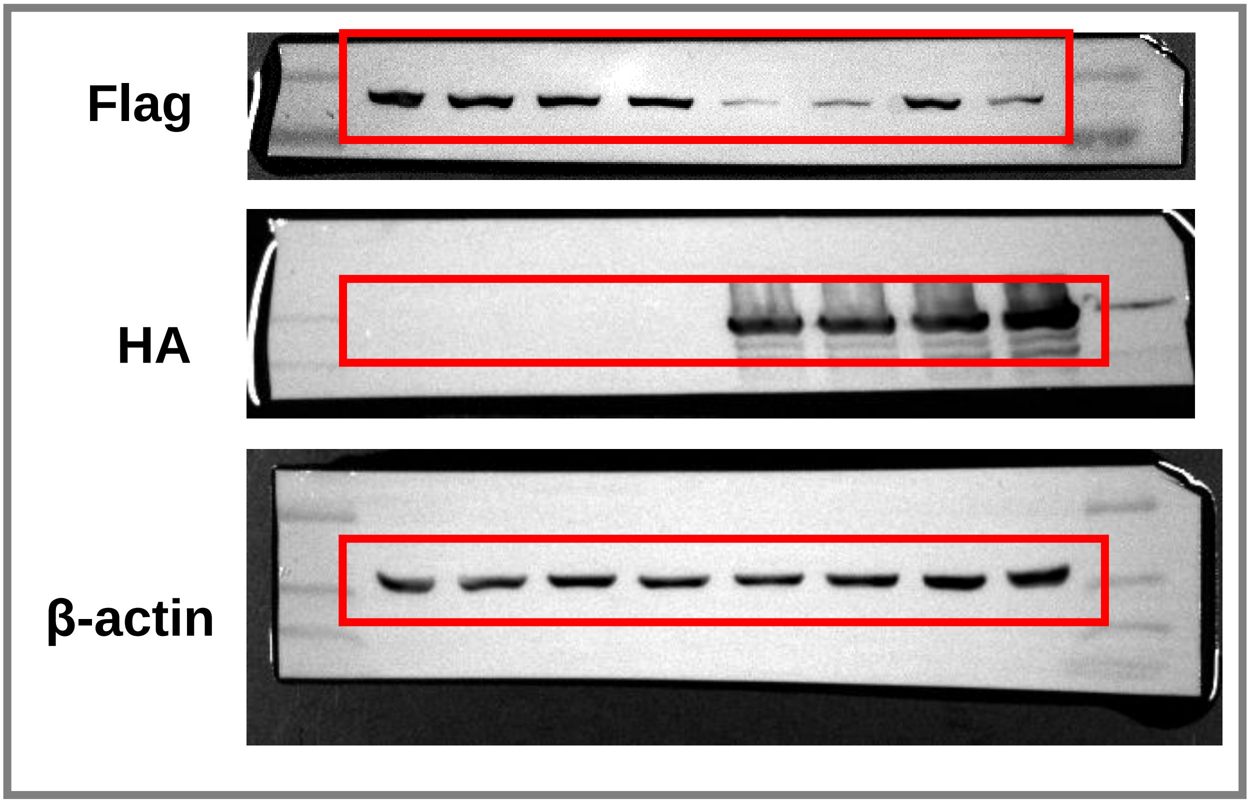

Figure 7C

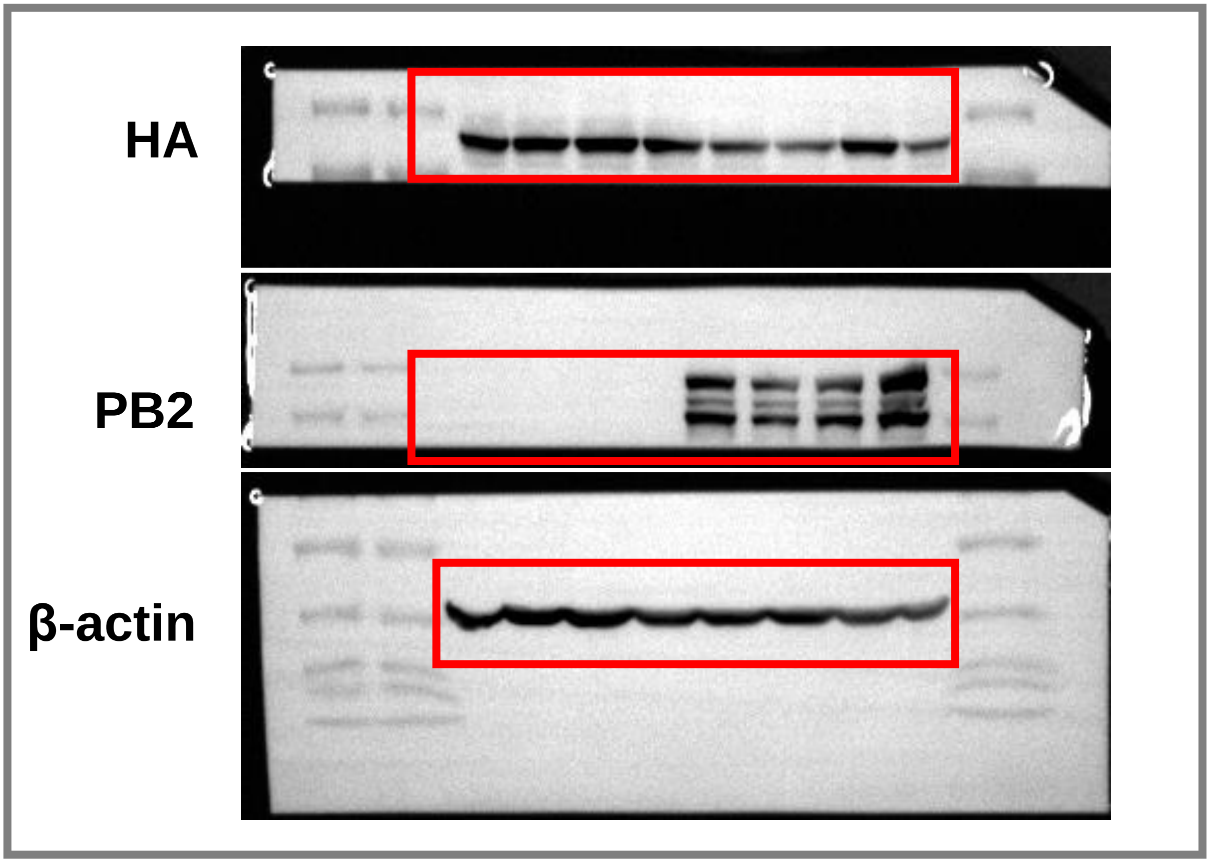

Figure 7D

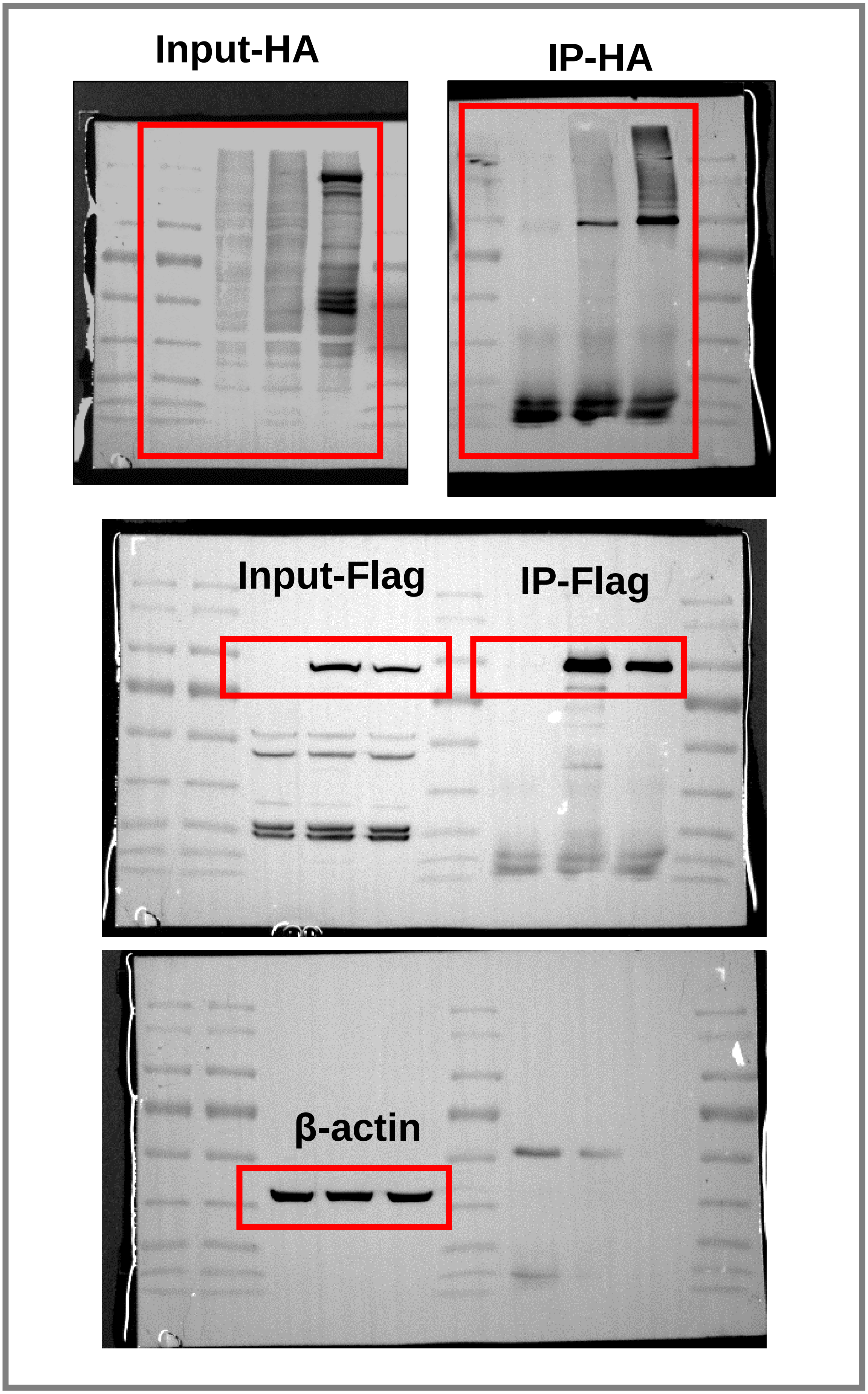

Figure 7E

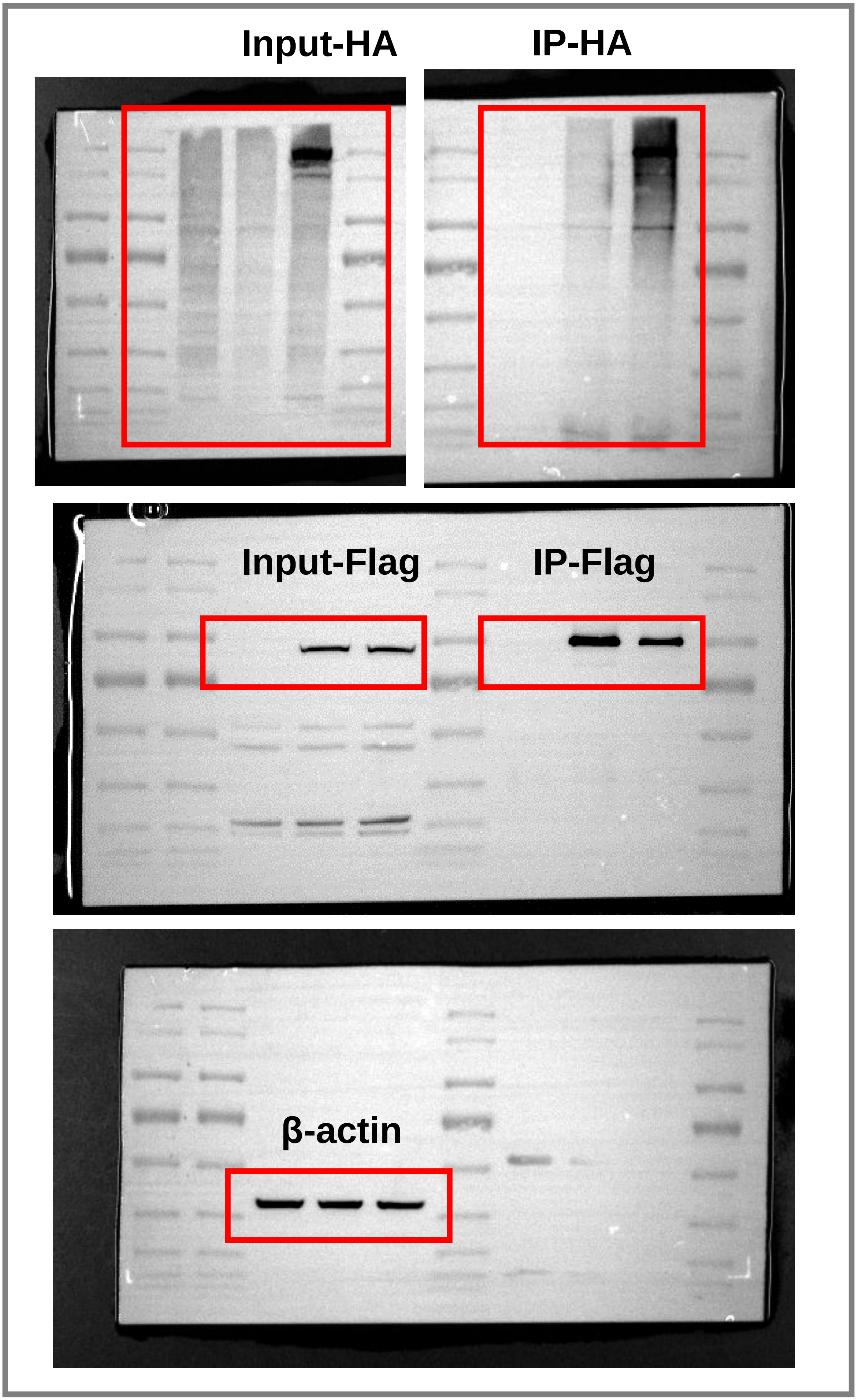

Figure 7E

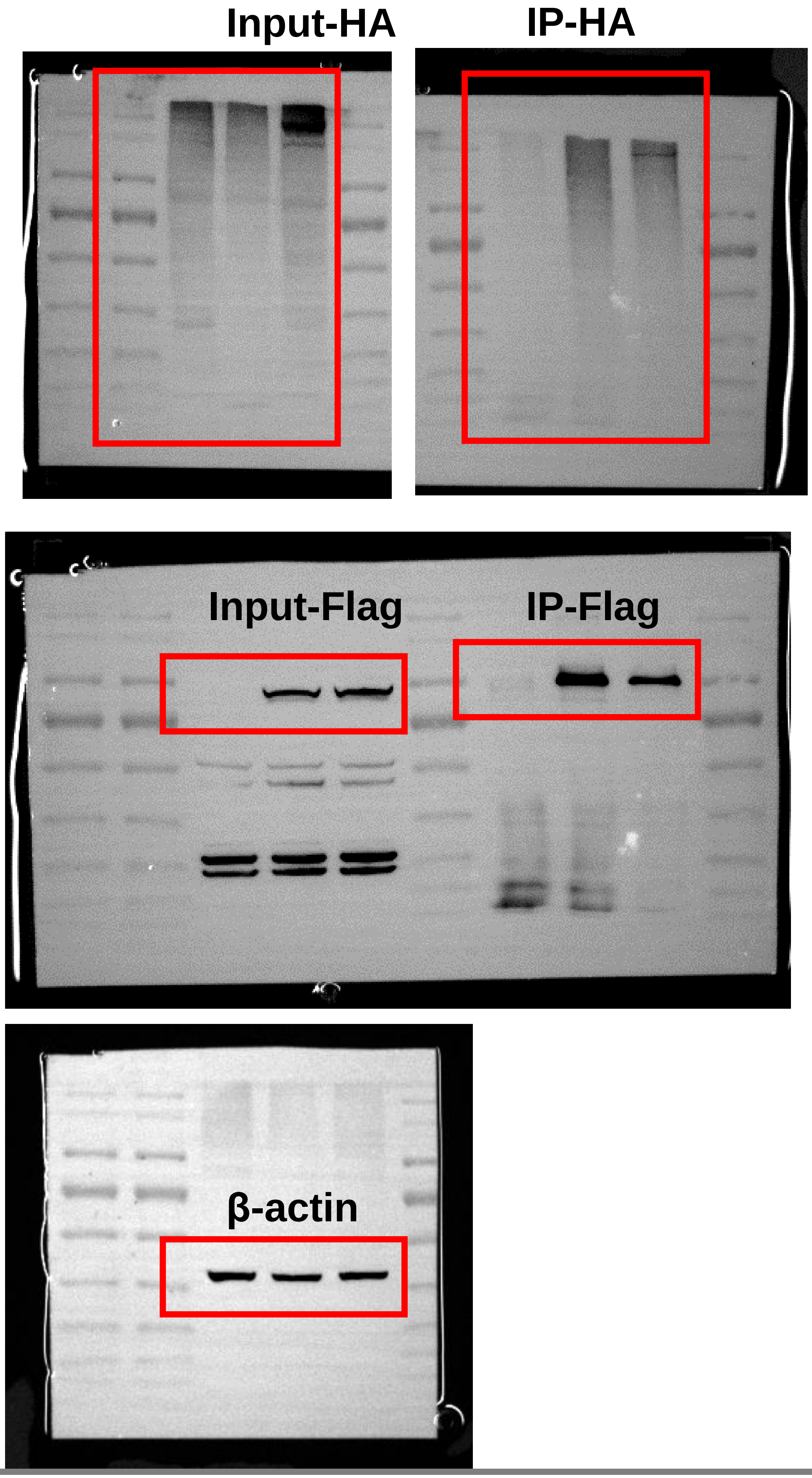

Figure 7F

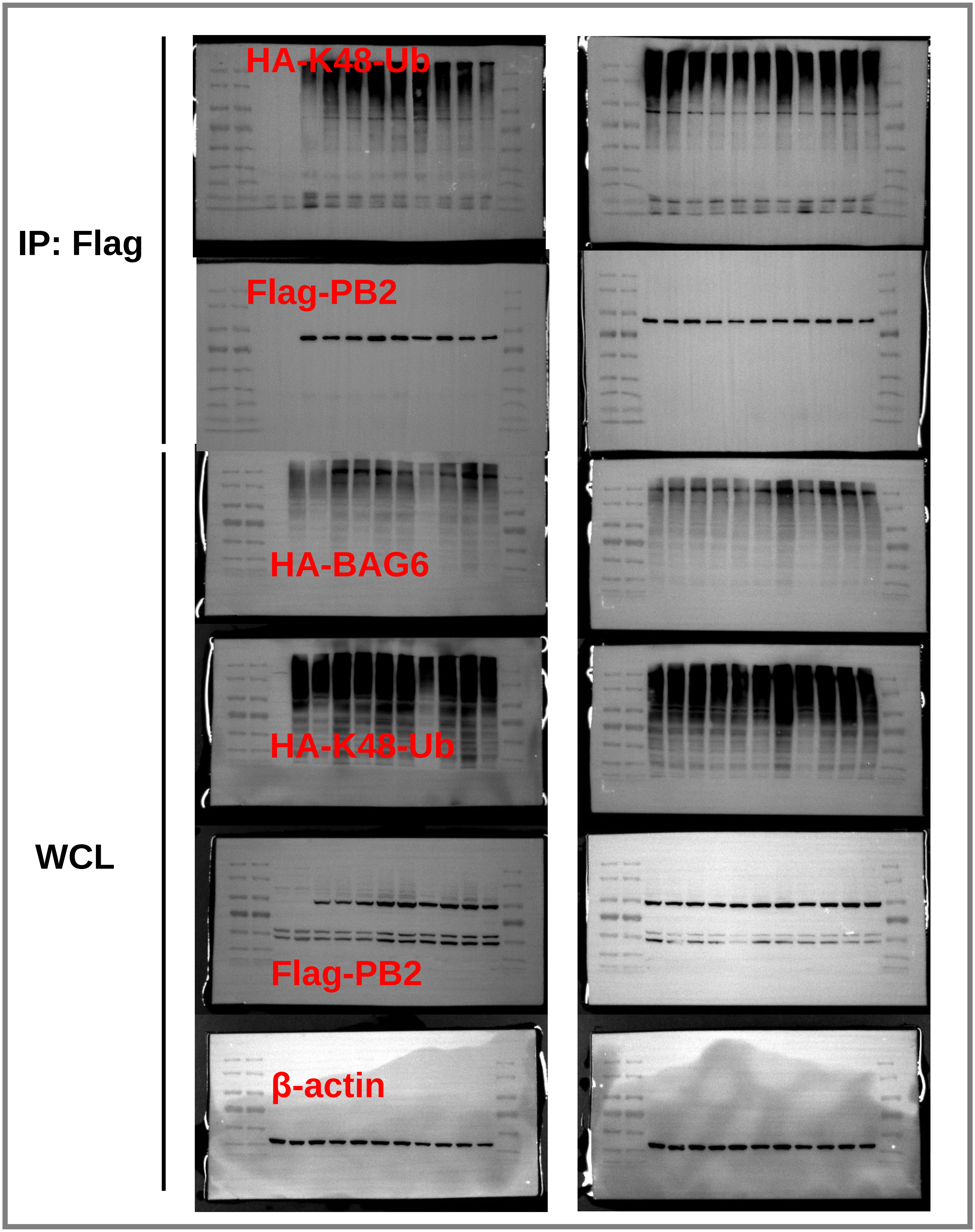

Figure 7G

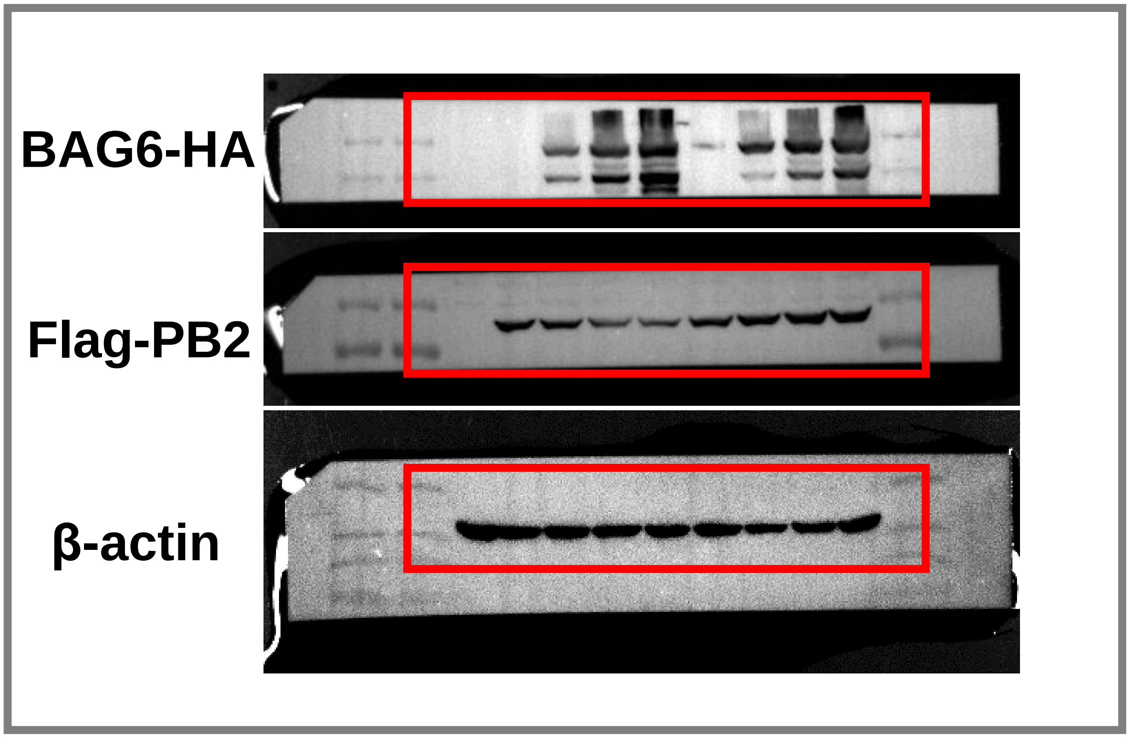

Figure 8B

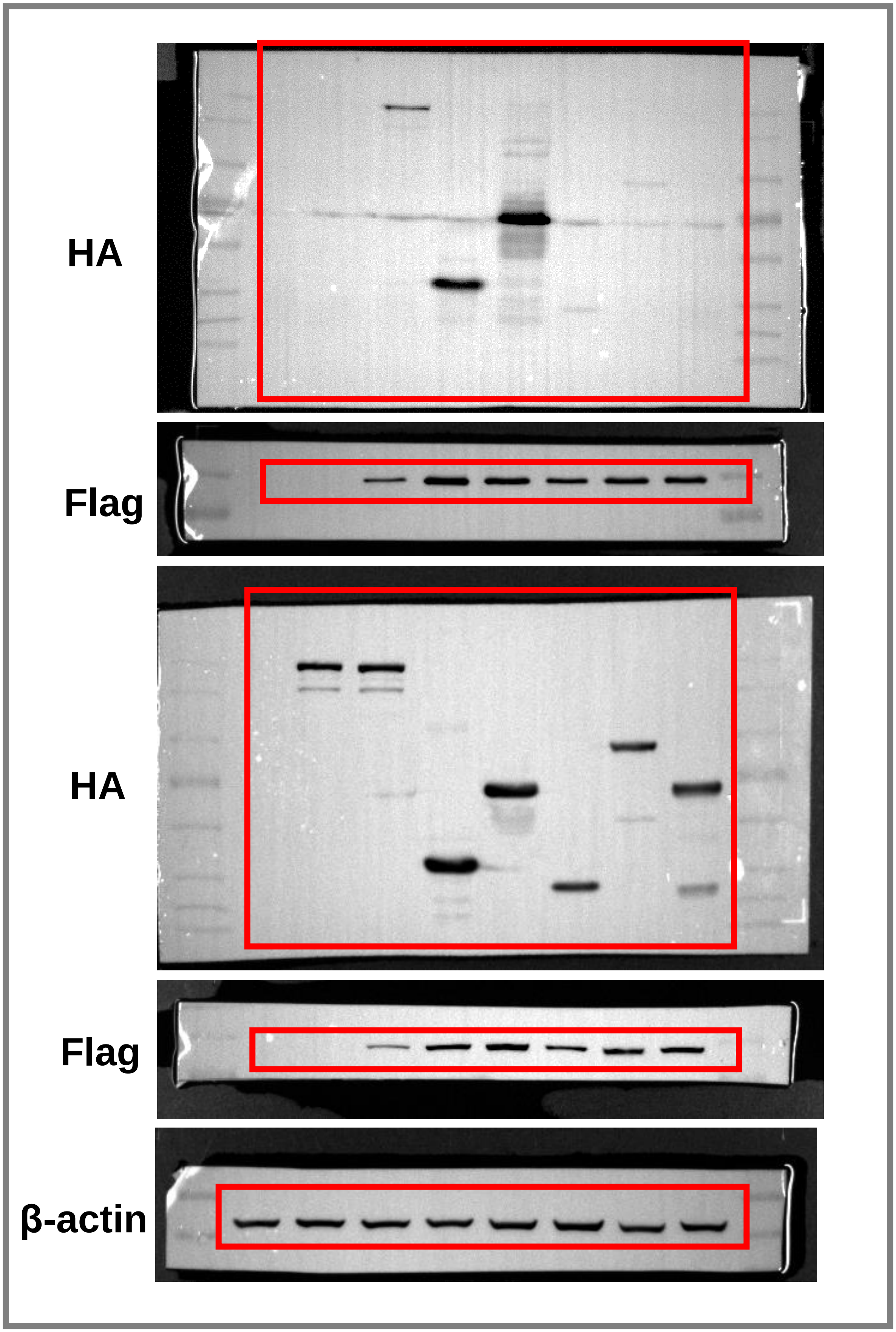

Figure 8C

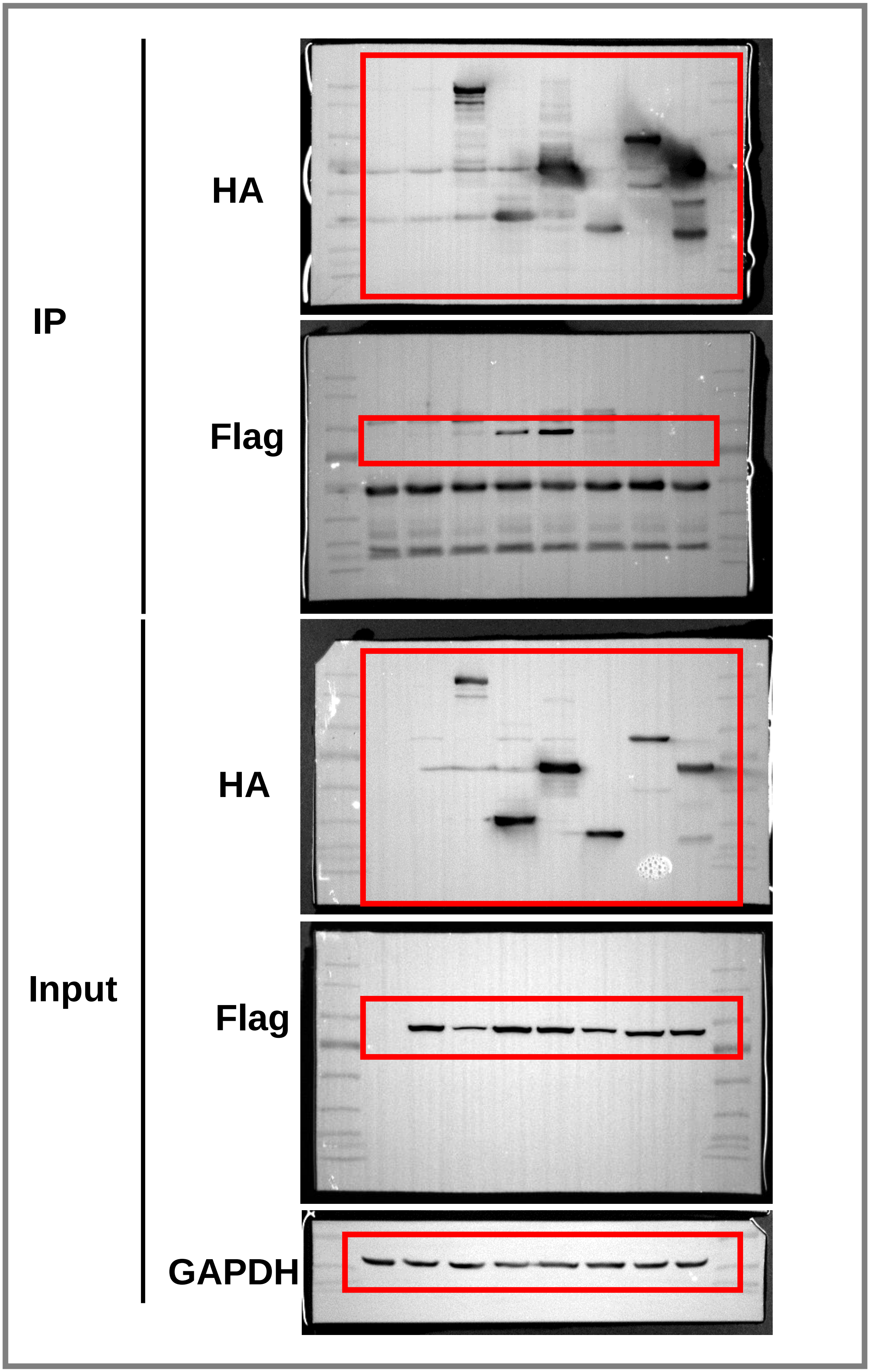

Figure 8E

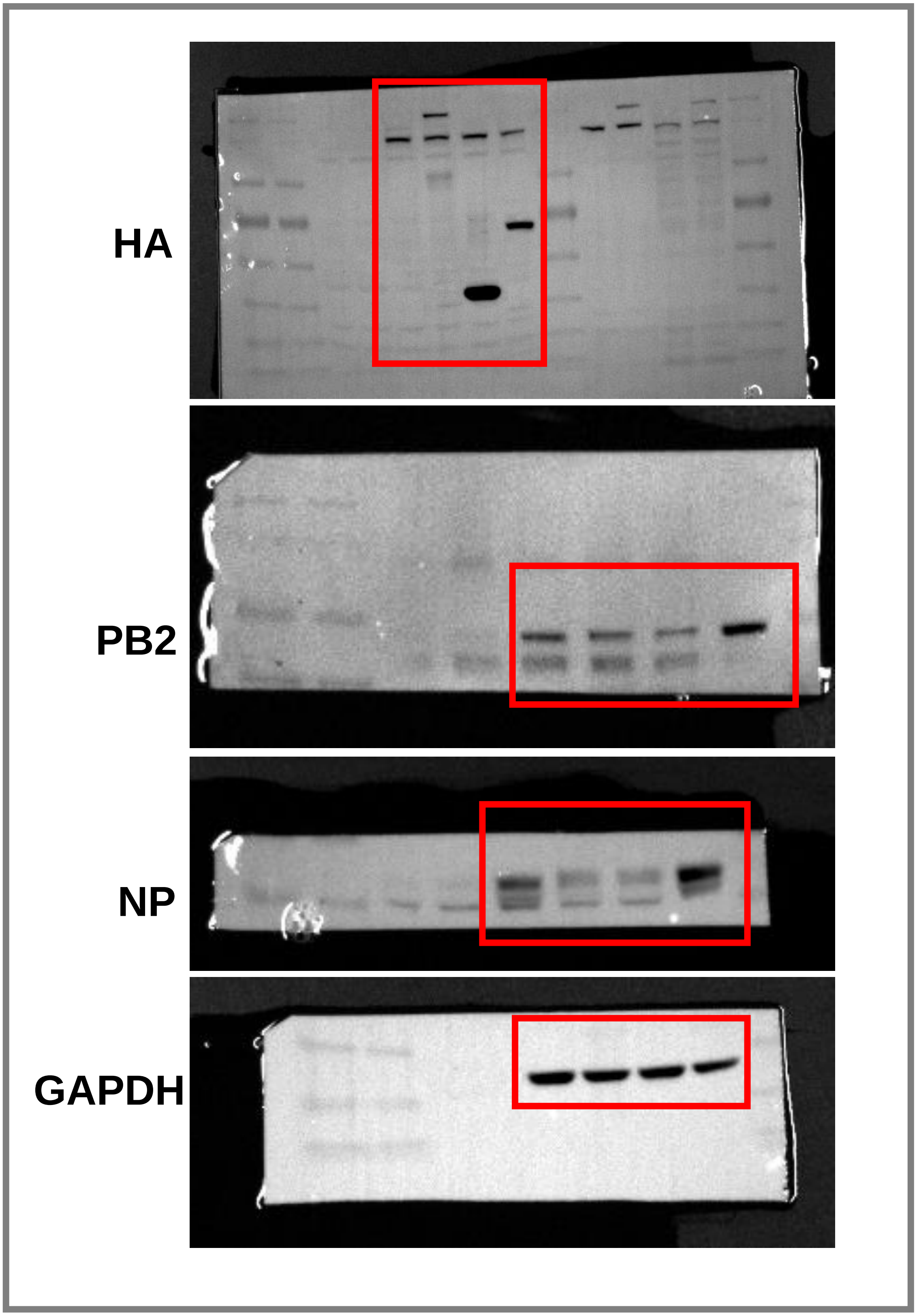

Figure 8H

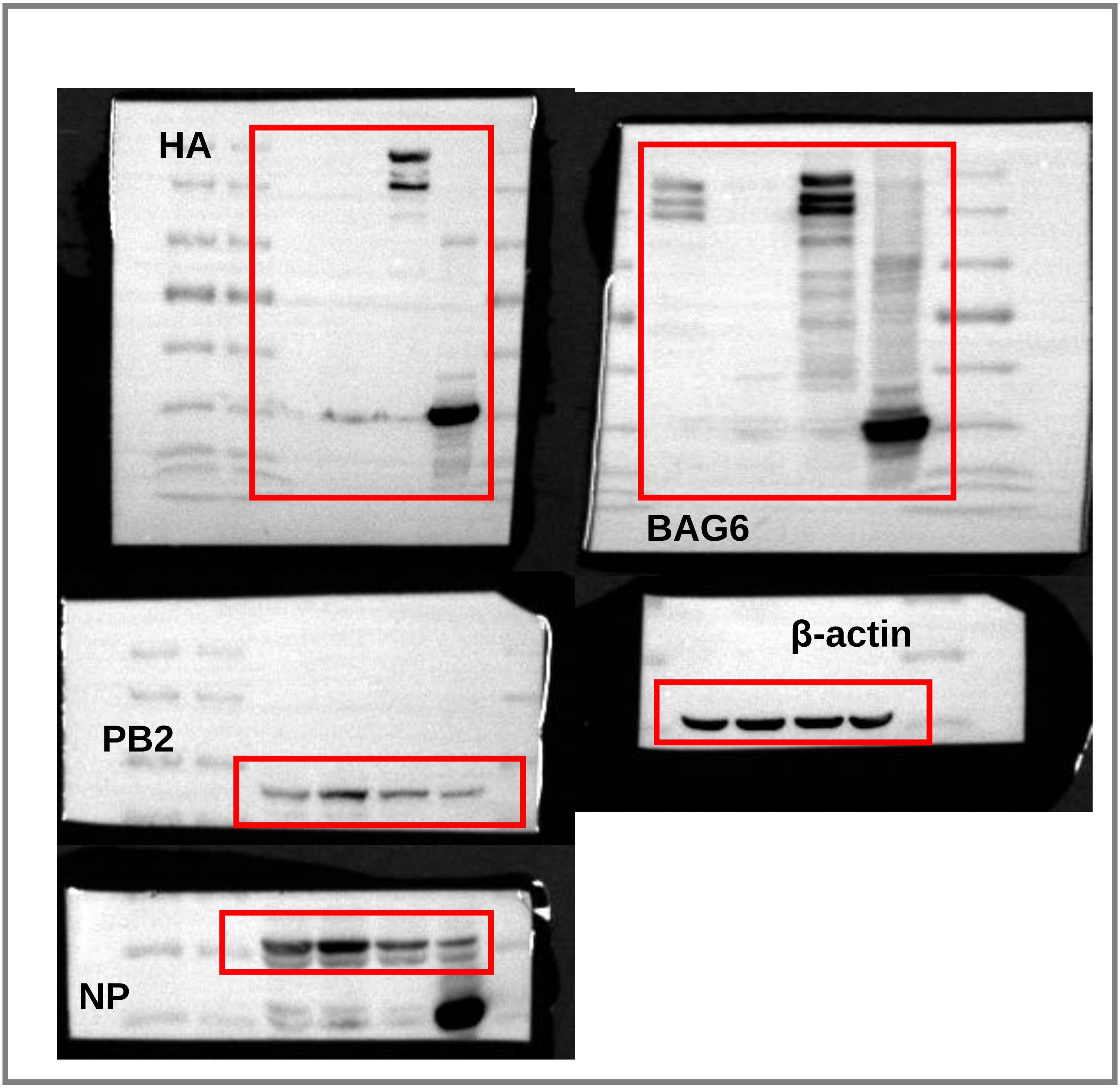

Figure 9C

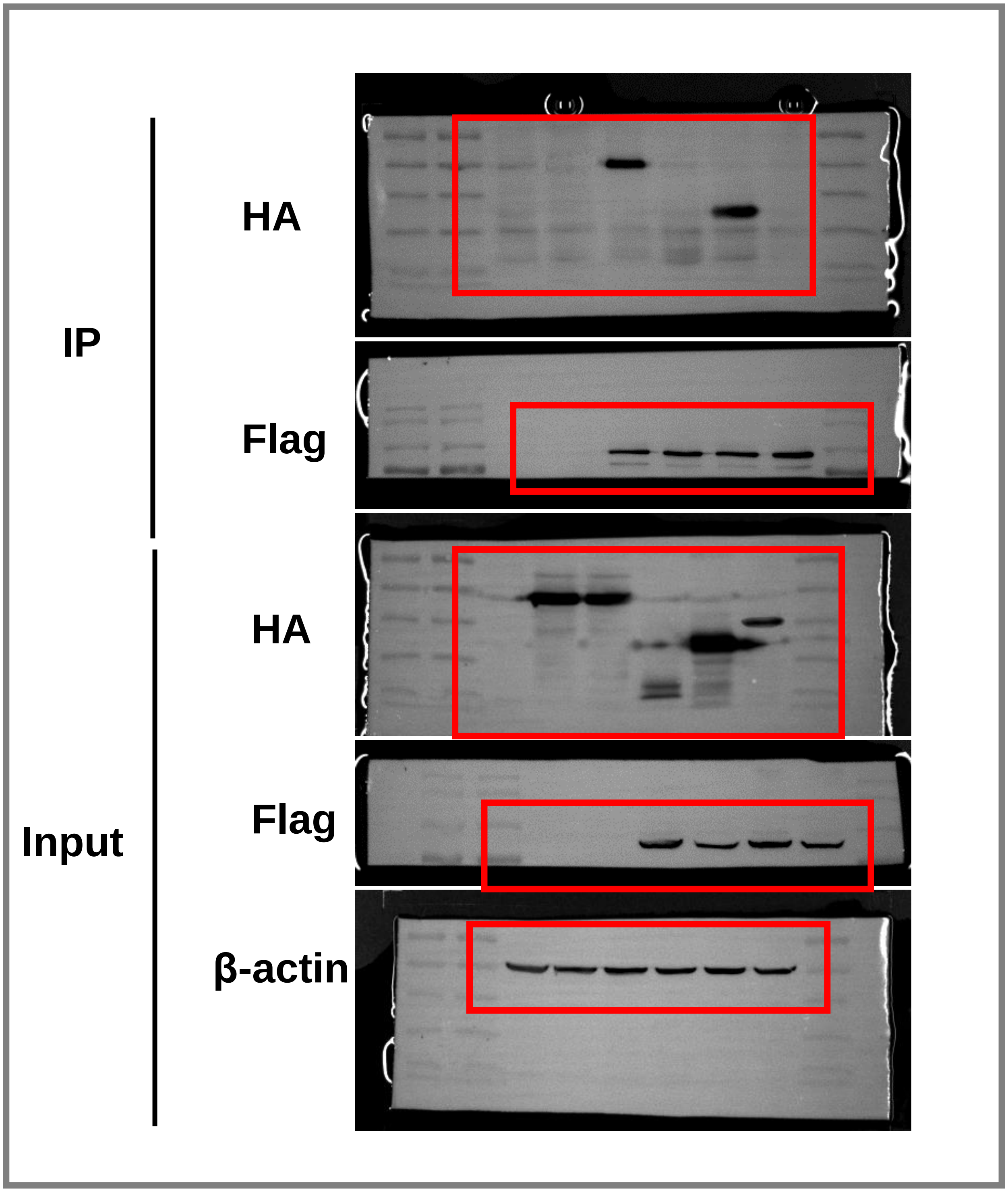

Figure 9D

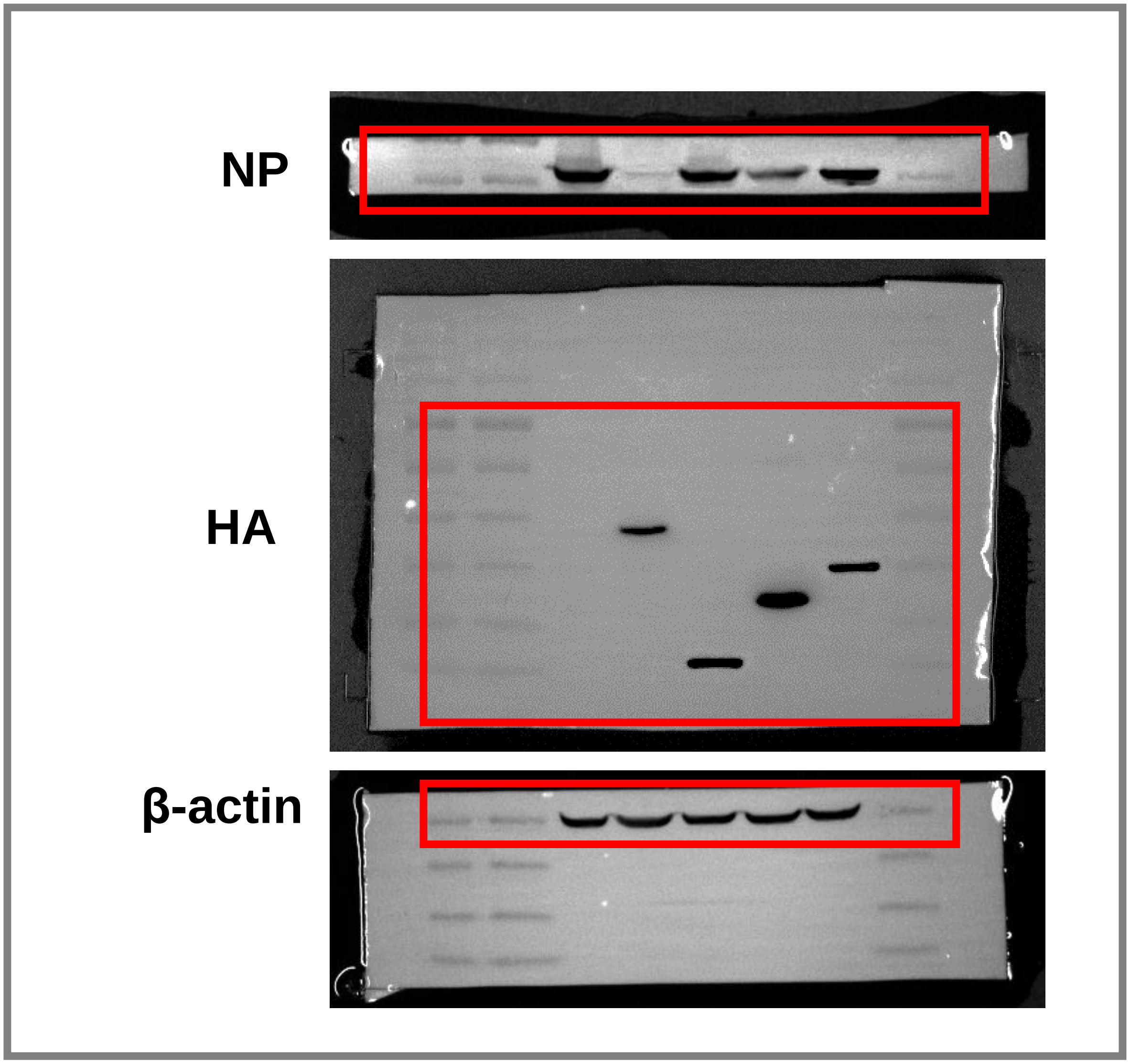

Figure S1A

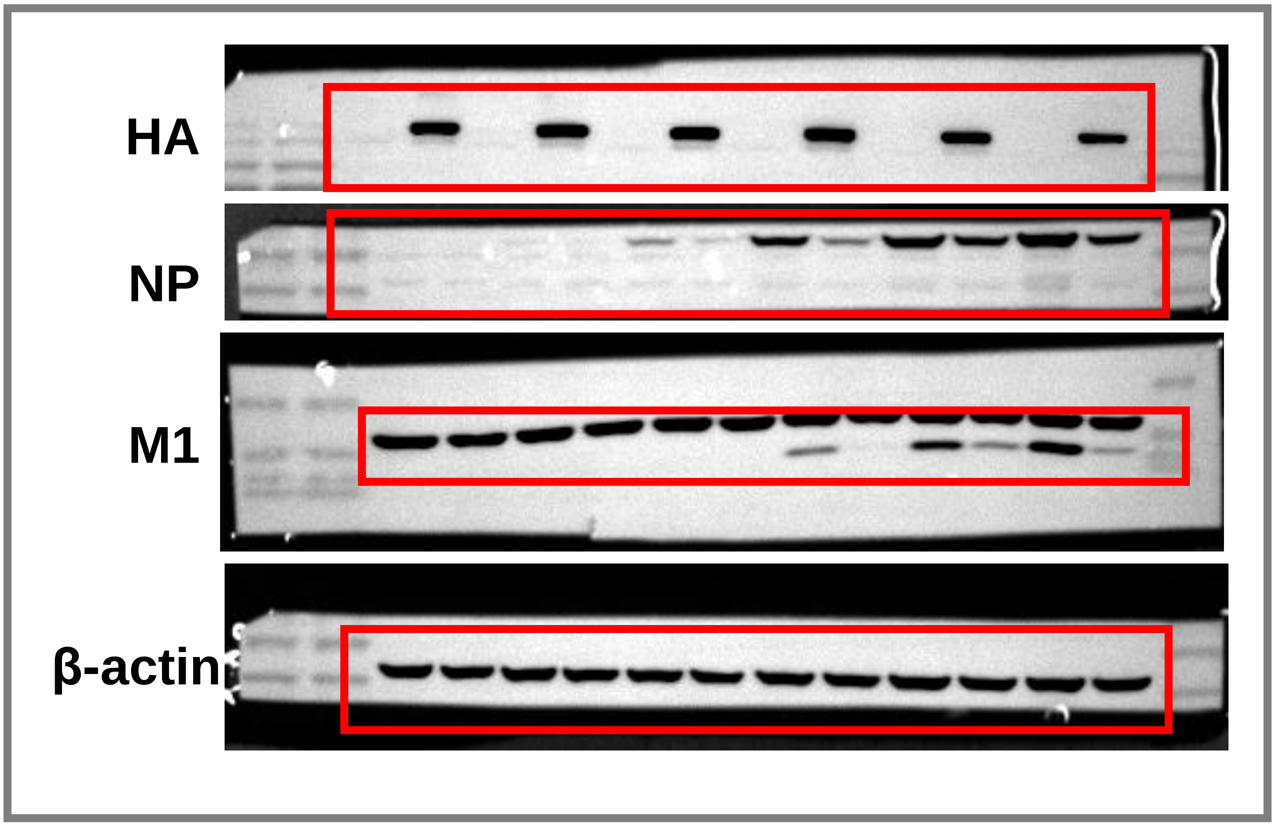

Figure S1C

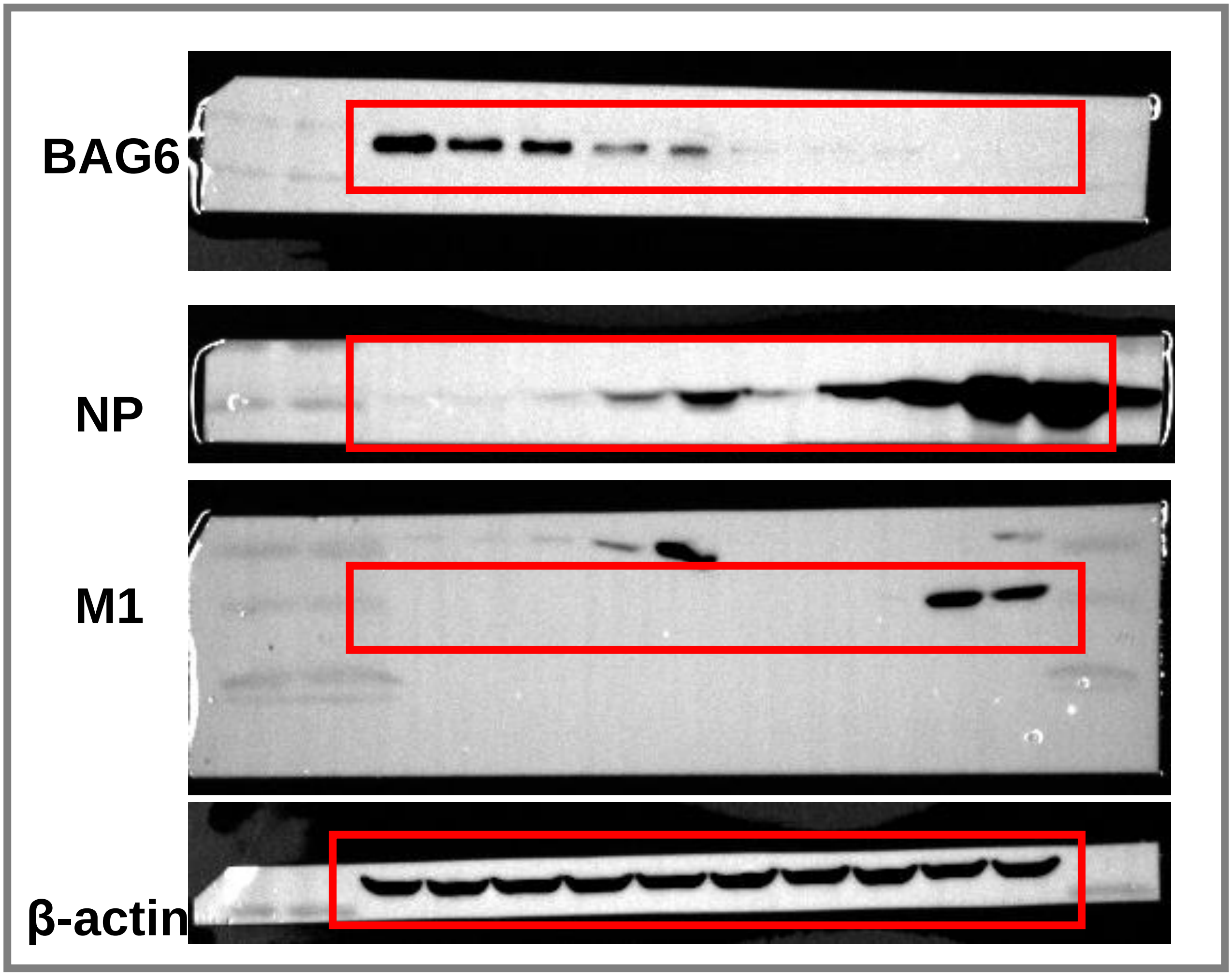

Figure S3A

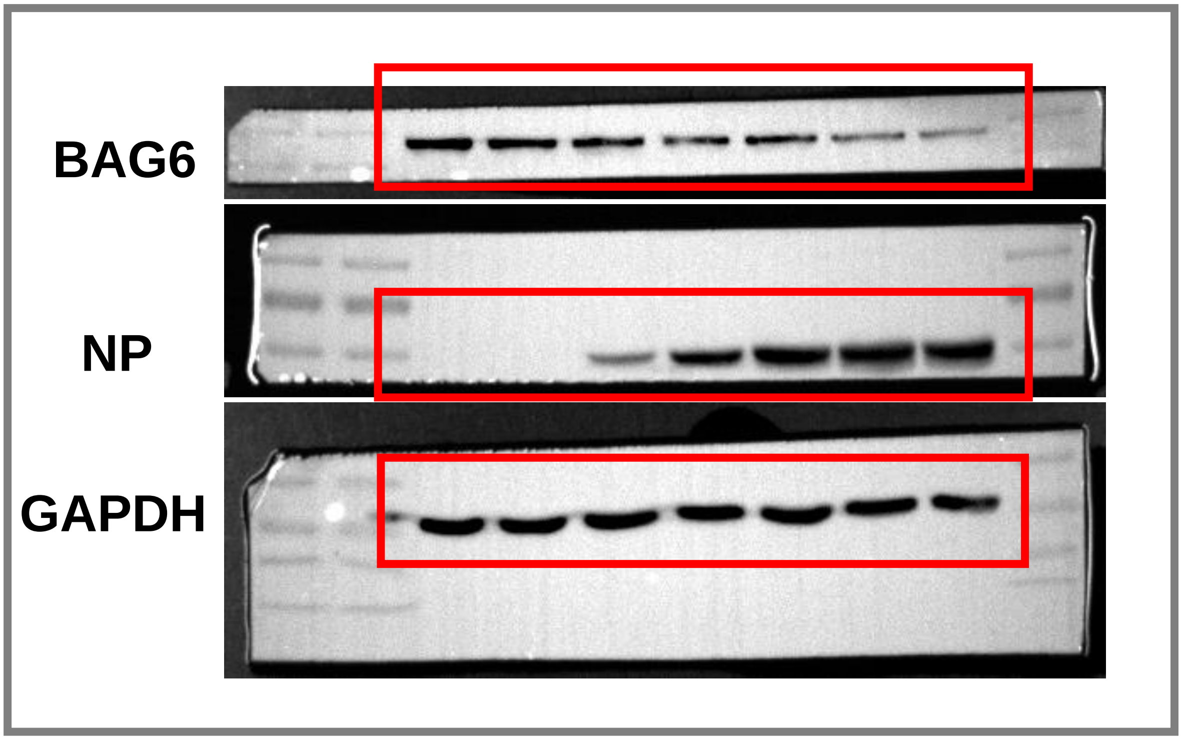

Figure S3B

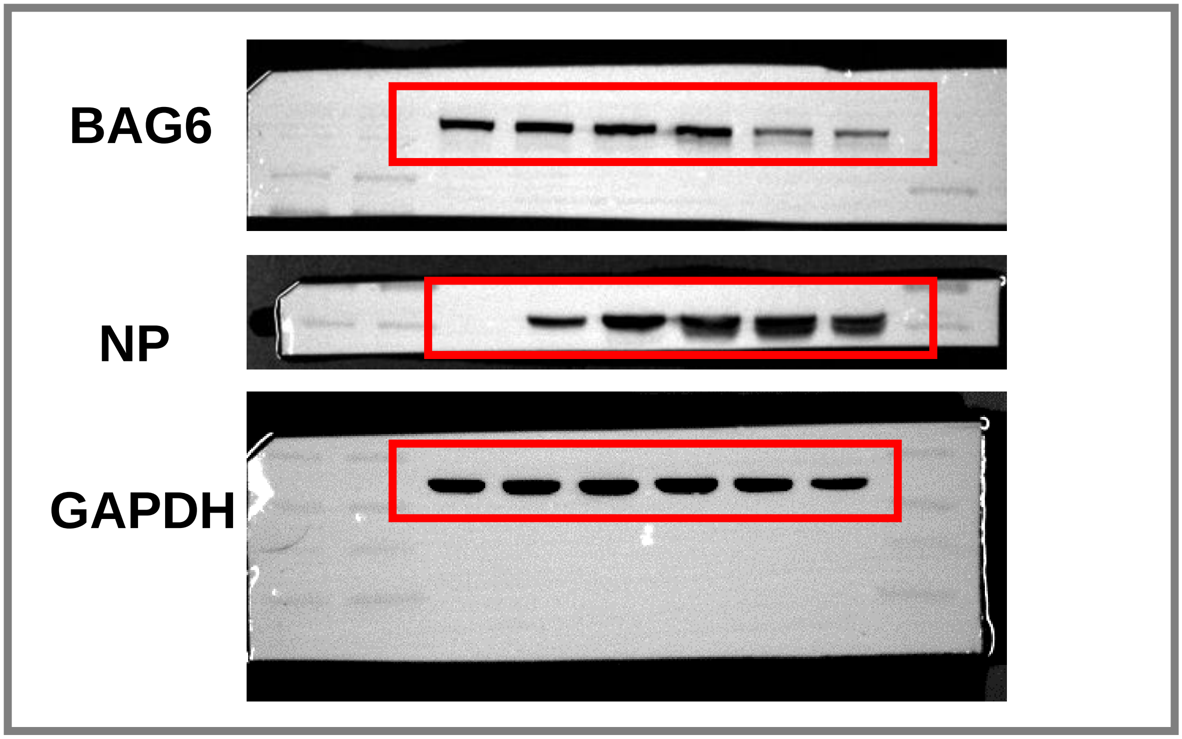

Figure S3C

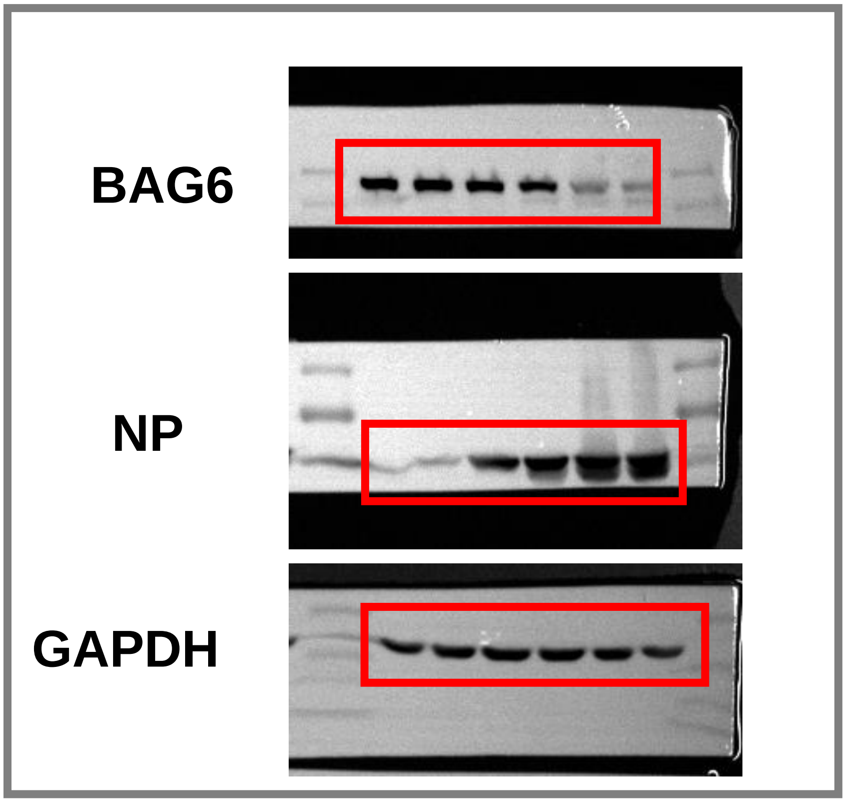

Figure S3D

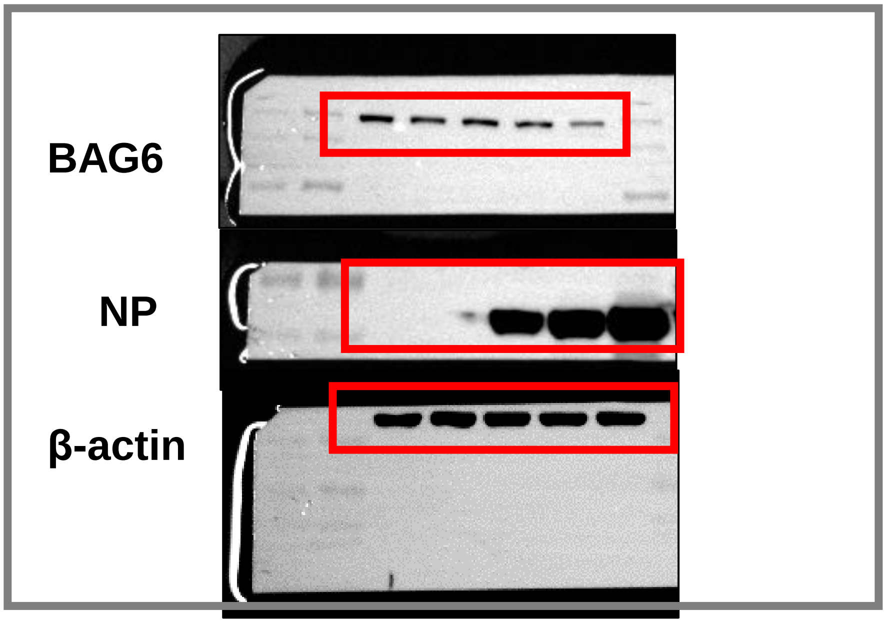

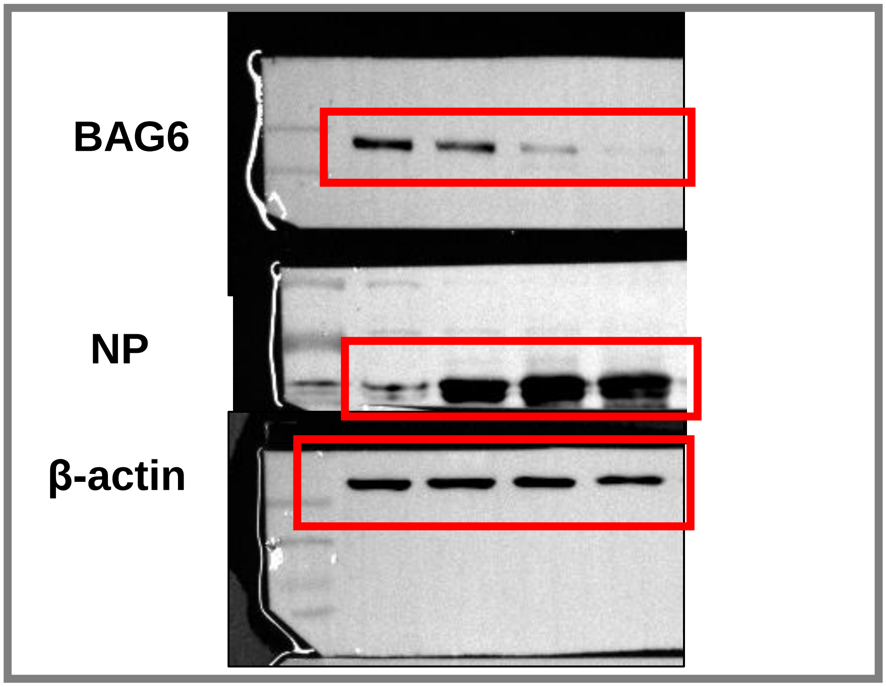

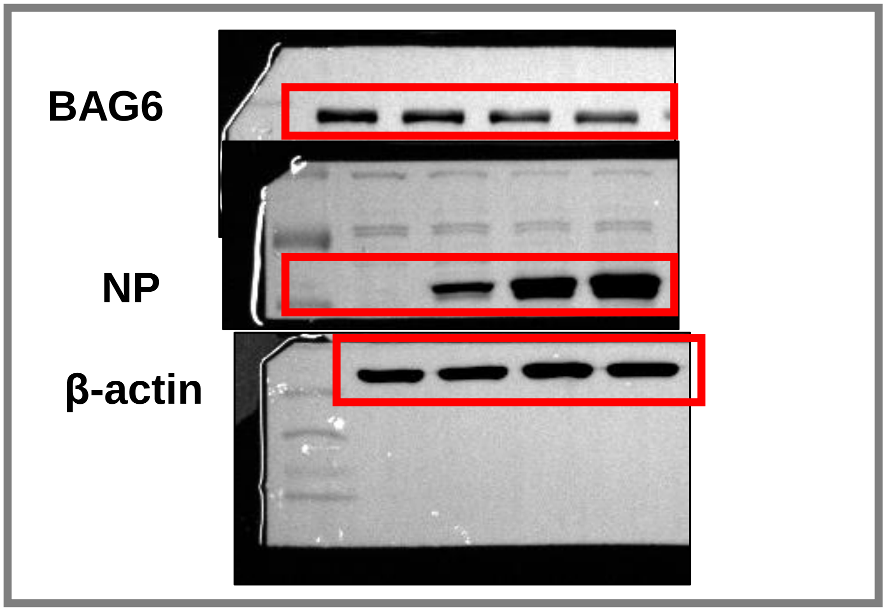

Figure S4A

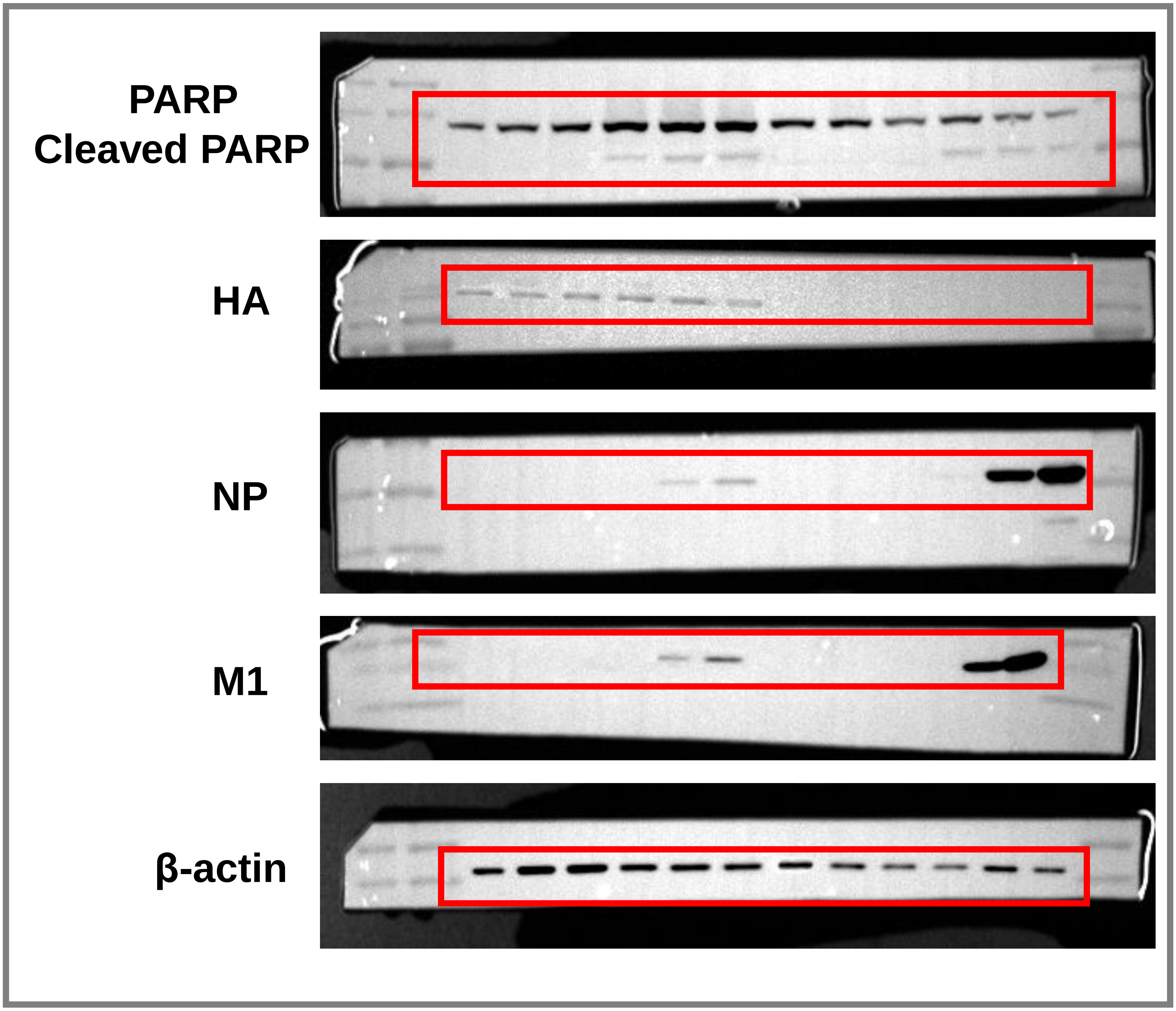

Figure 6E

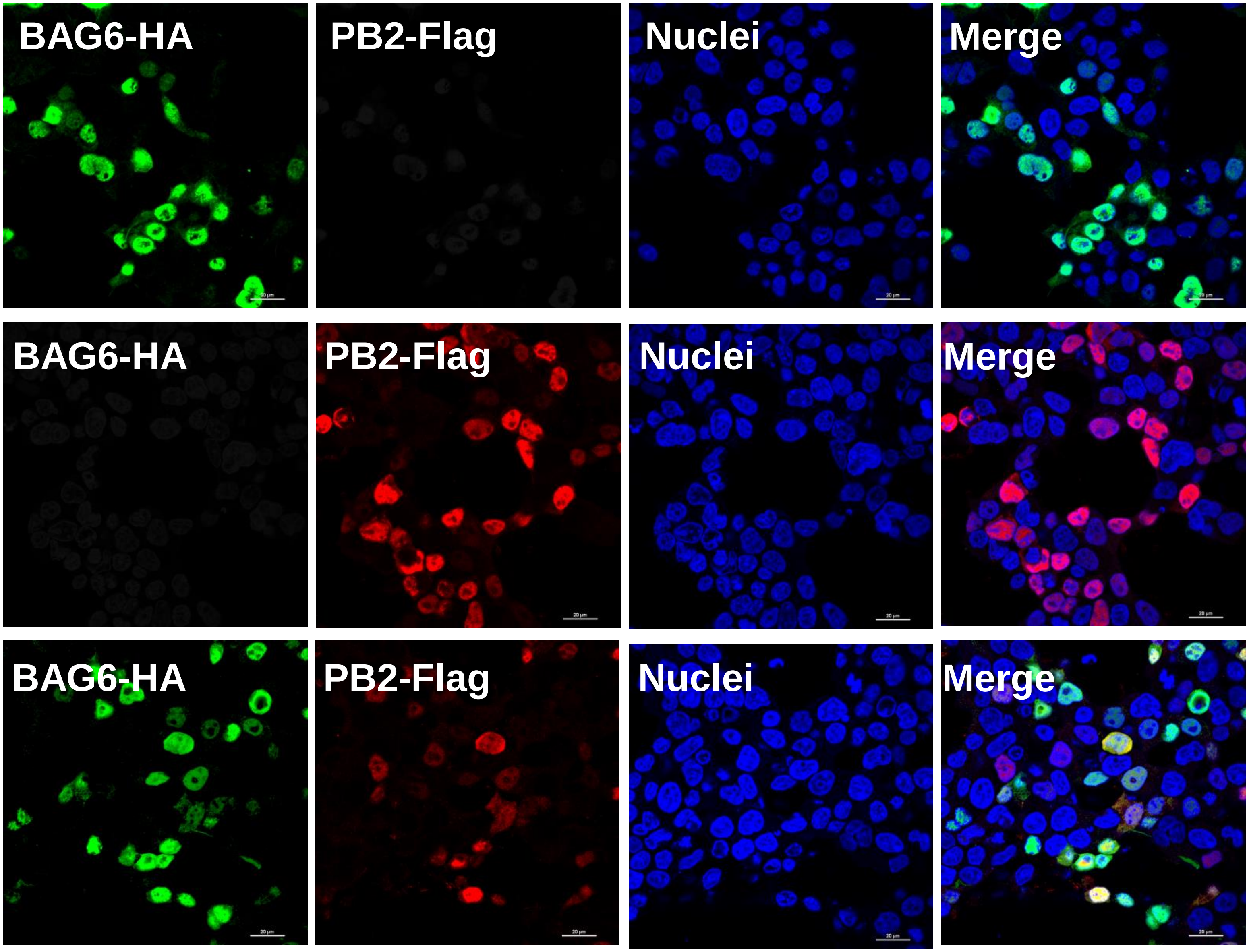
BAG6-HA
PB2-Flag
Nuclei
Merge
BAG6-HA
PB2-Flag
Nuclei
Merge
BAG6-HA
PB2-Flag
Nuclei
Merge

Figure 8D

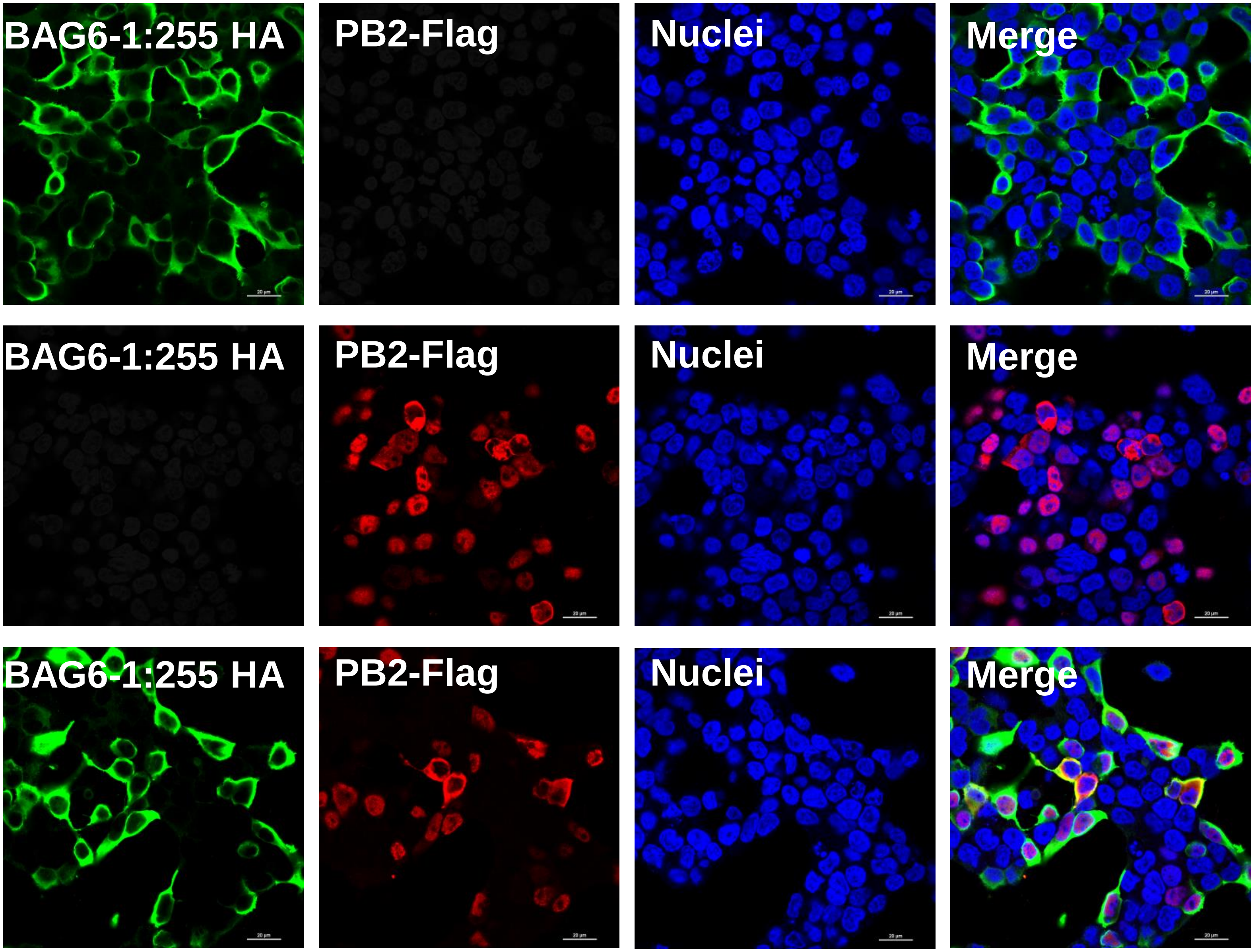
BAG6-1:255 HA
PB2-Flag
Nuclei
Merge
BAG6-1:255 HA
PB2-Flag
Nuclei
Merge
BAG6-1:255 HA
PB2-Flag
Nuclei
Merge
