# Supplementary material for: BAG6 inhibits influenza A virus replication by inducing viral polymerase subunit PB2 degradation and perturbing RdRp complex assembly
Source: PLoS Pathog. 2024 Mar 18;20(3):e1012110. doi: 10.1371/journal.ppat.1012110 (PMC10977894; doi:10.1371/journal.ppat.1012110)

Response to specific comments:

Reviewer #1:

Virus-host interaction is particularly important for the regulation of influenza A virus (IAV) replication and pathogenesis. In the present manuscript, the authors found that overexpression of BAG6 inhibited IAV replication, whereas knockout of BAG6 enhanced IAV replication. By performing luciferase reporter assay, the authors found that BAG6 inhibited the polymerase activity of IAV. Mechanistically, they found that BAG6 suppressed the RdRp activity of IAV through catalyzing the K48-linked ubiquitination and degradation of viral PB2 protein. Meanwhile, the ubiquitin-like (UBL) domain (17-92aa) and PB2-binding domain (124-186aa) of BAG6 were both required for mediating the PB2 degradation and the impairment of virus replication. Notably, BAG6 had obvious antiviral effect in mice. The manuscript can be further improved by addressing the comments below.

Major Issues: Key Experiments Required for Acceptance:

1. Lines 135-136: BAG6 plays an important role in inhibiting the replication of different influenza virus subtypes from avian and human. The authors displayed the anti-viral effect of BAG6 on the replication of H1N1, H7N9, and H9N2 viruses. It will be more informative to determine whether BAG6 also inhibits the replication of highly pathogenic H5N1 virus.

As suggested, the effects of BAG6 on the replication of H5N1(AH1) virus have been determined in both BAG6-overexpression cells (Fig 1G and 1H) and BAG6-KO cells (Fig 2C and 2D).

As shown in Fig 1, the overexpression of BAG6 caused a significant reduction in viral protein expression (Fig 1G) and viral titer (Fig 1H) of H5N1 (AH1). Meanwhile, an increased NP protein level (Fig 2C) and an up to 10-fold higher viral titers (Fig 2D) were observed in BAG6-KO cells infected with H5N1 (AH1) than that in BAG-WT cells.

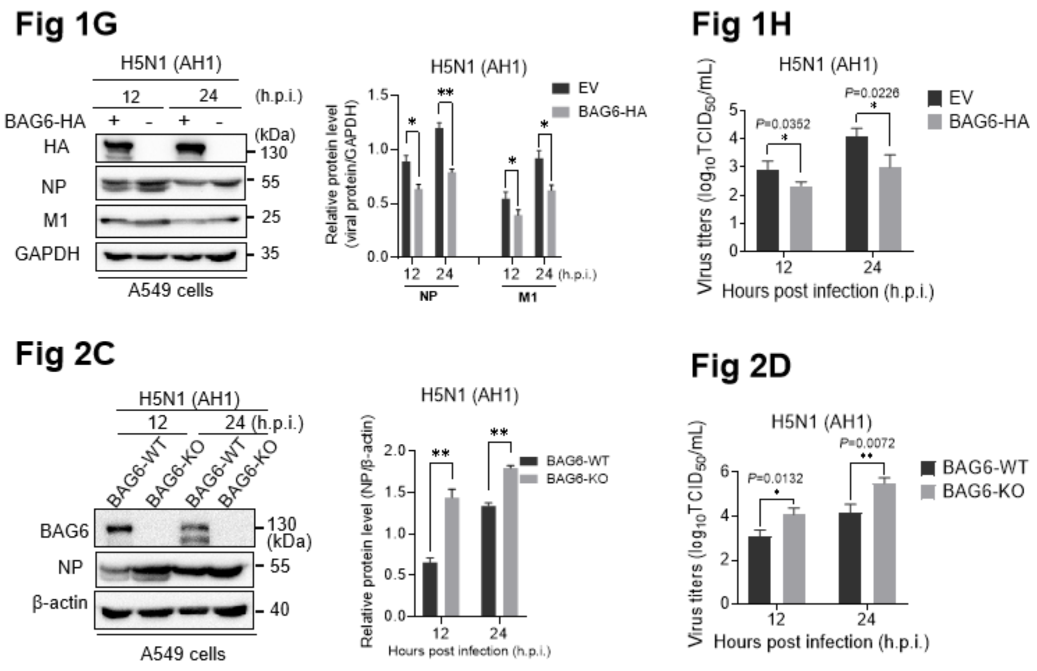

2. In Fig 5B, the expression level of viral M1 protein was very low, and the size of extra nonspecific band was nearly overlapped with that of the viral M1 protein. So it's almost impossible to identify where the actual band of M1 protein is localized?

We are sorry for the imperfect picture because the band of M1 protein is very close to the nonspecific bands of antibodies. We have marked the specific bands of viral proteins (including M1) with red arrows (Fig 6B), hoping they could, to a certain extent, help the readers locate the actual band of M1.

Fig 6B

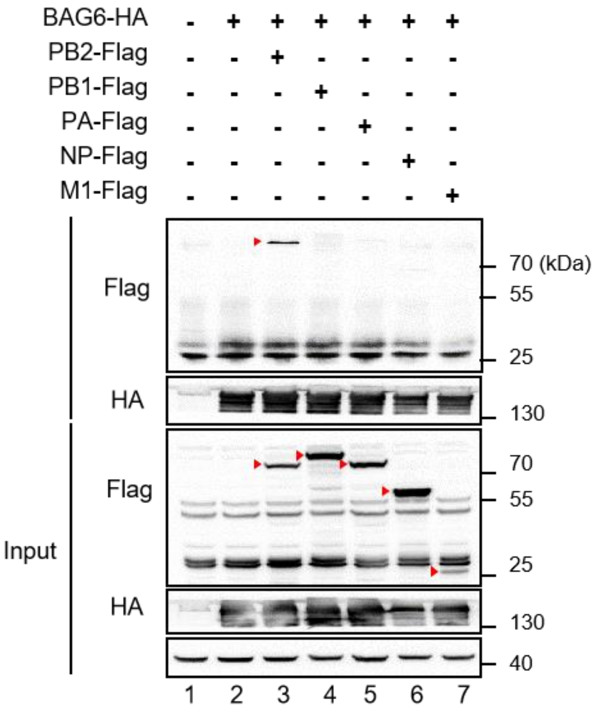

3. In Fig 6, BAG6 induces viral PB2 degradation through K48-linked ubiquitination. It will be highly desired to determine which amino acid residue of PB2 is modified with K48-linked ubiquitination. In such case, whether the mutation of the key amino acid residue in PB2 can prevent PB2 from being degraded by BAG6?

As suggested, we identified the K189 lysine residue on PB2 as the main target for ubiquitination by BAG6 using site-directed mutagenesis (Fig 7F) and determined that the mutation of K189R in PB2 prevented it from being degraded by HA-BAG6 (Fig 7G).

As shown in Fig 7, We generated PB2 mutants containing individual lysine to arginine mutations, and assessed the ubiquitination of each lysine-mutated PB2 proteins in cells transfected with HA-BAG6. The mutation of K189R strongly reduced PB2 ubiquitination upon expression of HA-BAG6 (Fig 7F, lane 11), suggesting that residue K189 is the main target for ubiquitination by BAG6. Consistently, the mutation of K189R in PB2 prevented it from being degraded by HA-BAG6 in comparison with the PB2-WT protein (Fig 7G).

Fig 7F

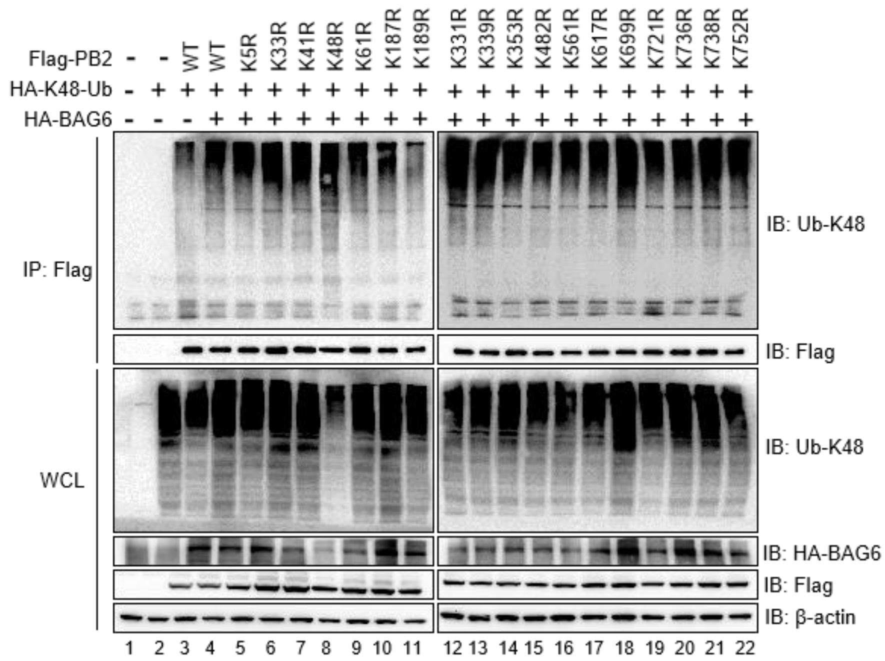

Fig 7G

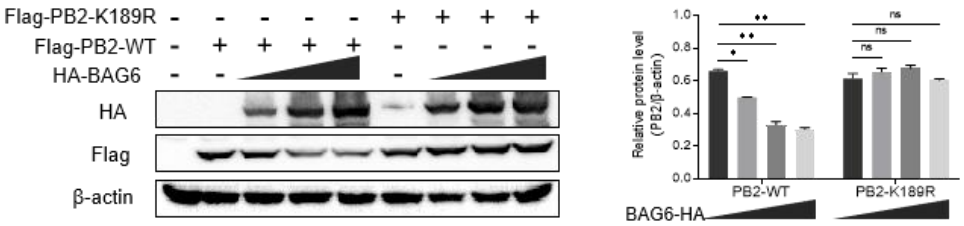

Minor Issues: Editorial and Data Presentation Modifications

1. Lines 113-114: "Consistently, the ectopic expression of BAG6 also caused a significant reduction in viral NP protein expression (Fig 1C) and viral loads (Fig 1D) of H7N9 and H9N2." The authors described the role of BAG6 in virus replication as "significant". The authors had better specifically provide the magnitude of the changes in virus titer. This lack of proper quantitative description of the data obtained is further complicated by the lack of specific numeric data in the figures presented, which makes it very difficult for the reader to estimate the magnitude of the changes observed.

As suggested, we added a proper quantitative description of the changes in virus titer of H7N9, H9N2 and H5N1 in the texts (lines112-114).

2. In all Figures, the abbreviation format needs to be uniform, such as dpi and d.p.i. in Fig 3.

Sorry for the inconsistent formats and we have corrected it in Fig. 3.

3. Line 172, BAG-KO should be BAG6-KO.

Sorry for the mistake, and we have checked the manuscript carefully and corrected the mistakes.

Reviewer #2:

This is an interesting work revealing the role of BAG6 (BCL2-associated athanogene 6) in restricting Influenza A virus (IAV) replication. The IAV is a major public health concern worldwide.

During infection, the viral genome is replicated and translated by the RDRP complex, which consists of PB2, PB1, PA, NP and the vRNA. These polymerases are relatively conserved across the IAV subtypes and are involved in host tropism and host adaptation. In Fig.1, both for the avian and human IAV, overexpression of BAG6 reduces viral translation and virus titer. Simultaneously, in Fig.2, BAG6 KO significantly enhanced virus replication. During this course, the authors found a decrease in BAG6 transcription and translation, and is evident that BAG6 is being downregulated. The authors propose a mechanism of antiviral activity in which the N-terminal region of BAG6 interacts with PB2 to inhibit replication and by targeting PB2 for degradation.

The manuscript is interesting and could be of interest to the field, however, there is already reports in the literature that suggests BAG6 degrades the protein aggregates by K48-linked ubiquitination (PMID: 23275523), and additionally, during RNA virus infection, it negatively regulates RLR-mediated antiviral response by inhibiting the aggregation/ complex formation of the multi-subunit protein complex, virus-induced-signaling adapter (VISA) in the RIG-I pathway, by K48-linked ubiquitination (PMID: 36045679). These manuscripts are not considered and the effects on the innate response are not addressed. Cellular factors might be contributing to the down-regulation of BAG6 so that RIG-I pathway, and this is not ruled out. It is essential that the authors connect if any of the viral proteins are involved in the down-regulation of BAG6 transcription. Mechanistically, the manuscript does not go deep enough to advance the knowledge from previous reports.

Major Issues: Key Experiments Required for Acceptance:

1) The role of type-I IFNs and innate cytokines are not considered, especially in light of previous publications. The mechanism is not studied in depth and the conclusions may not be supported without further investigation.

Thanks for the sound and constructive suggestion. To determine if IFN-I innate immune pathway is involved in BAG6-mediated anti-IAV response, we try to perform the following experiments:

1. We firstly determined whether BAG6 could also regulate RIG-I-MAVS mediated innate immune pathway during IAV infection. The expressions of several key components involved in IFN-I signaling pathways, including pattern recognition receptor RIG-I, the phosphorylated STAT1 (P-STAT1) and the IFN-stimulated gene ISG15, were examined in both BAG6 overexpressing and knocking-out HeLa cells during PR8 infection. The results showed that the expressions of RIG-I, P-STAT1 and ISG15 were only slightly increased in BAG6-overexpressing cells (Fig 4A), and exhibited a comparatively obvious decrease in BAG6-deficient cells (Fig 4B).

Fig 4A

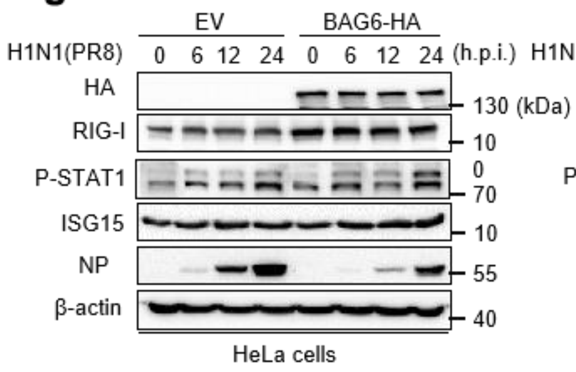

Fig4B

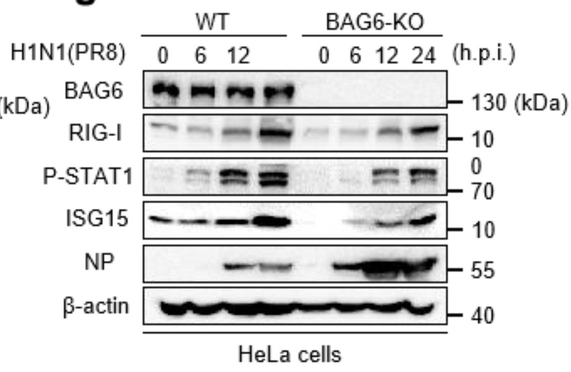

These data seem to be contradictory to the findings reported in previous study (PMID: 36045679). However, considering the potential impacts of the influenza virus itself on IFN pathway (as BAG6 could regulate IAV replication, and numerous studies have reported that IAVs could escape innate antiviral response by inhibiting IFN-I activation), we next conducted the following experiments.

2. To verify whether BAG6 inhibits IAV replication through IFN-I innate immune pathways, we used an IRF9-knockout HeLa cell line (IRF9-KO), in which the IFN-I-mediated innate immune responses were disrupted by knocking out interferon regulation factor 9 (IRF9) in JAK-STAT pathway, to examine the effect of BAG6-HA on IAV replication. Interestingly, while the depletion of IRF9 completely inhibited the expression of IFN-I-induced antiviral factor ISG15 in PR8-infected cells, BAG6-HA still remained its ability to suppress the expression of viral NP protein (Fig 4C) and induced an up to 10-fold reduction in the virus titer (Fig 4D), which are equal to the reduced levels in HeLa-WT cells. These data suggest that BAG6 inhibits IAV replication independent of IFN-I-mediated innate immune response.

Fig 4C

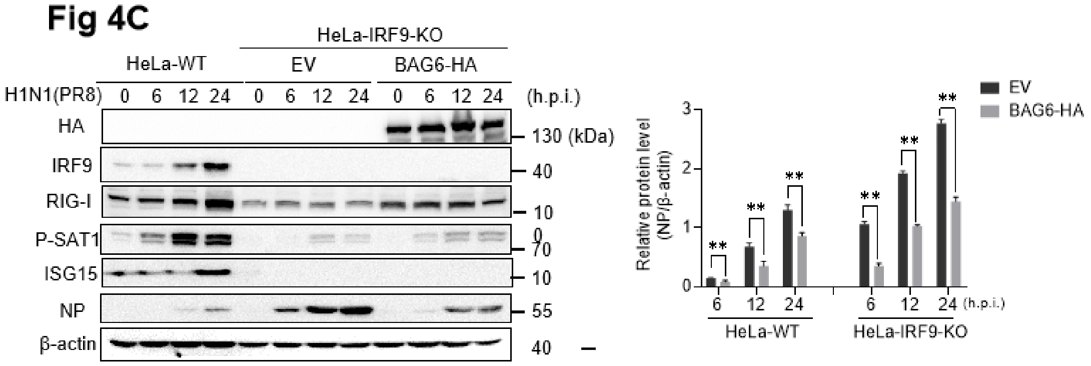

Fig 4D

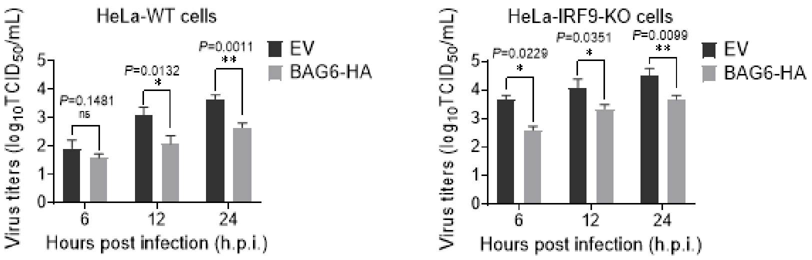

3. To further verify the above findings, we also examined the inhibitory effect of BAG6 on PR8

replication in Vero cell line, since it has been reported as a cell line with a deficient interferon-mediated antiviral response. As shown in Fig 4E and 4F, the overexpression of BAG6-HA could also significantly inhibit PR8 replication in Vero cells.

Fig 4E

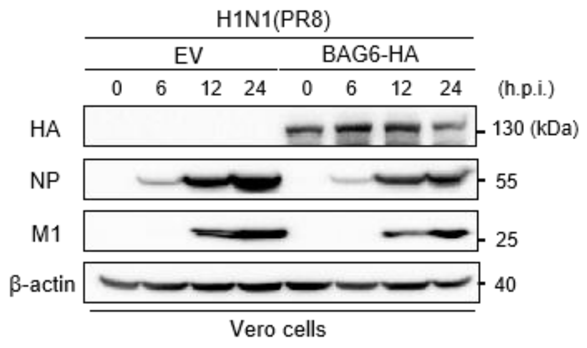

Fig 4F

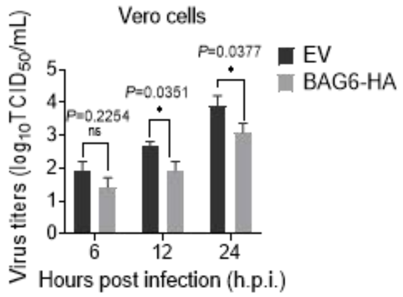

Altogether, these findings indicate that BAG6 is able to inhibit IAV replication independent of IFN-I-mediated innate immune response, and we thus speculate that the mild regulation of IFN-I immune activation presented in BAG6 overexpressing and knockout cells (Fig 4A and 4B) may be due to an indirect effect of BAG6 on virus replication (We have already discussed it in Results section).

2) Figure 1. overexpression of BAG6 may result in immune activation in A549 cells. Type-I IFNs needs to be measured.

As mentioned above, we determined whether overexpression of BAG6 may result in the immune activation of IFN-I pathway by monitoring RIG-I, P-STAT1 and ISG15. As shown in Fig 4A, the overexpression of BAG6 could only slightly increase the expression of these key components involved in IFN-I pathways, which could hardly cause a significant reduction in IAV replication. Therefore, we speculate that it may be due to an indirect effect of BAG6 on IAV replication.

3) Figure 2, the same as in point 2 above. What are the effects of knockout in cytokines, IFNs and ISGs?

The same as above, we examined the effect of BAG6 knockout in RIG-I, P-STAT1 and ISG15 (Fig 4B).

4) Figure 3. PPMOs could be also inducing immune activation. Controls need to include measuring IFNs and ISGs.

As suggested, we examined the effect of PPMO-NC and PPMO-BAG6 on the expression of RIG-I, P-STAT1 and ISG15, and found that there is no obvious activation of IFN-I pathway by BAG6 (data not shown in the manuscript).

Data not shown

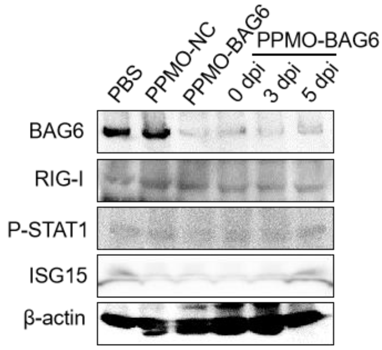

5) Figure 4 needs to show control expression of all transfected proteins

As suggested, the expression of all transfected proteins including HA-BAG6, PB2, PB1, PA and NP were measured by western blotting in Fig 5.

Fig 5

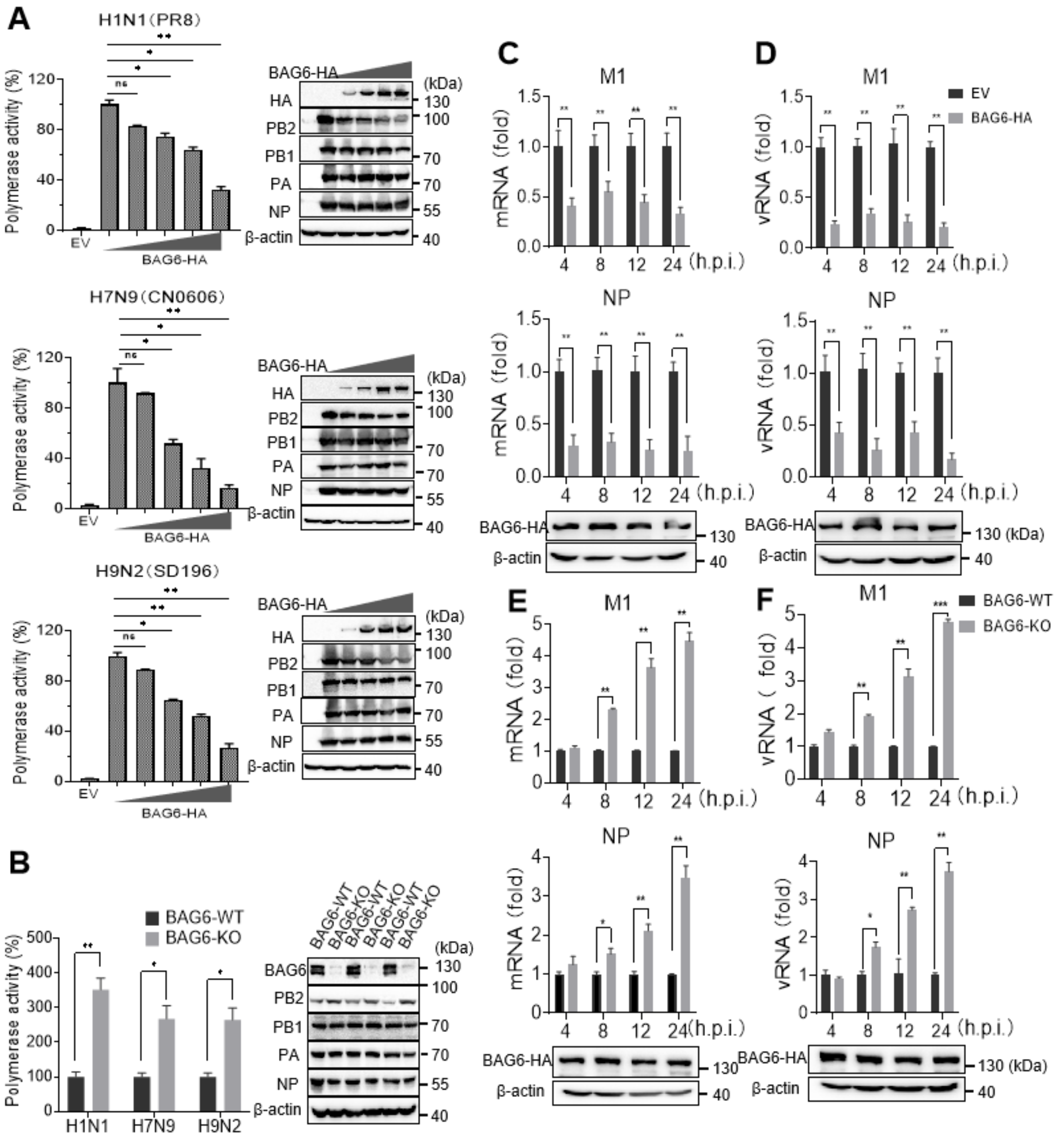

6) Figure 5, all IPs are done using wrong controls. Figure 5A, the proper control for the IP is the absence of BAG6-HA in the presence of PR8 infection. This is to ensure that there is no unspecific binding of the viral proteins to the beads. The same in Figure 5B, a control is needed without BAG6-HA in the presence of overexpressed Flaf proteins (at least PB2).

This is indeed a weak point in our experimental design, and we have repeated the IP assays in the original Fig 5A by adding the proper controls as shown in Fig 6A. Also, the interaction between exogenous BAG6-HA and PB2-Flag were verified by an additional IP with the proper controls in Fig 6C.

Fig 6A

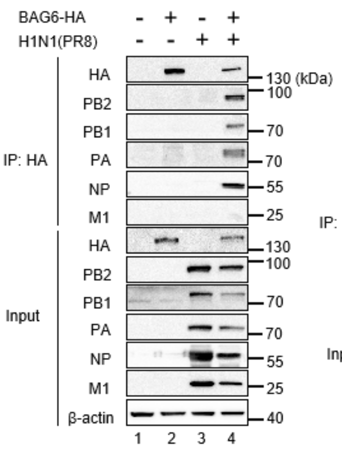

Fig 6B

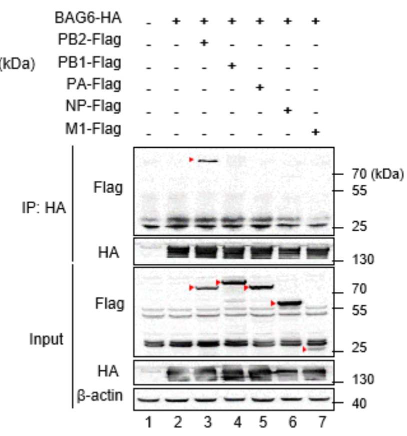

Fig 6C

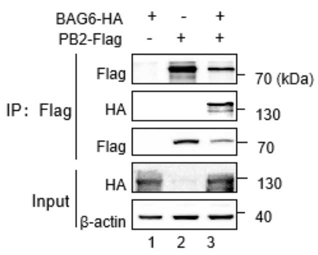

7) Figure 5C lacks controls, each protein needs to be overexpressed separately. Also, additional panels with more cells need to be shown.

As suggested, the immunofluorescence stainings were performed in cells expressed BAG6-HA or PB2-Flag separately, or in cells co-expressed both BAG6-HA and PB2-Flag. Meanwhile, more cells were displayed in the same field of vision.

As shown in Fig 6E, while the PB2 alone mainly localized in the nuclei, BAG6 could co-localize with PB2 and promote its localization in both nuclei and cytoplasm of cells.

Fig 6E

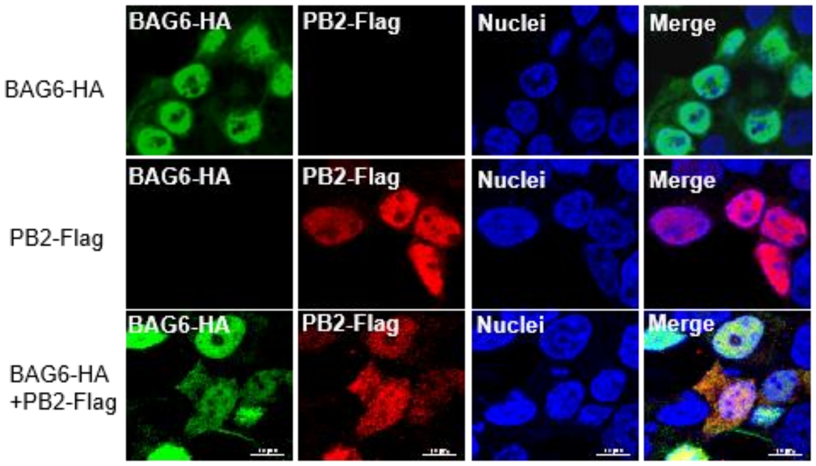

8) Figure 6C and D need to be compared to cells without MG132 as control. Also these two experiments need to be repeated without HA-Ub overexpression and instead immunoblotting for endogenous ubiquitin with anti-Ub specific antibodies.

According to our experience (PMID: 31647545 and 23728348) and the previous reports from others (PMID: 36271046 and 32265326), the levels of ubiquitination are barely detectable without MG132 treatment, because the protein substrates are quickly degradated after ubiquitination modification. Also it is difficult to monitor endogenous ubiquitin due to its relatively low quantity (compared to exogenous labeled Ub) and instantaneous interaction with substrates.

9) Figure 7D needs controls with individual proteins.

As suggested, the immunofluorescence stainings were performed in cells expressed BAG6-1:255 HA or PB2-Flag separately, or in cells co-expressed both BAG6-1:255 HA and PB2-Flag.

As shown in Fig 8D, the PB2-Flag alone mainly localized in the nuclei, but the N-terminal truncation of BAG6 (BAG6 1:255-HA), which lacks a nuclear localization signal (NLS), could co-localize with PB2 and induce its accumulation in the cytoplasm, suggesting that BAG6 1-255aa is sufficient for interacting with PB2 and might be responsible for targeting it for cytoplasm accumulation.

Fig 8D

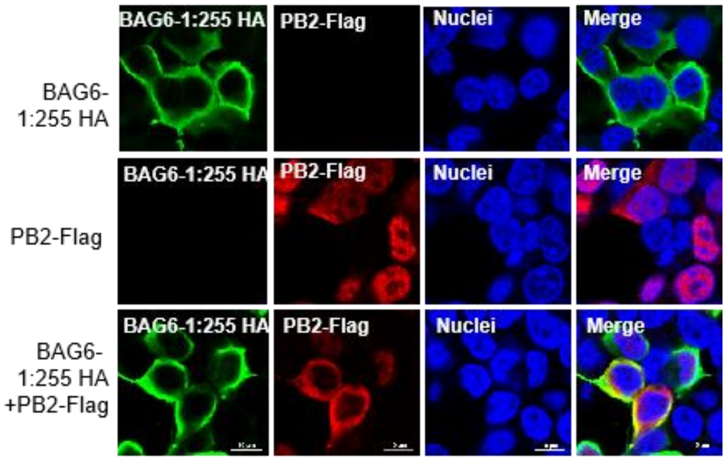

10) Fig.6, it is essential to show the rescue of PB2 degradation under MG132 treatment in infection conditions. Also, check the PB1 and NP levels under MG132 treatment since it was found to pulled down with BAG6 in Fig. 5A.

As suggested, we examined the rescue of PB2 degradation by MG132 treatment under the H1N1(PR8) infection conditions. As shown in Fig 7C, the reduced PB2 protein levels by BAG6-HA in PR8-infected cells were restored to the control level by the treatment of MG132, but not by lysosome inhibitor Chloroquine (CQ), verifying that BAG6 induces proteasome degradation of PB2 during virus infection.

Fig 7C

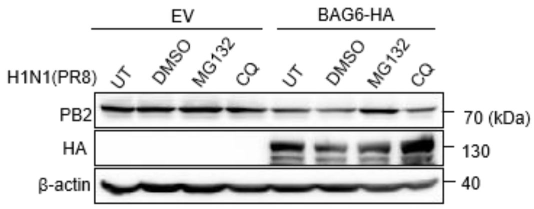

In addition, as shown in Fig 5A (right panels), the overexpression of HA-BAG6 can only induce the degradation of PB2, but not other viral proteins (PB1, PA and NP), from different IAV subtypes. Thereby, it is not necessary to check the PB1 and NP levels under MG132 treatment since they are not degradated by BAG6. Also we have already described this results in lines 209-215.

Fig 5A

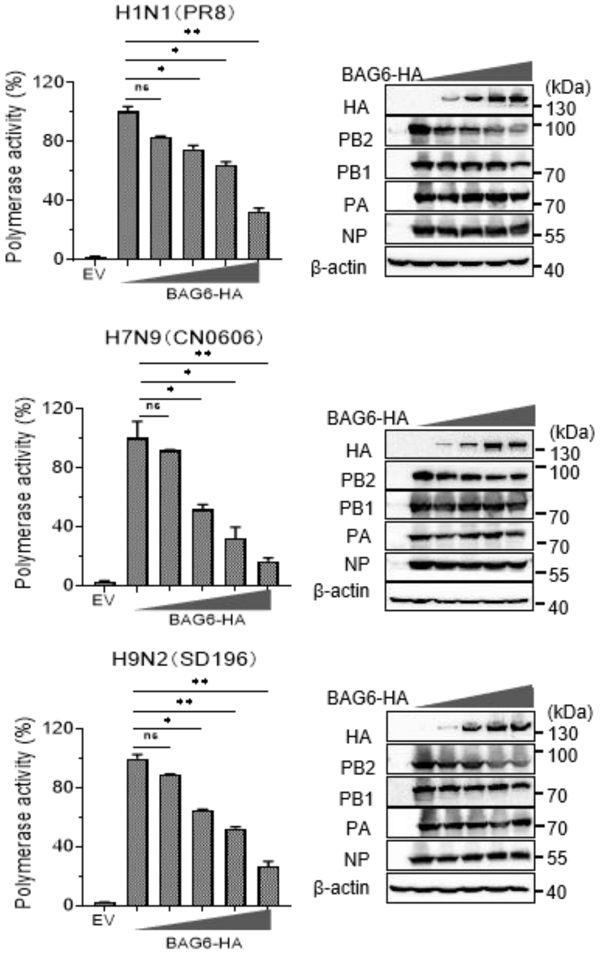

11) Fig.7, It will be appropriate to verify the effect of N-terminus BAG6 (1-225 aa) in the BAG6 KO cells under H1N1 infection.

As suggested, we examined the effect of N-terminus BAG6 (1-225 aa) on viral protein expression and virus titers in BAG6-KO cells under the H1N1(PR8) infection conditions. As shown in Fig 8, the viral protein expression (PB2 and NP) (Fig 8H) and virus titers (Fig 8I) up-regulated in BAG6-KO cells were restored to the control levels (BAG6-WT) by BAG6 1:255-HA overexpression. These results verified that BAG6 blocks IAV replication via its N-terminal 1-255aa region.

Fig 8H

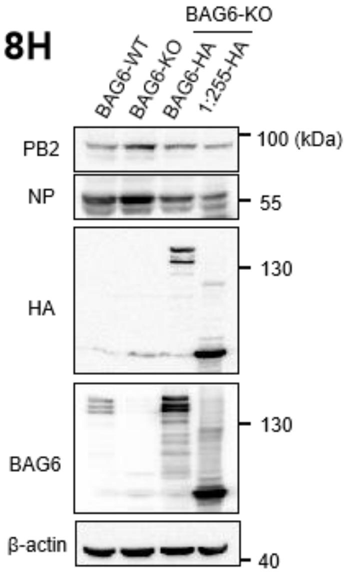

Fig 8I

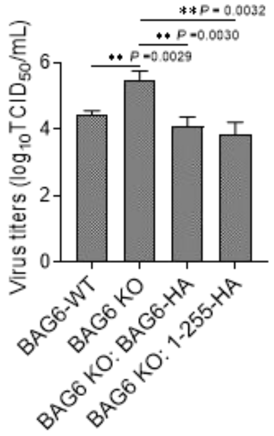

Minor Issues: Editorial and Data Presentation Modifications

1) Figure 3 also needs additional controls with PPMOs but without infection. The same for the IHCs in E and F, needs controls without infection.

As suggested, an additional control with PPMOs but without PR8 infection (PPMO-BAG6-Mock) were added in the mice experiments and the results are shown in Fig 3.

Knocking down BAG6 alone (PPMO-BAG6-Mock) did not affect the weight and survival rate of mice (Fig 3C and 3D). Also, the PPMO-BAG6-Mock control were performed the immunohistochemical staining of viral protein (Fig 3F) and histopathological analysis of lung tissue (Fig 3G).

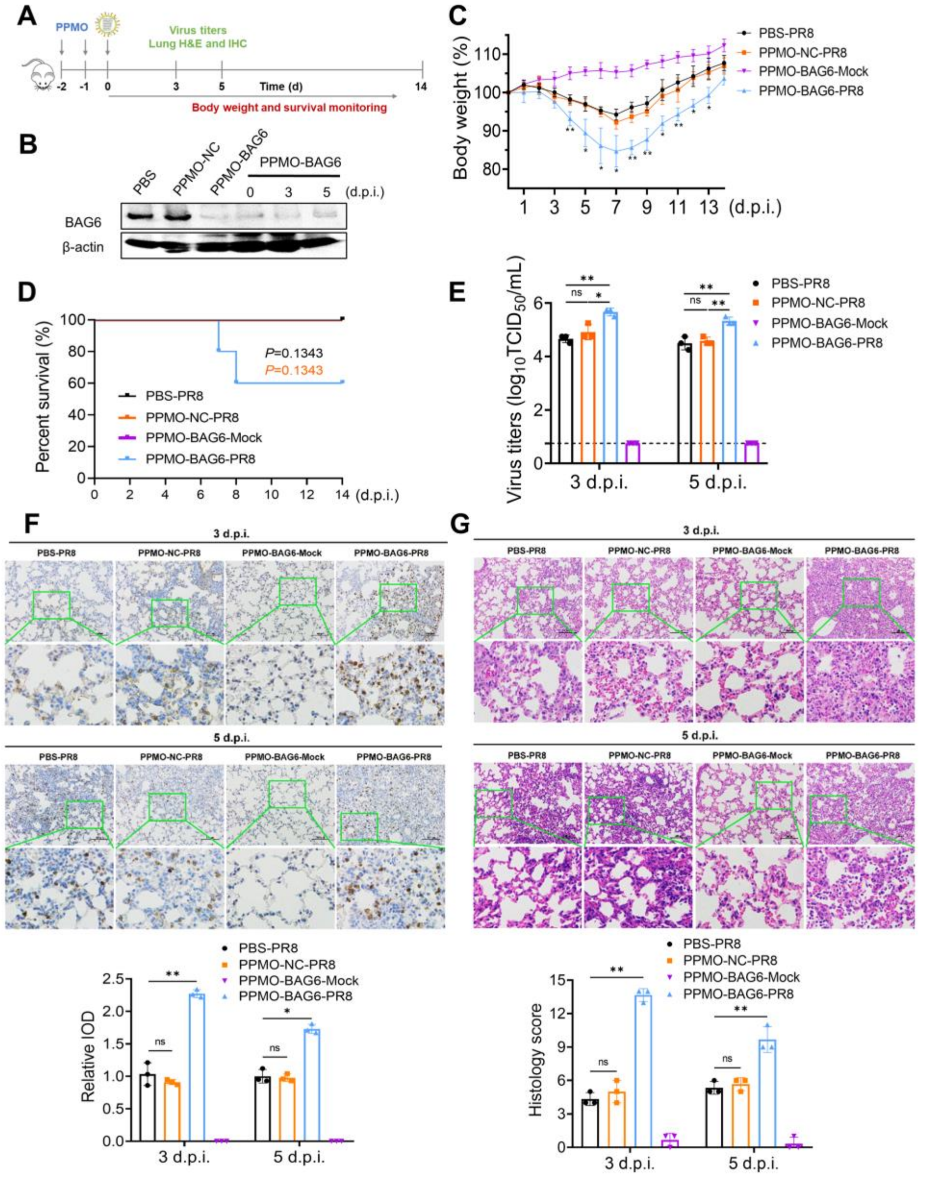

2) In Fig.1 the authors can also check the densitometry of NP/M in the western blot to see if any correlation appears between the IAV subtypes in the presence/absence of BAG6 overexpression.

As suggested, the densitometry of both NP and M1 in different IAV subtypes were detected in the western blotting. In addition, we also examined the effect of BAG6 on the replication of H5N1(AH1) virus.

As shown in Fig 1, the overexpression of BAG6 caused a significant reduction in both NP and M1 protein expression in all IAV subtypes, including H1N1 (Fig 1A), H7N9 (Fig 1C),H9N2 (Fig 1E), and H5N1 (Fig 1G).

Fig 1

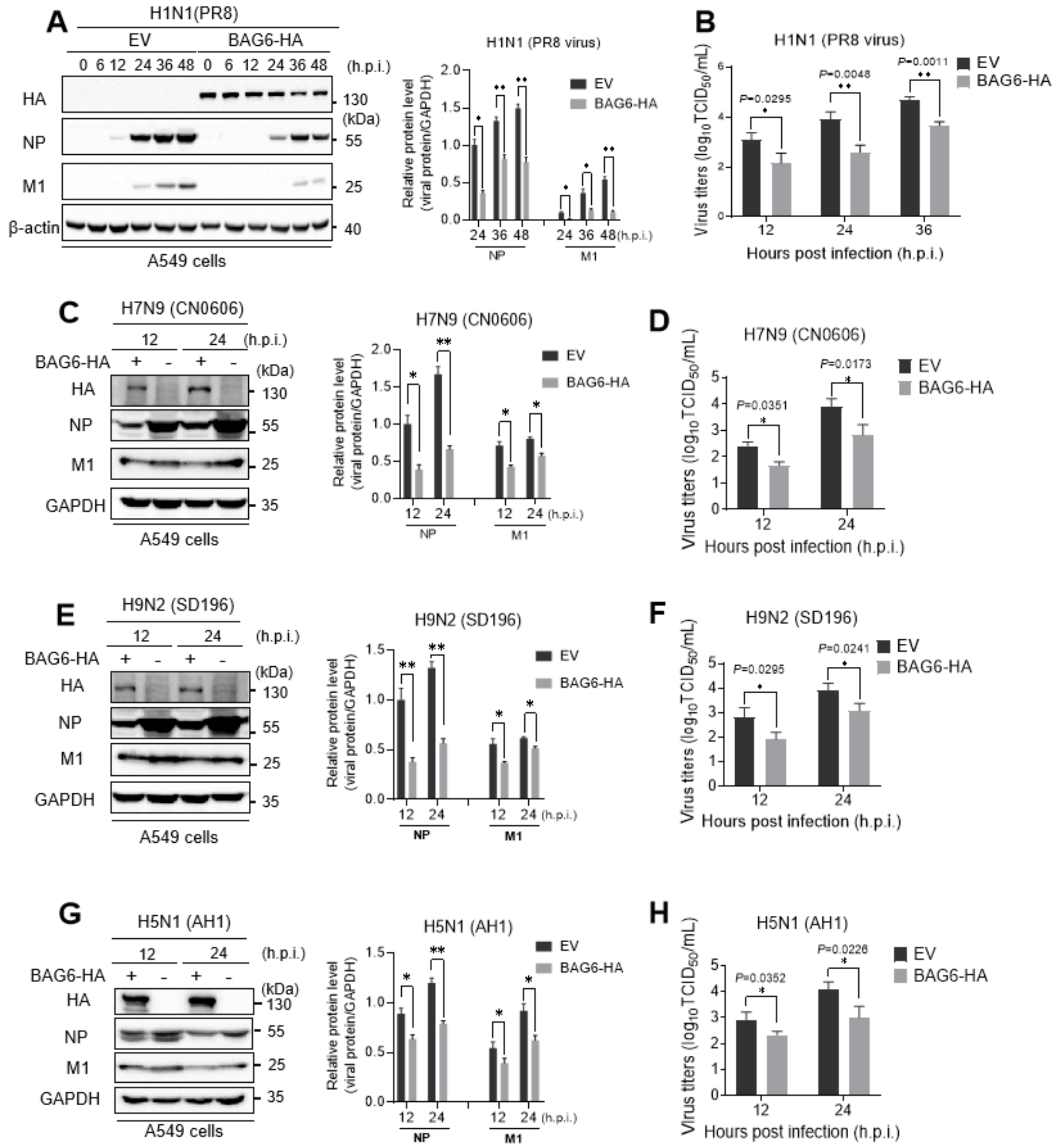

3) The authors can show the expression of RIG-I in the blot (Fig. 2A, BAG6-wt and KO and H1N1 infection).

As suggested, the expression of RIG-I were shown in Fig 4A and 4B.

4) For H1N1 infection in the PBS, PPMO-NC, and PPMO-BAG6 treated groups (Fig. 3, panel E), we see an appreciable and statistically significant viral titer on day 3 and as expected, it goes down on day 5. It will be informative if the authors can show the expression level of BAG6 on days 3 and -5 in these tissue samples.

We attempted to examine the expression of BAG6 in these tissue samples by IHC staining, but unfortunately, the available BAG6 antibodies cannot effectively monitor the BAG6 protein in mouse tissues. Therefore, we examined the expression levels of BAG6 on 3 and 5 days post infection in

the lung tissues by Western blotting (as shown in Fig 3B). Hopefully it would be helpful.

Fig 3B

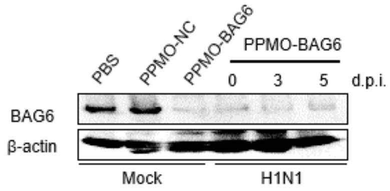

5) Check the nucleotide sequence of Uni-12 Primer and highlight the source/ reference.

The nucleotide sequence of Uni-12 primer was corrected as "5′-AGCAAAAGCAGG-3′", and the source was highlighted as reference [60].

6) Fig.5 Panel A, under PR8 infection, BAG6 interacts with the PB2, PB1 and NP. PB2 and NP are pulled down, almost in equal amounts, with BAG6. How do you explain the discrepancy with Fig.5B (where only PB2 interacts with BAG6)? It could also be possible that the association of polymerase complex (PB2, PB1, PA and NP) on the vRNA is sensing the BAG6 and hence you get an interaction in Fig.5A.

Yes, it is possible that the viral polymerase subunit proteins are immunoprecipitated by BAG6 through an indirect binding with PB2 on the vRNP complex. Thus, to clarify this possibility, we performed immunoprecipitation assay in A549 cells co-transfected with BAG6-HA and PB1-Flag, or PB2-Flag, or PA-Flag, or NP-Flag, respectively. The results showed that BAG6-HA was only able to interact with PB2-Flag, but not with PB1-Flag, PA-Flag, or NP-Flag individually (Fig 6B, the original Fig 5B). Also, we have explain the logical relation between the experiments shown in Fig 6A and Fig 6B in lines 230-235.

7) Lines 139-140: BAG6-deficient mice were generated using a peptide-conjugated phosphorodiamidate morpholino oligomers (PPMOs)". This sentence is incorrect. These are not deficient mice, this is a knockdown. Please correct text.

The inaccurate description have been corrected in whole manuscript.

8) Lines 189-190:. The M1-Flag was used as a negative control. The results showed that BAG6-HA was only able to directly interact with PB2-Flag (Fig 5B, lane 3)…" . these experiemnst don't whow direct interactions. Purified proteins would be needed in in vitro binding assays. Please remove 'directly' and discuss the potential for indirect interactions.

As suggested, we have removed 'directly' and discussed the possibility of indirect interactions.

Reviewer #3:

The manuscript by Zhou et al describes the role of BAG6 (BCL2-associated athanogene 6) as restriction factor in influenza virus infection. Overexpression of BAG6 leads to about a log drop in viral titer, while knockout of BAG6 increase viral titers by about a log in A549 and Hela cells. The authors used morpholinos to knock down BAG6 expression in mice, and found that this led to increased viral titers, weight loss and death, whereas no effects were seen in the PBS-infected BAG6 knock down mice or the infected wt mice. Finally, the authors used pull-down assays to demonstrate that BAG6 binds to the IAV RNA polymerase subunit PB2 and that it can induce the degradation of

PB2. The authors propose that BAG6 interacts with the N-terminus of PB2 and interferes with RNA polymerase assembly or induces PB2 degradation. Generally, the manuscript is well-organized, but there are many grammatical issues (e.g. line 845 "A549 cells was transfected"), and the data appear to support the conclusions. There are a couple of points that would need attention.

Minor Issues: Editorial and Data Presentation Modifications

1. Line 150: please clarify "extremely higher"

Thanks for the suggestion. We have clarified the description by adding a proper quantitative description of the changes in virus titer in the texts (lines153-155).

2. Figure 7B/C: The figure legend appears to be incorrect as it refers to the IP flag as Fig. 7C. This should be 7B, and the HA IP should be 7C.

Sorry for the mistakes, and we have corrected it in the legends of Figure 8B/C (the original Figure 7B/C).

3. Figure 7C: the IP has two Flag blots. Should the bottom one be part of the input?

Yes, it was wrongly labeled. Sorry about that and we have corrected it it the Figure 8C (the original Figure 7C).

4. In Fig. 7D, the 1:255-HA constructs keeps PB2 in the cytoplasm, suggesting that it affects PB2 import into the nucleus and the functioning of the PB2 NLS (which resides near the C-terminus of PB2). The authors only consider the BAG6 NLS in their discussion. In addition, it would be useful if the authors added controls to show/confirm the localization of PB2 and BAG6 alone.

Thanks for the suggestion, and we have performed the immunofluorescence stainings in cells expressed BAG6 or PB2 separately, or in cells co-expressed these proteins.

As shown in Fig 6E, while the PB2 alone mainly localized in the nuclei, BAG6 could co-localize with PB2 and promote its localization in both nuclei and cytoplasm of cells.

In Fig 8D, the PB2-Flag alone mainly localized in the nuclei, but the N-terminal truncation of BAG6 (BAG6 1:255-HA), which lacks a nuclear localization signal (NLS), localized in the cytoplasm and could induce the PB2 accumulation in cytoplasm, suggesting that BAG6 1-255aa is sufficient for interacting with PB2 and might be responsible for targeting it for cytoplasm accumulation.

Fig 6E

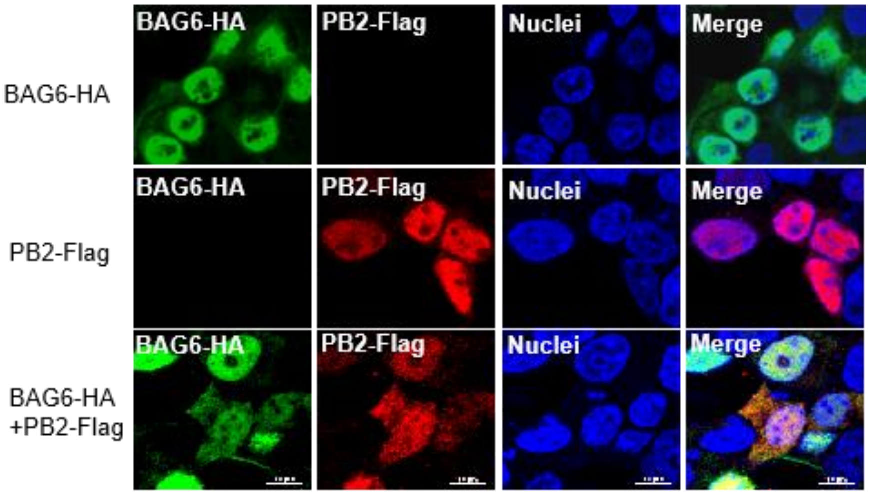

Fig 8D

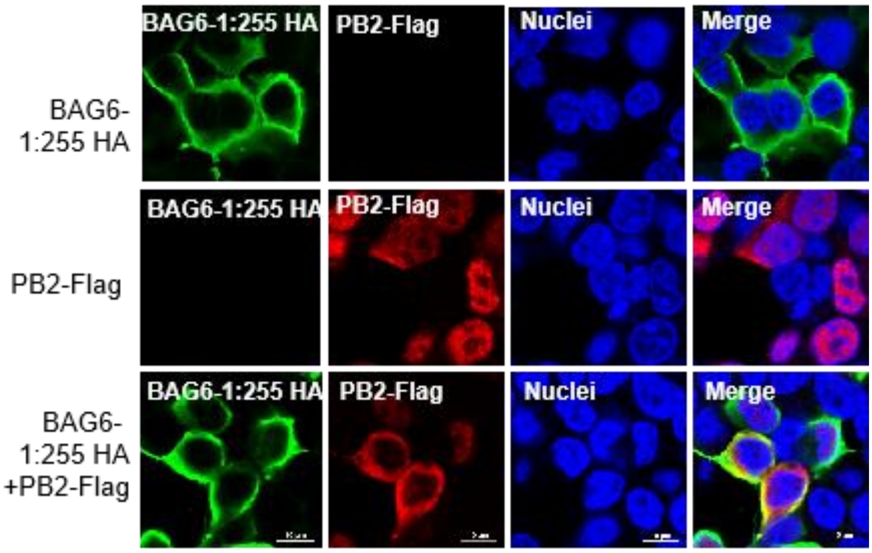

5. Line 242. "Targeting for subcellcular localization" appears to be missing a word (e.g., cytoplasmic).

We have changed this sentence as "BAG6 1-255aa is sufficient for interacting with PB2 and might be responsible for targeting it for cytoplasm accumulation" (lines 296-298).

6. The authors claim that BAG6 binds to the PB2 N-terminus, but they provide no PB2 mutational data (e.g. PB2 truncations) to support this. The language should be adjusted.

We performed a Co-IP assay between BAG6 and the N-terminal truncation of PB2 (amino acids 1-247), and found that BAG6 could bind to the N-terminus of PB2 protein (Fig 6F).

Additionally, we further identified the K189 lysine residue on the N-terminus of PB2 as the main target for ubiquitination by BAG6 using site-directed mutagenesis (Fig 7F) and determined that the mutation of K189R in PB2 prevented it from being degraded by HA-BAG6 (Fig 7G). Hopefully,

these additional data could support our conclusions.

Fig 7F

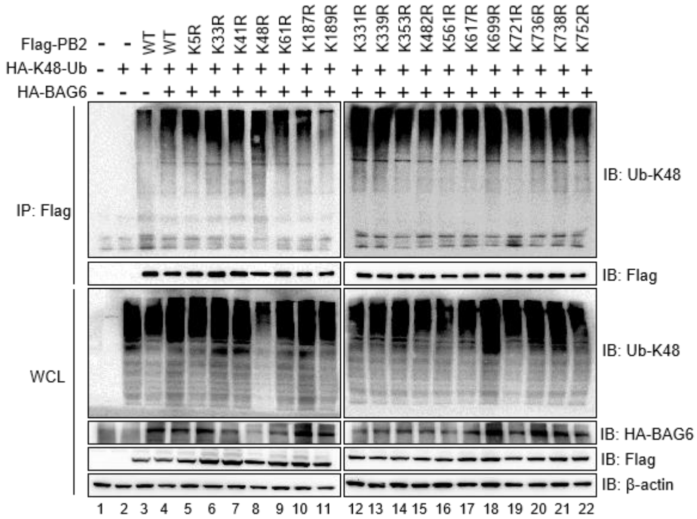

Fig 6F

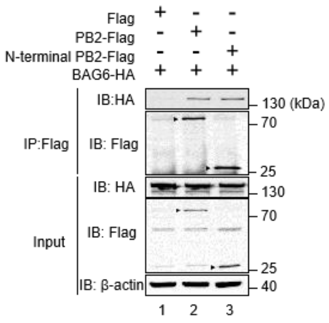

Fig 7G

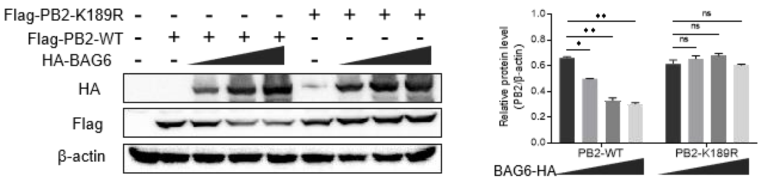

Supplement: Attachment — Submitted filename: Response to reviewers comments.pdf [file ppat.1012110.s008.pdf]
